# Socioeconomic status and non-communicable disease behavioural risk factors in low-income and lower-middle-income countries: a systematic review

**DOI:** 10.1016/S2214-109X(17)30058-X

**Published:** 2017-02-11

**Authors:** Luke Allen, Julianne Williams, Nick Townsend, Bente Mikkelsen, Nia Roberts, Charlie Foster, Kremlin Wickramasinghe

**Affiliations:** a British Heart Foundation Centre on Population Approaches for Non-Communicable Disease Prevention, Nuffield Department of Population Health, University of Oxford, Oxford, UK; b Health Care Libraries, Bodleian Libraries, University of Oxford, Oxford, UK; c WHO Global Coordination Mechanisms on the Prevention and Control of Non-communicable diseases, WHO, Geneva, Switzerland

## Abstract

**Background:**

Non-communicable diseases are the leading global cause of death and disproportionately afflict those living in low-income and lower-middle-income countries (LLMICs). The association between socioeconomic status and non-communicable disease behavioural risk factors is well established in high-income countries, but it is not clear how behavioural risk factors are distributed within LLMICs. We aimed to systematically review evidence on the association between socioeconomic status and harmful use of alcohol, tobacco use, unhealthy diets, and physical inactivity within LLMICs.

**Methods:**

We searched 13 electronic databases, including Embase and MEDLINE, grey literature, and reference lists for primary research published between Jan 1, 1990, and June 30, 2015. We included studies from LLMICs presenting data on multiple measures of socioeconomic status and tobacco use, alcohol use, diet, and physical activity. No age or language restrictions were applied. We excluded studies that did not allow comparison between more or less advantaged groups. We used a piloted version of the Cochrane Effective Practice and Organisation of Care Group data collection checklist to extract relevant data at the household and individual level from the included full text studies including study type, methods, outcomes, and results. Due to high heterogeneity, we used a narrative approach for data synthesis. We used descriptive statistics to assess whether the prevalence of each risk factor varied significantly between members of different socioeconomic groups. The study protocol is registered with PROSPERO, number CRD42015026604.

**Findings:**

After reviewing 4242 records, 75 studies met our inclusion criteria, representing 2 135 314 individuals older than 10 years from 39 LLMICs. Low socioeconomic groups were found to have a significantly higher prevalence of tobacco and alcohol use than did high socioeconomic groups. These groups also consumed less fruit, vegetables, fish, and fibre than those of high socioeconomic status. High socioeconomic groups were found to be less physically active and consume more fats, salt, and processed food than individuals of low socioeconomic status. While the included studies presented clear patterns for tobacco use and physical activity, heterogeneity between dietary outcome measures and a paucity of evidence around harmful alcohol use limit the certainty of these findings.

**Interpretation:**

Despite significant heterogeneity in exposure and outcome measures, clear evidence shows that the burden of behavioural risk factors is affected by socioeconomic position within LLMICs. Governments seeking to meet Sustainable Development Goal (SDG) 3.4—reducing premature non-communicable disease mortality by a third by 2030—should leverage their development budgets to address the poverty-health nexus in these settings. Our findings also have significance for health workers serving these populations and policy makers tasked with preventing and controlling the rise of non-communicable diseases.

**Funding:**

WHO.

## Introduction

Non-communicable diseases account for 70% of global deaths,^1^ and the disproportionate concentration of premature deaths from these diseases in lower-income countries has been described as “the social justice issue of our generation”.^2^, ^3^ The Sustainable Development Goals (SDGs) include the target of reducing premature deaths from non-communicable diseases by a third over the next 15 years.^4^ The disconnect between non-communicable disease prevention, development, and poverty reduction strategies was mentioned by WHO in the first Global Action plan in 2008,^5^, ^6^, ^7^ with calls for improved coordination culminating in two “Non-communicable diseases and Development Cooperation” dialogues in 2015.^8^

Development agencies—mainly working with the poorest members of low-income and lower-middle-income countries (LLMICs)—might be more likely to realign their activities to address non-communicable disease prevention if there was clear evidence that non-communicable diseases and their risk factors affect these populations.^8^ The distribution of diseases and risk factors between nations is well established, but little evidence for the socioeconomic distribution of risk factors within LLMICs has been published.^9^ The urgent need for disaggregated data was underlined at the 2011 UN High Level Meeting on non-communicable diseases.^10^

**Research in context**

**Evidence before this study**

We searched PubMed and Google scholar on July 28, 2015, with no language restrictions. Our search terms were a list of World Bank-defined low-income and lower-middle income countries (LLMICs); MeSH and free-text terms for tobacco use, alcohol use, diet, and physical inactivity; and socioeconomic status. Studies published before 1990 were excluded. There was a moderate risk of bias among the included studies.

From this search, we identified a 2005 non-systematic review of surveys from 11 low-income and middle-income country (LMIC) WHO subregions and a meta-analysis of studies examining tobacco use and income. These studies report higher prevalence of tobacco use (odds ratio 1·48, 95%CI 1·38–1·59) and lower alcohol use in the poorest strata of LMICs compared with more affluent groups. The most comprehensive analysis to date comes from an analysis of LMIC World Health Survey data from 2002–04. Self-reports from 232 056 participants from 48 countries—of which 25 were upper-middle-income and 23 were low-income or lower-middle income (LLMICs)—suggested that those with more education and assets were more likely to be physically inactive and consume insufficient fruit and vegetables and less likely to smoke daily. The socioeconomic patterning of heavy episodic drinking was mixed and inequalities were more pronounced in the least developed countries. The findings from that study are now 10 years old, and largely drawn from upper-middle or high-income countries.

**Added value of this study**

To our knowledge, this study is the first systematic review to examine the distribution of the main non-communicable behavioural risk factors across different socioeconomic groups within LLMICs and the first study to report on physical activity and socioeconomic status in developing countries. This work supports ongoing efforts to link non-communicable disease prevention with the global development agenda and provides evidence for development agencies on how to engage with non-communicable diseases. Our study shows that lower socioeconomic groups are more likely to drink alcohol, use tobacco, and consume insufficient fruit and vegetables than more advantaged groups. Higher socioeconomic groups were found to be more inactive and might consume more fats, salt, and processed food. Our findings substantially augment the scant evidence from previous LLMIC-based reviews on individual risk factors. With the use of broader measures of socioeconomic status, we found significant differences between castes, classes, sexes, and occupational groups with the widest differences observed across different educational strata.

**Implications of all the available evidence**

Combined with previous work, the association between non-communicable disease risk factors and socioeconomic status seems to be dependent on setting, population, and exposure definitions. Tobacco use seems to be almost universally more prevalent in low socioeconomic groups than in high socioeconomic groups, whereas alcohol and diet require further investigation. Our findings have importance for the development community that have a part to play in ensuring that their projects do not promote environments that promote non-communicable diseases in low-income settings. This study shows that there is a clear socioeconomic gradient of non-communicable disease risk behaviours within most LLMICs. Education was strongly correlated with healthier behaviour in most settings and might be an important tool in controlling the epidemic. Other interventions should be focused on social groups that are most at risk.

Only a few studies on the intranational distribution of behavioural risk factors have been published: a 2005 non-systematic review of surveys from 11 low-income and-middle-income country (LMIC) WHO subregions^11^ and a meta-analysis of studies examining tobacco use and income.^12^ These studies report a higher prevalence of tobacco use (odds ratio [OR] 1·48, 95% CI 1·38–1·59) and lower alcohol use in the poorest strata of LMICs than in more affluent strata. The most comprehensive analysis to date comes from an analysis of LMIC World Health Survey data from 2002–04.^13^ Self-reports from 232 056 participants from 48 countries (23 of which were LLMICs) suggested that those with more education and assets were more likely to be physically inactive and consume insufficient fruit and vegetables, and less likely to smoke daily, than were those with a lower level of education. The socioeconomic patterning of heavy episodic drinking was mixed and inequalities were more pronounced in the least developed countries. The findings from this study are now 10 years old and largely drawn from non-LLMICs. These reviews were limited by indirect estimates of behaviour and narrow definitions of socioeconomic status.

Non-communicable diseases are the leading cause of death and individuals living in LLMICs are 1·5 times more likely to die prematurely from these conditions than those living in high-income countries.^14^ With increasing international attention being paid to the epidemic, international and intranational health inequalities, and the potential role for development agencies in combatting non-communicable diseases, it is important that we have up to date information about the socioeconomic patterning of the most important non-communicable disease risk factors in lower-income settings.

We aimed to systematically review current evidence on the association between socioeconomic status and harmful use of alcohol, tobacco use, unhealthy diets, and physical inactivity within LLMICs.

## Methods

### Search strategy and selection criteria

We did a systematic review following a registered protocol and PRISMA guidelines^15^ (appendix). We searched Embase, MEDLINE, Web of Science, Global Health, and TRoPHI for all studies that included primary data published between Jan 1, 1990, and July 30, 2015. We also searched grey literature in Digital Dissertations (Global full-text plus), WHOLIS (WHO Library), and the WHO regional databases AIM (AFRO), LILACS (AMRO/PAHO), IMEMR (EMRO), IMSEAR (SEARO), and WPRIM (WPRO). We reviewed the first 30 results from Google Scholar and searched MEDLINE In-process and other non-indexed citations, the websites of the World Bank, DFID, USAID, and WHO, and scrutinised the reference lists of included papers and contacted key authors to uncover additional or forthcoming work.

We used English search terms (appendix) but did not restrict results by language or age of participants. Records were included if they presented primary data from one or more of the 84 LLMICs, as defined by the 2013 World Bank analytic classifications^16^ or on one or more non-communicable behavioural risk factor (defined by WHO as tobacco use, unhealthy diet, harmful alcohol use, and physical inactivity)^17^ and if the data were stratified by at least one socioeconomic indicator.

To accommodate differing views, capture all relevant studies, and broaden the systematic review, we included household or individual-level data measures of income, wealth, assets, socioeconomic status, education, caste, and occupation (where categories were ordinal). We excluded studies that did not allow comparison between more or less advantaged groups. Authors were contacted for additional data where socioeconomic status and behavioural risk factors were measured but reported independently. We used the same dates and strategy for all searches, tailored to specific databases by LA and an experienced medical librarian (NR).

With the use of a piloted form (appendix), LA and JW independently screened titles and abstracts, calculating percentage agreement and Cohen's κ statistic at 10% intervals (every 424 records). Once inter-rater agreement exceeded 95% and Cohen's κ was higher than 0·75 (excellent agreement^18^), LA screened all remaining records, bringing uncertainties to JW and KW, with disagreements resolved by group consensus. The same protocol was used for full-text systematic review. If data from included studies were unclear or if more information was required, the authors were contacted by email. If this information was not available, the study was excluded.

### Data analysis

LA used a piloted version of the Cochrane Effective Practice and Organisation of Care Group data collection checklist^19^ to extract relevant data from the included full-text studies including study type, methods, outcomes, and results (appendix). JW independently cross-checked a random 10% sample of included papers. Disagreements and ambiguities were resolved by full group consensus. Authors were contacted by email if more information was required.

We assessed data quality using a modified Newcastle-Ottawa scale,^20^ as recommended by the Cochrane Collaboration (appendix).^21^ Appropriate versions of the scoring rubric were used for randomised controlled trials, case-control studies, and cross-sectional studies. Scores were based on design-specific sources of bias, methods for selecting participants, exposure measures, outcome variables, and methods to control confounding. The source of funding was recorded for each study.

The main outcome was differences in prevalence or relative risk of non-communicable disease behavioural risk factors between different socioeconomic groups. We also planned to examine how age, sex, urban or rural location, and study quality affected findings. We assessed variability within studies in our quality scoring. This included considering the uniformity of training for those conducting the study and the instruments used to gather data. Significant heterogeneity between studies, particularly in the exposure and outcome measures precluded quantitative synthesis and meta-analysis. We used a narrative approach, grouping studies by outcome measure and WHO region. We analysed differences between outcomes, geographic regions, age groups, and sex. We also present sensitivity analyses for each risk factor having removed all medium and low-quality studies. The protocol of this study is registered with PROSPERO, number CRD42015026604. We used Excel to generate simple descriptive statistics.

### Role of the funding source

An employee of the funder (BM) contributed to the study design and review of draft manuscripts. The funder did not have any involvement in data collection, data analysis, or data interpretation. The corresponding author had full access to all the data in the study and had final responsibility for the decision to submit for publication.

## Results

**Figure 1:**
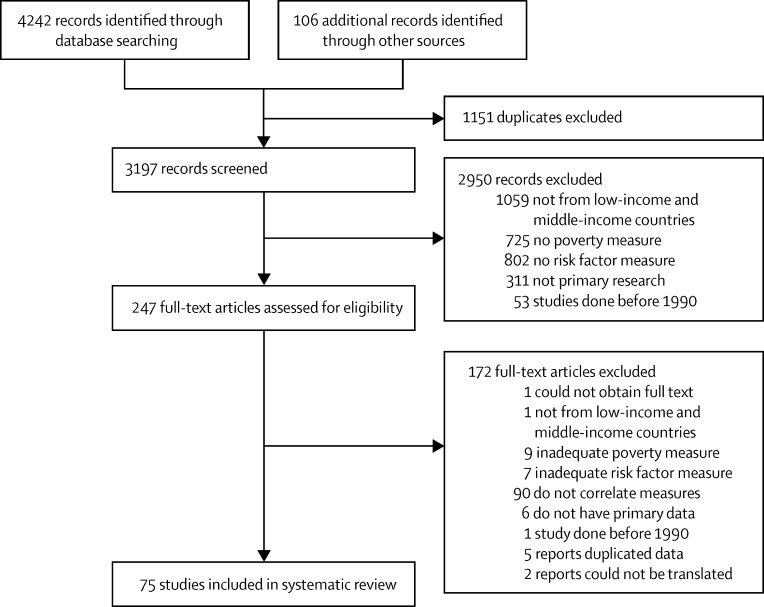
Study selection

Our literature search returned 4242 records and 106 additional records were retrieved from other sources (figure 1). Over 1000 studies were from higher-income or upper-middle-income countries. We assessed 247 full-text articles, of which 75 met our inclusion criteria. These articles covered 39 countries and presented data for 2 135 314 individuals aged older than 10 years. The median sample size (individuals for whom data of interest were reported) was 1984 (range 66–471 143).

Five articles presented data for all risk factors, 41 articles reported on a single risk factor, and the remaining 29 articles reported data for two or three risk factors. Ten different socioeconomic indicators were used (income, wealth or assets, state-defined poverty, literacy, education, occupational class, occupational status [employed or unemployed], job seniority, caste, researcher-defined socioeconomic status).

**Figure 2:**
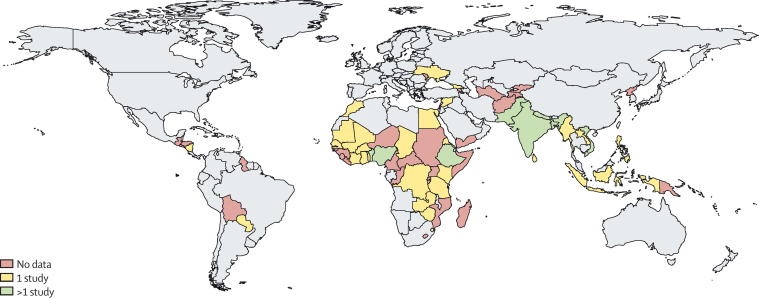
Sources of data from low-income and lower-middle-income countries

One article presented Global Adult Tobacco Survey data from two LLMICs,^22^ another reported World Health Survey data for smoking rates in 28 LLMICs,^23^ and the remaining 73 articles reported data from one country each. 44 studies were done in southeast Asian populations, with 35 pertaining to India. Data were presented for 20 African LLMICs countries, whereas the Americas and Europe had the lowest representation with two apiece. There were no data for 45 of the 84 LLMICs (figure 2).

Over half of the included studies had been published since 2010 and seven were published before 2000.^24^, ^25^, ^26^, ^27^, ^28^, ^29^, ^30^ 70 studies were cross-sectional, two were prospective longitudinal cohort studies,^31^, ^32^ two were case control studies,^33^, ^34^ and one study was a randomised controlled trial.^35^ Five studies were not peer reviewed; all were WHO STEPS surveys.^36^, ^37^, ^38^, ^39^, ^40^

Overall, 13 studies were of low quality, 33 were moderate, and 29 were of high quality, leading to a low risk of bias across studies. The most common study weaknesses were loss to follow-up and failure to control for confounding factors. Studies in African populations were more likely to be non-peer reviewed, to be of a lower quality, and have smaller sample sizes. Studies reporting data on Indian populations and tobacco use tended to be larger and of a higher quality than those for other populations and risk factors. Most studies were funded by governments, public health agencies, development agencies, and non-governmental or non-profit organisations. A summary of all high quality studies is presented in the table. Details of all included studies are available in the appendix.

**Table:** Characteristics of included high-quality studies

	**Site**	**Study design**	**Number of participants**	**Population**	**Age (years)**	**Exposure**	**Outcome**
**Physical activity**							
Kinra, 2010	India	Cross-sectional	1983	1600 villages in 18 states	20–69	Socioeconomic status	Low physical activity; <1·69 MET
Gupta, 2003	India	Cross-sectional	573	General population in Jaipur	NA	Education	Low physical activity; <30 min leisure time physical activity 3 times a week
Oanh, 2008	Vietnam	Cross-sectional	1776	STEPS survey in Ho Chi Minh	25–64	Assets/education/income	Low physical activity; <600 MET min per week
Gupta, 2012	India	Cross-sectional	6198	Middle-class areas of 11 cities	18–75	Education/occupation/socioeconomic status	Low physical activity; no regular work or leisure time physical activity
Dhungana, 2014	Nepal	Cross-sectional	406	Rural community in Sindhuli	20–50	Education/socioeconomic status/caste	Low physical activity; <150 minutes moderate physical activity/week
Zeba, 2014	Burkina Faso	Cross-sectional	330	Ouagadougou residents	25–60	Assets/education	Physical activity and sedentary time; means >3 h and <3 h MET, respectively
Reddy, 2007	India	Cross-sectional	19 969	Industrial workers from 10 cities	20–69	Education	Leisure time physical activity
Singh, 1997	India	Cross-sectional	1767	Two villages in rural north India	25–64	Socioeconomic status	Sedentary*
**Alcohol**							
Bonu, 2005	India	Cross-sectional	22 685	Inpatients from 1995 National Survey	>10	Alcohol use	Poverty; borrowing or financial distress during hospital admission
Gupta, 2012	India	Cross-sectional	6198	Middle-class areas of 11 cities	18–75	Education/occupation/socioeconomic status	Alcohol abuse
Samuel, 2012	India	Cross-sectional	2218	Rural and urban southern India	26–32	Assets/education	Alcohol use
Hashibe, 2003	India	Case-control	47 773	Adults in Kerala	>35	Income/education/occupation	Alcohol use
Neufeld, 2005	India	Cross-sectional	471 143	1995 National Sample Survey	>10	Poverty/caste/education	Alcohol use; regular use of any alcoholic beverage
Kinra, 2010	India	Cross-sectional	1983	1600 villages in 18 states	20–69	Socioeconomic status	Alcohol use; consumed >10 days per month over last 6 months
Dhungana, 2014	Nepal	Cross-sectional	406	Rural community in Sindhuli	20–50	Education/socioeconomic status/caste	Alcohol use; used alcohol up to 30 days before interview
Subramanian, 2005	India	Cross-sectional	301 984	1998 National Family Health Survey	>18	Assets/caste/education	Household member drinks alcohol
**Diet**							
Hashibe, 2003	India	Case-control	47 773	Adults in Kerala	>35	Income/education/occupation	Daily vegetables, high intake of fruit
Gupta, 2012	India	Cross-sectional	6198	Middle-class areas of 11 cities	18–75	Education/occupation/socioeconomic status	Less than two servings fruit and vegetables per day, more than 20 g fat per day
Ganesan, 2012	India	Cross-sectional	1261	Urban diabetics from Chennai	>40	Socioeconomic status	Low or high fibre diet; scored using a questionnaire
Kinra, 2010	India	Cross-sectional	1983	1600 villages in 18 states	20–69	Socioeconomic status	Low fruit and vegetable intake; <400 g/day
Dhungana, 2014	Nepal	Cross-sectional	406	Rural community in Sindhuli	20–50	Education/socioeconomic status/caste	Low fruit and vegetable intake; <400 g/day
Zeba, 2014	Burkina Faso	Cross-sectional	330	Ouagadougou residents	25–60	Assets/education	Unhealthy diet; fat/sugar/fibre/plant protein/complex carbohydrates
Agrawal, 2014a	India	Cross-sectional	156 317	National Family Health Survey	20–49	Caste/socioeconomic status	Non-vegetarian; eats meat, fish, milk, eggs, curd, dairy
Agrawal, 2014b	India	Cross-sectional	156 317	National Family Health Survey	20–49	Caste/wealth	Daily fish consumption
**Tobacco**							
Bonu, 2005	India	Cross-sectional	22 685	Inpatients from 1995 Nat. Survey	>10	Tobacco use	Poverty; borrowing or financial distress during hospitalisation
Hashibe, 2003	India	Case-control	47 773	Adults in Kerala	>35	Income/education/occupation	Smoking, tobacco chewing
Corsi, 2014	India	Cross-sectional	4534	20 villages in Andhra Pradesh	>20	Income/education	Current smoker, ever smoker
Kinra, 2010	India	Cross-sectional	1983	1600 villages in 18 states	20–69	Socioeconomic status	Daily smoker at any time in the last 6 months
Neufeld, 2005	India	Cross-sectional	471 143	1995 National Sample Survey	>10	Poverty/caste/education	Regular smoker, regularly chews tobacco
Gupta, 2003	India	Cross-sectional	573	General population in Jaipur	NA	Education	Past or present use of any tobacco product
Singh, 2000	India	Cross-sectional	1767	Two villages in rural north India	25–64	Socioeconomic status	Uses tobacco more than once per week
Gupta, 2012	India	Cross-sectional	6198	Middle-class areas of 11 cities	18–75	Education/occupation/socioeconomic status	Daily use of a tobacco product
Reddy, 2007	India	Cross-sectional	19 969	Industrial workers from 10 cities	20–69	Education	Use of any tobacco product in previous 30 days
Singh, 2007	India	Cross-sectional	2222	Residents of Moradabad	25–64	Socioeconomic status	Use of any tobacco product
Samuel, 2012	India	Cross-sectional	2218	Rural and urban southern India	26–32	Assets/education	Current tobacco user
Gupta, 2015	India	Cross-sectional	6198	Middle-class areas of 11 cities	>20	Education	Quit for >1 year having used tobacco for >1 year previously
Narayan, 1996	India	Cross-sectional	13 558	Residents of Delhi	25–64	Education/occupation	Current smoker or has smoked >100 times in the past
Rani, 2003	India	Cross-sectional	334 553	1998 National Family Health Survey	>15	Wealth/education/caste	Smokes, chews tobacco
Heck, 2012	Bangladesh	Cross-sectional	19 934	Married Bangladeshi adults	18–75	Education	Betel quid use
Dhungana, 2014	Nepal	Cross-sectional	406	Rural community in Sindhuli	20–50	Education/socioeconomic status/caste	Smoking until last 30 days before interview
Bovet, 2002	Tanzania	Cross-sectional	9254	Residents of Dar es Salaam	25–64	Wealth/education	Smokes one or more cigarettes per day
Minh, 2007	Vietnam	Cross-sectional	1984	2005 STEPS survey of Bavi district	25–64	Education/socioeconomic status	Smoker
Tonstad, 2013	Cambodia	Cross-sectional	5592	2006 National Tobacco Survey	>18	Education/income/occupation	Quit; not used tobacco for >2 years among ever users
Ali, 2006	Pakistan	Cross-sectional	411	Men from rural Sindh province	>18	Education/Income	Has smoked >100 cigarettes
Hosseinpoor, 2012	28 LLMICs	Cross-sectional	213 807	2003 World Health Survey	>18	Socioeconomic status	Daily or occasional tobacco smoker
Jena, 2012	India	Cross-sectional	69 296	2009 Global Tobacco Survey data	>15	Occupation/education	Hardcore smoker†
Kishore, 2013	India, Thailand, and Bangladesh	Cross-sectional	92 491	2009 Global Adult Tobacco Survey	>15	Education	Hardcore smoker†

MET=Metabolic Equivalent of Task. LLMIC=low-income and lower-middle-income countries.

* Walks less than 14·5 km, less than 20 flights of stairs, or does no moderate activity 5 days per week.

† Hardcore smoker is defined as someone who currently smokes daily, with no quit attempt in last 12 months or whose last quit was for less than 24 h; no intention to quit in next 12 months or not interested in quitting first smoke within 30 min of waking; and who has knowledge of harms. High-quality survey findings and findings for physical activity, alcohol, diet, and tobacco are in the appendix.

29 studies from 15 countries reported measures of physical activity.^25^, ^28^, ^31^, ^32^, ^36^, ^37^, ^38^, ^41^, ^42^, ^43^, ^44^, ^45^, ^46^, ^47^, ^48^, ^49^, ^50^, ^51^, ^52^, ^53^, ^54^, ^55^, ^56^, ^57^, ^58^, ^59^, ^60^ Three WHO STEPS surveys from India, Eritrea, and Côte d'Ivoire had not been peer-reviewed, but the remaining 26 were published in peer-reviewed journals. Three studies were low quality, 18 were moderate (including the WHO surveys), and eight were of high quality. Nine studies^37^, ^38^, ^48^, ^52^, ^53^, ^54^, ^55^, ^57^, ^61^ reported outcomes based on WHO recommendations and results from the International and Global Physical Activity Questionnaires,^62^, ^63^, ^64^ five studies used definitions derived from other sources,^32^, ^41^, ^44^, ^58^, ^60^ and 15 did not refer to any pre-existing definition.^25^, ^27^, ^31^, ^36^, ^42^, ^43^, ^45^, ^46^, ^47^, ^49^, ^50^, ^51^, ^56^, ^59^, ^65^ Measures of sedentary behaviour (not technically a non-communicable disease behavioural risk factor) were reported in 19 studies; high or sufficient levels of activity were reported in ten studies. All data were derived from survey instruments rather than the use of accelerometers or other devices.

There was a paucity of studies reporting adjusted results that were statistically significant; however, most studies found that individuals with a high socioeconomic status were less active than groups with a lower socioeconomic status, irrespective of outcome and exposure measures. This trend was consistent across studies from southeast Asia, the western Pacific, Africa, and the eastern Mediterranean. The notable exceptions were found in populations from urban areas: residents of Aleppo,^60^ pre-diabetics in southern India,^61^ and residents of multiple Indian cities.^43^, ^46^ In these settings, low-income and less educated groups had the highest prevalence of inactivity. Eight of the ten studies that stratified findings by sex found men to be more active than women;^25^, ^41^, ^45^, ^46^, ^50^, ^53^, ^54^, ^58^ the remaining two found female residents of Jaipur to be more active than men in all educational groupings;^42^ and no clear association was found in two rural north Indian villages.^44^

Most participants were aged 15–65 years old. Studies that excluded people aged over 60 years, or those younger than 30 years, still found that higher socioeconomic groups were the least active.^45^, ^47^, ^52^ The eight high-quality physical activity studies corroborate these findings. Single studies from the capital cities of Vietnam and Burkina Faso found that wealthy and educated individuals were the least active.^56^, ^57^ The six papers from India and Bangladesh showed that higher socioeconomic status was associated with lower levels of physical activity in rural settings,^41^, ^44^, ^52^ and this association was reversed in urban settings.^42^, ^43^ None of these studies controlled for occupation; however, Reddy and colleagues^46^ examined physical activity in 20 000 industrial workers and found that those with primary or no education were eight times less active than the most educated workers in their leisure-time (p<0·001). This study did not account for other important sources of physical activity including commuting, employment, or housework.

24 studies from ten countries reported measures of alcohol use.^25^, ^27^, ^28^, ^31^, ^32^, ^34^, ^35^, ^36^, ^38^, ^39^, ^40^, ^41^, ^43^, ^45^, ^47^, ^52^, ^66^, ^67^, ^68^, ^69^, ^70^, ^71^, ^72^, ^73^ Three studies were graded as low quality (including the only randomised controlled trial), 13 were moderate, and eight were of high quality, including the only case-control study. Four studies reported prevalence of harmful alcohol use, defined in terms of the frequency and volume of alcohol consumed.^35^, ^40^, ^43^, ^69^ The remaining 18 reported measures of any alcohol use as the outcome variable. There was reasonable agreement between the various socioeconomic proxies; none of the studies that used multiple exposure measures found conflicting assessments.

One study found that alcohol users were more likely to experience impoverishment than non-users but this association was not statistically significant.^66^ Overall, low-income uneducated groups in rural areas were the most likely to engage in harmful drinking behaviour. The widest differences were observed between different educational groups; smaller gaps were observed when comparing income strata.

Analysing the findings by region, alcohol use—while not necessarily at harmful levels—was most prevalent in low-income and less-educated groups across India^32^, ^34^, ^38^, ^41^, ^45^, ^47^, ^67^, ^68^, ^70^, ^71^ and in the solitary study from the Americas.^73^ Prevalence of alcohol use tended to be higher in more affluent and well educated Africans;^25^, ^27^, ^28^, ^31^, ^35^ however, most of these studies were published in the 1990s^25^, ^27^, ^28^ and sample sizes were in the hundreds for all but one study—a randomised controlled trial graded as low quality.^35^ No studies from Europe, the eastern Mediterranean, or western Pacific regions were published. All five studies that reported results by sex found men to drink more than women;^25^, ^27^, ^41^, ^45^, ^70^ however, inequalities were often more pronounced between women. The smaller numbers of women in these studies rendered many of these findings not significant.

Six of the eight high-quality studies assessed alcohol use rather than harmful use of alcohol. With the exception of a 2012 survey of younger Indian adults,^67^ these studies all found that lower socioeconomic groups were the most likely to use alcohol.^34^, ^41^, ^52^, ^70^, ^71^ The largest differences were observed between members of different castes and educational groups. Gupta and colleagues^43^ assessed (undefined) alcohol abuse in middle-class urban Indians, finding minor differences between educational and self-assessed socioeconomic tertiles. Individuals in middle occupational classes had double the rate of alcohol abuse compared with the lowest occupational class (10·8% *vs* 5·1%); however, no measures of significance were presented.^43^

26 papers from 11 countries reported on eight different aspects of diet.^24^, ^25^, ^29^, ^30^, ^34^, ^36^, ^38^, ^40^, ^41^, ^43^, ^45^, ^50^, ^51^, ^52^, ^56^, ^60^, ^65^, ^68^, ^72^, ^74^, ^75^, ^76^, ^77^, ^78^, ^79^, ^80^ There was one case-control study^34^ and the remainder were cross-sectional. Four studies were low quality, 14 were moderate, and eight were of high quality. Six studies, from Pakistan,^24^ India,^29^, ^30^, ^43^, ^65^ and Nigeria^25^ found higher consumption of unhealthy fats in individuals of high socioeconomic status. Two studies examining salt intake found a higher prevalence in high-income households in Chennai^77^ and non-significant differences in a low quality multisite Indian survey.^65^ Two higher quality African studies found that the individuals of high socioeconomic status were more likely to consume diets high in processed foods.^56^, ^78^

Studies from Indonesia, Syria, Nepal, Benin, Eritrea, and Nigeria all found lower fruit and vegetable intake in less affluent and less educated groups.^36^, ^50^, ^52^, ^60^, ^72^, ^79^, ^80^, ^81^ These studies tended to present results that were either significant but unadjusted or adjusted but not significant; only two studies presented significant adjusted findings.^80^, ^81^ Six larger and higher quality studies from India predominantly found lower fruit and vegetable intake in groups of lower socioeconomic status.^34^, ^38^, ^41^, ^45^, ^50^, ^65^ One high-quality survey of low fibre intake found a low socioeconomic status preponderance^76^ and three large Indian studies found less affluent groups to consume the least fish and most meat.^68^, ^74^, ^75^ There was good agreement between different poverty markers; all but two studies that used more than one measure found that similar groups were identified as consuming the least healthy diet.^43^, ^52^ Women were found to consume less fruit and vegetables than were men in two Indian studies.^41^, ^45^ Four other studies reporting dietary findings by sex found non-significant differences.^24^, ^25^, ^29^, ^50^ In 320 elderly residents of Baroda city, India, men consumed twice as much fat as women.^30^

When we examined the effect of age on these dietary findings, we found that studies examining older populations came to the same conclusions as other studies examining the same dietary component.^30^, ^34^, ^41^, ^76^, ^79^, ^80^ One cross-sectional study examining cholesterol intake in Pakistani schoolchildren found the highest consumption in boys and girls from the highest socioeconomic group.^24^ After removing all medium and low-quality studies, five high-quality studies suggest that high socioeconomic groups consume more fat, fish, fibre, and fruit and vegetables than lower socioeconomic groups in southeast Asia.^34^, ^41^, ^43^, ^52^, ^74^, ^75^ The remaining small cross-sectional study in Burkina Faso^56^ found that those with the most education and assets were twice as likely to consume an unhealthy diet (high in fat and sugar, low in fibre, plant protein, and complex carbohydrates) as those with the lowest education and assets.

50 studies reported data for tobacco use in 39 countries (appendix), almost twice the number of studies examining other risk factors.^22^, ^23^, ^26^, ^27^, ^31^, ^32^, ^33^, ^34^, ^37^, ^38^, ^39^, ^41^, ^42^, ^43^, ^44^, ^45^, ^46^, ^47^, ^49^, ^50^, ^51^, ^52^, ^58^, ^59^, ^60^, ^65^, ^66^, ^67^, ^68^, ^71^, ^73^, ^82^, ^83^, ^84^, ^85^, ^86^, ^87^, ^88^, ^89^, ^90^, ^91^, ^92^, ^93^, ^94^, ^95^, ^96^, ^97^, ^98^, ^99^, ^100^ Eight studies were low quality, 18 were moderate, and 23 were of high quality—a much higher proportion than for other risk factors. 33 studies reported smoking as an outcome variable using a range of definitions,^23^, ^26^, ^27^, ^31^, ^32^, ^37^, ^38^, ^39^, ^41^, ^42^, ^45^, ^50^, ^51^, ^52^, ^60^, ^68^, ^73^, ^83^, ^91^, ^93^, ^94^, ^95^, ^96^, ^97^, ^99^, ^100^ two reported hardcore smoking (very low chances of ever quitting),^22^, ^88^, ^101^ two reported quitting,^87^, ^98^ and one study each was included on use of manipuri,^33^ betel quid,^90^ and the likelihood of smokers experiencing financial distress.^66^

The popularity of chewing versus smoking tobacco varied by setting, but women were more likely to chew tobacco than smoke.^84^, ^89^ Although levels of smoking and chewing were broadly commensurate within populations, socioeconomic inequalities were more pronounced with smoking. Jena and colleagues^88^ found an adjusted but non-significant higher prevalence of hardcore smoking in well educated individuals in a large nationally representative Indian sample.^88^ Illiterate individuals and those who were poorly educated were more likely to smoke manipuri^33^ and betel quid,^90^ and less likely to quit all forms of tobacco.^87^, ^98^

One moderate quality survey of 233 young Keralan men found a significantly higher prevalence of tobacco use in middle-class students, adjusting for age and other confounders.^86^ The remaining 49 studies found tobacco use, in all forms, to be more prevalent in low socioeconomic groups than in high socioeconomic groups, including 17 studies presenting statistically significant adjusted results from 18 different countries.^23^, ^26^, ^41^, ^42^, ^43^, ^46^, ^47^, ^58^, ^67^, ^71^, ^82^, ^83^, ^89^, ^94^, ^97^, ^98^, ^99^

The finding that low socioeconomic groups were more likely to use tobacco than high socioeconomic groups was the same in every geographical region. Studies that examined older populations came to the same conclusions.^34^, ^47^, ^92^ Two studies examined tobacco use in young adults, both from southern India; Lal and Nair's subanalysis^86^ of Keralan data from the Global Adult Tobacco Survey, which has been previously mentioned, uniquely found higher tobacco usage in 233 highly educated and middle socioeconomic status men aged 15–24 years. Samuel and colleagues found the highest usage in the poorest and least educated 26–32 year olds in a cross sectional survey of 2218 26–32 year olds living in south India.^67^

Differences between educational groups were larger than differences between castes and income or wealth strata.^26^, ^43^, ^45^, ^71^ Neufeld and colleagues found that measures of caste and state-defined poverty were associated with wider inequalities for chewing tobacco than cigarette use.^71^ All 15 studies that stratified prevalence by sex found men to smoke more than women, often by a large margin.^23^, ^26^, ^27^, ^41^, ^42^, ^44^, ^45^, ^46^, ^50^, ^58^, ^83^, ^84^, ^85^, ^89^, ^96^ Removing all low and moderate quality studies did not change the findings. Among the 24 high-quality studies, education remained the strongest predictor of betel quid and tobacco use. Those with no formal education were between 1·75 and 6·50 times more likely to smoke than those with at least a secondary education.^26^, ^34^, ^42^, ^46^, ^67^, ^83^ Low income, caste, and socioeconomic status were associated with a tobacco use prevalence roughly twice that of high-status groups.

**Figure 3:**
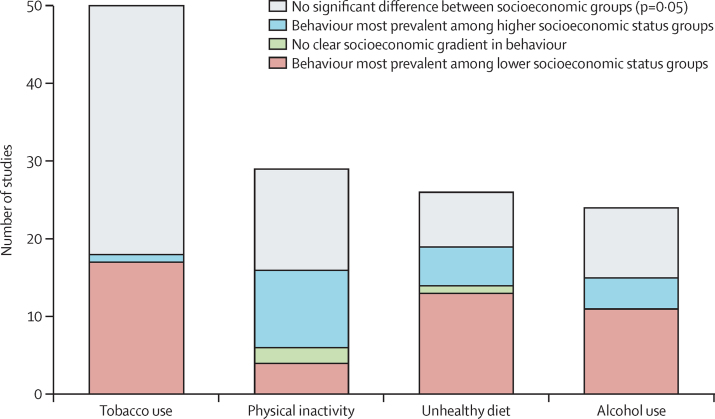
Number of studies for each risk factor showing the socioeconomic group with the highest risk

Overall, low socioeconomic groups in most of the LLMICs in which evidence was available were more likely to use tobacco and alcohol, and to consume less fruit, vegetables, fish, and fibre, and more meat than high socioeconomic groups. High socioeconomic status groups tended to have higher levels of physical inactivity and consume more fats, salt, and processed foods than low socioeconomic groups (figure 3). While the included studies presented clear patterns for tobacco use and physical activity, heterogeneity between dietary outcome measures and a paucity of evidence around harmful alcohol use limit the certainty of these findings.

## Discussion

This systematic review identifies broad trends in global behavioural risk factors for non-communicable diseases, finding that low socioeconomic groups in many countries are more likely to drink alcohol, use tobacco, and consume insufficient fruit and vegetables than are high socioeconomic groups. High socioeconomic groups tend to be more inactive and consume more fats, salt, and processed food.

This systematic review is the first to examine the socioeconomic distribution of all four major behavioural risk factors within LLMICs. Our findings substantially augment the scant evidence from previous LLMIC-based reviews on individual risk factors; Ciapponi and colleagues^12^ showed a significant income gradient for tobacco use but their focus on income only excluded many of the studies included in our systematic review. With the use of a broad range of socioeconomic indicators, we found significant differences between castes, classes, sexes, and occupational groups with the widest differences observed across educational strata.

Our tobacco findings mirror the well established inequalities from high-income countries, where low-income groups are the most likely to smoke, start smoking earlier, consume more tobacco, quit less successfully, experience more adverse health effects, and die at a younger age than affluent groups.^23^, ^102^, ^103^, ^104^, ^105^ The last study to examine socioeconomic status and tobacco in LMICs was performed by Blakely and colleagues in 2005.^11^ They found that low-income groups from 11 LMIC WHO subregions had a marginally higher prevalence of tobacco use and lower use of alcohol than did higher-income groups.^11^ Most studies included in our systematic review used direct surveys, whereas Blakely and colleagues relied on estimates of consumption derived from household economic data.^11^ Evidence summarised in the Global Alcohol Report also suggests that abstinence is more common in low-income groups and that alcohol-related harm is more prevalent in low socioeconomic groups than in high-income groups; however, these data are mainly drawn from high-income countries.^106^, ^107^ Our findings suggest that alcohol use and harmful alcohol use tend to be most prevalent in low socioeconomic groups. We note that data for harmful alcohol use were lacking from 79 of 84 LLMICs and the few existing African studies are of low quality. There is an urgent need to quantify the burden of risky alcohol use in LLMICS.

Our dietary findings complement studies from high-income countries that have consistently found a positive association between socioeconomic status and consumption of fruit, vegetables, fibre, and fish.^108^, ^109^, ^110^, ^111^, ^112^, ^113^, ^114^ Whereas low socioeconomic status groups in high-income settings tend to consume higher levels of salt and processed food,^111^, ^115^ we found the opposite in LLMICs, but there was a conspicuous absence of studies on salt intake given the impact of this dietary risk factor.^1^ Our findings corroborate those of Mayén and colleagues^116^ who found higher consumption of all foods except fibre in high socioeconomic status groups in their systematic review of dietary patterns in LMICs; however, three quarters of their included studies were from upper-middle-income countries.

Our finding that rural high socioeconomic status groups tend to be the most physically inactive departs from the experience of high-income countries.^117^, ^118^, ^119^, ^120^ A possible explanation is that rural low socioeconomic status groups tend to work in physically demanding occupations in LLMICs.^121^ In cities, this association was reversed and evidence from China suggests that low socioeconomic status migrants take up less physically demanding jobs when they move to cities.^122^ If cities truly attenuate the socioeconomic gradient in occupational activity, then leisure-time physical activity might be proportionally more important as an explanatory variable. Reddy and colleagues found that higher socioeconomic status Indian groups engaged in more leisure-time physical activity than low socioeconomic status groups in urban areas.^46^ A large systematic review from mainly high-income countries has shown that leisure activity is associated with larger health gains than occupational activity.^123^ Our findings highlight the need for more research in LLMICs to explore the health effects of various domains of physical activity on different socioeconomic groups in rural and urban settings.

This systematic review was done in line with PRISMA and Cochrane guidance, following a registered protocol and assessing risk of bias using well established criteria. The bidirectional association between socioeconomic status and health is widely averred but infrequently assessed within LLMICs.^11^, ^124^, ^125^, ^126^, ^127^, ^128^ To our knowledge, this is the first systematic review to explore intranational socioeconomic patterning of behavioural risk factors in these countries and the first study to report that increasing wealth and education are associated with physical inactivity and increasing consumption of fats, salt, and processed food in a number of LLMICs. Our work demonstrates important associations and emphasises the importance of context; trends vary by region, sex, urbanicity, and exposure. Most included studies were moderate to high quality and almost invariably used a cross-sectional, survey-based approach.

Our method was designed to capture all studies on socioeconomic status and non-communicable disease behavioural risk factors. As a result, our findings are extremely heterogeneous and require careful interpretation. We treated the highest and lowest groupings of each exposure (eg, education, income, or social class) as if they were interchangeable even though each study tended to use a unique definition, cutoff, and study population. This allowed us to identify broad trends for future research to examine in detail; however, it meant that our findings should not be seen as definitive. The large amount of data also prevented us from presenting deep analysis of each risk factor in this systematic review; however, our comprehensive data extraction and presentation of all original data and subgroup descriptors in the appendix allows further study to build upon this initial global assessment. The heterogeneity in outcome measures for each risk factor limits the ability of any systematic review to synthesise findings cleanly, and the surfeit of smoking and alcohol definitions is especially noteworthy given the relative homogeneity of the products. A further source of bias was a dependence on survey instruments rather than objective measurements to establish tobacco use, alcohol use, diet, and physical activity between the studies. Survey responses are not very reliable and socioeconomic differences in recall bias might affect observed gradients in behaviour.

Use of the 2013 World Bank classification of income excluded countries that have only recently been reclassified as upper-middle-income; however, our focus on countries that are currently low-income enhances the usefulness of this systematic review as development agencies are moving away from upper-middle-income settings.^129^ Because of resource constraints, we were unable to perform a duplicate screen and data extraction for every record. Our high levels of agreement at each stage, including perfect agreement in triple-checked data extraction samples, provide reassurance that this systematic review includes all relevant data.

In view of the broad scope of this systematic review, the fact that over half of the countries classed as low-income or lower-middle-income were not represented in our search results is striking. Almost half of the included studies relate to India, and the evidence from the Americas, the eastern Mediterranean, and Europe is relatively scant. The fact that so many LLMICs were not represented is a major finding, but also a weakness in itself; the excellent evidence from India is not generalisable to all low socioeconomic groups in LLMICs and research is needed to explore whether the patterns we identify hold true in countries where surveillance is non-existent.

Of the 47 publicly available LLMIC-based WHO STEPS surveys,^130^ only five present behavioural risk factors stratified by any marker of socioeconomic status.^35^, ^36^, ^37^, ^38^, ^39^ All STEPS reports should make these routinely collected data publicly available.

Our findings provide an overview of the current evidence, underlining intranational trends and data gaps. Policy makers and national development agencies working in the countries where 82% of premature deaths occur should review the evidence relevant to their setting and consider whether their current non-communicable disease prevention strategies are appropriate. Where low socioeconomic status correlates with non-communicable disease risk factors, governments can use development funds to simultaneously improve literacy, living standards, and income alongside health. The definitions used to identify behavioural risk factors are inconsistent, and data are not available for most LLMICs. Rectification of these issues, with surveillance reporting risk factors stratified by socioeconomic status, is an obvious research priority. Nevertheless, this should not delay action in the countries where data exist.
