# Supplementary material for: Socioeconomic status and non-communicable disease behavioural risk factors in low-income and lower-middle-income countries: a systematic review
Source: Lancet Glob Health. 2017 Feb 11;5(3):e277–89. doi: 10.1016/S2214-109X(17)30058-X (PMC5673683; doi:10.1016/S2214-109X(17)30058-X)
Supplement: Supplementary appendix [file mmc1.pdf]

THE LANCET
Global Health

Supplementary appendix

This appendix formed part of the original submission and has been peer reviewed. We post it as supplied by the authors.

Supplement to: Allen L, Williams J, Townsend N, et al. Socioeconomic status and non-communicable disease behavioural risk factors in low-income and lower-middle-income countries: a systematic review. *Lancet Glob Health* 2017; **5:** e277–89.

Appendix

Tables

High quality studies	Page 2
Alcohol	Page 38
Physical activity	Page 49
Diet	Page 65
Tobacco	Page 79

Forms

Search terms	Page 110
Screening form	Page 113
Quality scoring rubric	Page 114
PRISMA checklist	Page 115
Data extraction checklist	Page 117

Design, n	Sample	Exposure	Outcome	n	Exposure subgroup	Value	95% CI	p
Physical activity								
**Kinra et al. (2010)** - Funded by the Wellcome Trust								
cross-sectional 1,983 India	Nationally representative sample of rural inhabitants from 1600 villages in 18 states. Aged 20-69 years	**SES (men) assets, housing**	**Low physical activity** <1.69 MET	147	Low (asset score)	**65.2%**	57.5-72	0.11
				358	Middle (asset score)	**72.4%**	67.8-77.0	
				870	High (asset score)	**72.9%**	70.0 -75.9	
		**SES (women) assets, housing**	**Low physical activity**	106	Low (asset score)	**66.0%**	54.5-77	0.084
				143	Middle (asset score)	**73.5%**	66.0-81.0	
adjusted for age				359	High (asset score)	**76.5%**	72-81	
**Gupta et al. (2003)** - Source of funding not reported								
cross-sectional 573 India	Serial cross-sectional surveys from the general population of Jaipur. Data taken from the most recent round. Age not reported	**Education (men)**	**Physical Inactivity** Leisure time physical activity <30 minutes 3x week	103	Low (no formal education)	**89.3%**		0.016
				182	Middle-low (1-10 years)	**64.3%**		
				202	Middle-high (11-15 years)	**50.5%**		
				63	High (>16 years)	**42.9%**		
		**Education (women)**	**Physical Inactivity**	213	Low (no formal education)	**89.2%**		0.038
				163	Middle-low (1-10 years)	**58.9%**		
				161	Middle-high (11-15 years)	**39.8%**		
adjusted for age				36	High (>16 years)	**33.3%**		

Singh et al. (1997) - Source of funding not reported

cross-sectional 1,767 India	Residents of two villages in rural north India. Aged 25-64 years	**SES (men) education, occupation, income, assets, housing**	**Sedentary lifestyle** walk <14.5km/week, climb <20flights stairs or no moderate activity 5 days/week	147	High	**44.20%**		<0.05
				147	Middle-high	**34.60%**		
				287	Middle-low	**3.50%**		
				313	Low	**not reported**		
		**SES (women) education, occupation, income, assets, housing**	**Sedentary lifestyle**	115	High	**13%**		<0.01
				112	Middle-high	**58%**		
				313	Middle-low	**20.40%**		
no adjustment described				335	Low	**4.40%**		

<u>Gupta et al. (2012)</u> - Funded by the South Asian Society of Atherosclerosis and Thrombosis

ross-sectiona 6,198 India	General population in middle-class areas of 11 cities, excluded house-bound, pregnant and those likely to die within 6 months. Aged 18-75 years	**Education**	**Low physical activity** no regular work-related or leisure time physical activity	1,248	Low (0-10 years)	**37.2%**		
				2,956	Middle (11-15 years)	**45.5%**		
				1,366	High (>15 years)	**35.5%**		
		**Occupational Class (British Social Register, housewife = husband)**	**Low physical activity**	1,287	Low (4-5)	**47.4%**		
				1,677	Middle (3 manual/non-manual)	**43.7%**		
				3,018	High (1-2)	**39.1%**		
		**SES (self-assessed)**	**Low physical activity**	374	Low (score 1-3)	**42.3%**		
				3,622	Middle (score 4-6)	**40.1%**		
adjusted for age and sex				1,114	High (score 7-10)	**36.9%**		

<u>Reddy et al. (2007)</u> - Funded by the Indian Ministry of Health; WHO

cross-sectional 19,969	Industrial workers and their relatives from ten urban sites across India. Aged 20-69 years.	**Education (men)**	**Physical activity** leisure time physical activity	1,611	High (postgraduate)	**1**	OR	<0.001
				2,607	Middle-high (secondary-tertiary)	**0.9**	0.8-1.1	

				5,820	Middle-low (primary-secondary)	**0.7**	0.6-0.8	
				1,859	Low (none-primary)	**0.2**	).18-0.25	
		**Education (women)**	**Physical activity**	1,611	High (postgraduate)	**1**	OR	<0.001
				2,607	Middle-high (secondary-tertiary)	**0.9**	0.7-1.1	
				5,820	Middle-low (primary-secondary)	**0.6**	0.5-0.8	
adjusted for age and occupation				1,859	Low (none-primary)	**0.3**	0.2-0.4	

Dhungana et al. (2014) - Source of funding not reported

cross-sectional 406 Nepal	Rural community in the Sindhuli district. Aged 20-50 years	**Caste**	**Insufficient physical activity** <150 minutes moderate physical activity/week	High (Brahman/Chhetri)	**47.5%**		0.06
				Middle (Adhibasi/Janajati)	**51.6%**		
				Low (Dalits)	**28.6%**		
		**Education**	**Insufficient physical activity**	Low (No formal education)	**45.7%**		0.0286
				Middle-low (lower than primary)	**37.9%**		
				Middle-high (primary)	**50.0%**		
				High (Secondary and higher)	**61.4%**		
		**SES (education, occupation, income)**	**Insufficient physical activity**	Lowest	**54.1%**		0.8
				Middle-low	**42.6%**		
				Middle-high	**50.0%**		
no adjustment described				Highest	**62.5%**		

Zeba et al. (2014) - Funded by the Canadian International Development Agency

cross-sectional 330 Burkina Faso	Burkinabe born and resident in Ouagadougou for >6 months. Excludes pregnant or lactating women and physically and mentally disabled	**Asset score**	**Physical activity** mean hours of physical activity equivalent to >3 MET	Low (tertile score)	**5.3h**	(SD) 2.7	<0.001
				Medium (tertile score)	**4.3h**	(SD) 2.2	
				High (tertile score)	**3.4h**	(SD) 1.6	

	individuals.						
	Aged 25-60 years	**Asset score**	**Sedentary time** mean hours of sedentary time equivalent to <3 MET hours	Low (tertile score)	**10.7h**	(SD) 3.[illegible]	<0.001
				Medium (tertile score)	**11.4h**	(SD) 3.1	
				High (tertile score)	**12.4h**	(SD) 2.8	
		**Education**	**Physical activity**	Low (no formal)	**4.8h**	(SD) 2.4	0.002
				Medium (elementary)	**4.6h**	(SD) 2.5	
				High (High school and above)	**3.8h**	(SD) 2.1	
		**Education**	**Sedentary time**	Low (no formal)	**11.1h**	(SD) 3.2	0.251
				Medium (elementary)	**11.6h**	(SD) 3.1	
no adjustment described				High (High school and above)	**11.7h**	(SD) 3.0	

<u>Oanh et al. (2008)</u> - Funded by Atlantic Philanthropies

[illegible]ross-section[illegible]	Representative STEPS	**Education**	**Insufficient physical activity**	less than primary	**1**	OR, p for trend <0.001	
1,776	survey from Ho Chi Minh		<600 MET mins/week	primary completed	**0.88**	0.56-1.37	
Vietnam	city.			secondary completed	**0.93**	0.55-1.56	
	Aged 25-64 years			high school completed	**1.09**	0.68-1.75	
				some college	**1.32**	0.63-2.76	
		**Income**	**Insufficient physical activity**	VND <1,000,000/month	**1**	OR, p for trend <0.001	
				VND 1,000,000-3,000,000	**1.42**	1.02-2	<0.05
				VND 3,000,000-5,000,000	**1.51**	0.94-2.43	
				VND >5,000,000	**1.77**	1.05-2.[illegible]	<0.05
		**Wealth (assets)**	**Insufficient physical activity**	Lowest	**1**	OR, p for trend <0.001	
				Second	**1.29**	0.9-1.84	
				Middle	**1.67**	[illegible].26-2.2[illegible]	<0.05
				Fourth	**1.87**	[illegible].15-3.0[illegible]	<0.05
				Highest	**1.86**	[illegible].29-2.6[illegible]	<0.05

no adjustment described			*variables; age group, sex, ethnicity, education, occupation, household SES, tobacco use, alcohol consumption*					

Alcohol

Bonu et al. (2005) - Source of funding not reported

cross-sectional 22,685 India	Hospitalised participants of the 1995/6 Indian National Sample Survey. Aged >10 years	**Current regular alcohol use**	**Poverty** borrowing/financial distress during hospitalisation		Non-user	**1**	OR	
					Alcohol user	**1.1**	0.64-1.8	>0.05
adjusted for: head of household, age, sex, level of schooling, marital status, state. number of days of hospitalisation used to control for severity of illness.								

Gupta et al. (2012) - Funded by the South Asian Society of Atherosclerosis and Thrombosis

cross-sectional 6,198 India	General population in middle-class areas of 11 cities, excluded house-bound, pregnant and those likely to die within 6 months. Aged 18-75 years	**Education**	**Alcohol abuse**	1,248	Low (0-10 years)	**9.6%**		
				2,956	Middle (11-15 years)	**10.3%**		
				1,366	High (>15 years)	**11.5%**		
		**Occupational Class** (British Social Register, housewife = husband)	**Alcohol abuse**	1,287	Low (4-5)	**5.1%**		
				1,677	Middle (3 manual/non-manual)	**13.3%**		
				3,018	High (1-2)	**10.8%**		
		**SES (self-assessed)**	**Alcohol abuse**	374	Low (score 1-3)	**11.2%**		
				3,622	Middle (score 4-6)	**11.6%**		
adjusted for; age and sex				1,114	High (score 7-10)	**11.4%**		

Samuel et al. (2012) - Funded by the British Heart Foundation

cross-sectional 2,218 India	Young adults from population-based birth cohort in rural and urban areas of southern India. Aged 26-32 years	**Wealth (asset score)**	**Alcohol use**		Low (quintile 1)	**1**	OR	
					Middle-low (quintile 2)	**0.8**	0.6-1.3	

					Middle (quintile 3)	**0.8**	0.6-1.3	
					Middle-high (quintile l4)	**1**	0.7-1.5	
					High (Quintile 5)	**0.8**	0.5-1.3	
		**Education**	**Alcohol use**		Low (0 years formal schooling)	**1**	OR	
					Middle-low (1-8 years)	**1.3**	0.7-2.3	
					Middle-high (9-12)	**1.1**	0.6-1.9	
					High (>12 years)	**0.6**	0.3-1.2	
adjusted for; gender, place of residence, possessions score, adult educational status, paternal educational status								

Kinra et al. (2010) - Funded by the Wellcome Trust

cross-sectional 1,983	Nationally representative sample of rural inhabitants from 1600 villages in 18 states.	**SES (men) assets, housing**	**Alcohol use**	147	Low (asset score)	**33.7%**	26.2-41	<0.001
India	Aged 20-69 years		consumed >10 days/month any time in previous 6 months	358	Middle (asset score)	**26.9%**	22.3-31.5	
				870	High (asset score)	**20.2%**	17.5-22.9	
		**SES (women) assets, housing**	**Alcohol use**	106	Low (asset score)	**11.2%**	5.6-16.[illegible]	<0.001
				143	Middle (asset score)	**8.1%**	3.4-12.9	
adjusted for; gender, place of residence, possessions score, adult educational status, paternal education				359	High (asset score)	**2.5%**	1.0-4.1	

Subramanian et al. (2005) - Source of funding not reported

cross-sectio	Data from the nationally	**Caste (men)**	**Drink alcohol**	60,001	Other (High)	**1**	OR	
301,984	representative 1998/9		household member drinks alcohol	41,984	Other backward class (Medium)	**1.08**	1.04-1.12	
India	Indian National Family			24,503	Scheduled caste (Low)	**1.43**	1.37-1.49	
	Health Survey.			18,362	Scheduled tribe (Low)	**2.04**	1.92-2.17	
	Aged >18 years							
		**Education (men)**	**Drink alcohol**	4,328	Highest (postgraduate)	**1**	OR	
				13,004	High (college)	**1.11**	1.01-1.22	
				14,170	Medium-high (higher secondary)	**1.21**	1.1-1.33	
				54,516	Medium-low (secondary)	**1.75**	1.61-1.91	

				27,504	Low (primary)	**2.13**	1.95-2.33
				38,523	Lowest (Illiterate)	**2.28**	2.08-2.5
		**Wealth (men) assets**	**Drink alcohol**	34,133	Highest	**1**	OR
				31,611	High	**1.12**	1.07-1.18
				30,413	Medium	**1.30**	1.24-1.37
				28,985	Low	**1.59**	1.51-1.68
				26,903	Lowest	**1.92**	1.81-2.03
		**Caste (women)**	**Drink alcohol**	58,977	Other (High)	**1**	OR
				41,614	Other backward class (Medium)	**1.55**	1.31-1.83
				23,847	Scheduled caste (Low)	**1.66**	1.47-1.87
				18,373	Scheduled tribe (Low)	**3.74**	2.79-5
		**Education (women)**	**Drink alcohol**	2,154	Highest (postgraduate)	**1**	OR
				6,948	High (college)	**0.85**	0.72-1
				7,571	Medium-high (higher secondary)	**0.55**	0.41-0.75
				33,133	Medium-low (secondary)	**0.81**	0.62-1.05
				22,952	Low (primary)	**1.00**	0.74-1.35
				77,181	Lowest (Illiterate)	**1.31**	1-1.71
		**Wealth (women) assets**	**Drink alcohol**	34,049	Highest	**1**	OR
				30,798	High	**1.17**	0.94-1.45
				29,156	Medium	**1.60**	1.23-2.09
				27,836	Low	**1.96**	1.53-2.43
				28,100	Lowest	**2.72**	2.18-3.39

adjusted for; living environment, marital status, age, religion, caste, education, household standard of living index

Hashibe et al. (2003) - Funded by Assoc. Int Cancer Research; Imperial Cancer Research Fund; National Cancer Institute (USA) & UCLA Jonnson Cancer Centre Foundation

case-control 47,773 India	Study examining SES and premalignant oral lesions in Kerala. Data is presented	**Income**	**Drinking**		Low (INR<1500	**16.8%**	
					Middle-low (INR 1500-3000)	**12.4%**	
					Middle-high (INR 3001-5000)	**11.2%**	

	linking NCD risk factors with SES markers for the 47,773 controls only. Aged >35 years				High (INR>5000)	**9.3%**		<0.0001
		**Education**	**Drinking**		None/illiterate	**11.6%**		
					None/literate	**8.3%**		
					Primary	**15.4%**		
					Middle	**17.2%**		
					>High school	**15.4%**		<0.0001
		**Occupation**	**Drinking**		Manual	**12.5%**		
					Teacher/office	**18.7%**		
					Business	**30.0%**		
					Retired	**24.7%**		
no adjustment described					Other	**34.3%**		<0.0001

Neufeld et al. (2005) - No external funding

cross-sectio 471,143 India	Data from the 1995/6 Indian National Sample Survey. Aged >10 years	**Poverty***	**Alcohol** regular use of any alcoholic beverage	303416	High (above poverty line)	**1**	OR	
				167,727	Low (below poverty line)	**1.2**	1.1-1.4	
		**Caste****	**Alcohol**	334,512	High	**1**	OR	
				136,631	Low	**3.4**	3-3.8	
		**Education**	**Alcohol**	273,069	High (formal education)	**1**	OR	
				188,956	Low (no formal education)	**1.3**	1.2-1.4	

**Planning commission of India definition - income required to ensure adequate intake of calories (INR 2,100 urban; INR 2,400 rural)*

***Scheduled Castes and tribes - identified in the Indian Constitution as especially disadvantaged or needy*

adjusted for; age group, gender caste, income, residence, education

Dhungana et al. (2014) - Source of funding not reported

cross-sectional 406	Rural community in the Sindhuli district.	**Caste**	**Alcohol use**		High (Brahman/Chhetri)	**31.1%**		<0.001

Nepal	Aged 20-50 years		use of alcohol until up to 30 days before interview	Middle (Adhibasi/Janajati)	**56.2%**	
				Low (Dalits)	**42.9%**	
		**Education**	**Alcohol use**	Low (No formal education)	**58.5%**	<0.001
				Middle-low (lower than primary)	**55.2%**	
				Middle-high (primary)	**36.1%**	
				High (Secondary and higher)	**26.0%**	
		**SES (education, occupation, income]**	**Alcohol use**	Lowest	**57.4%**	0.012
				Middle-low	**42.6%**	
				Middle-high	**53.1%**	
no adjustment described				Highest	**31.2%**	

Diet

Hashibe et al. (2003) - Funded by Assoc. Int Cancer Research; Imperial Cancer Research Fund; National Cancer Institute (USA) & UCLA Jonnson Cancer Centre Foundation

case-control	Study examining SES and	**Income**	**Fruits - high intake**	Low (INR<1500	**87.5%**	
47,773	premalignant oral lesions in			Middle-low (INR 1500-3000)	**92.1%**	
India	premalignant oral lesions in			Middle-high (INR 3001-5000)	**93.0%**	
	Kerala. Data is presented			High (INR>5000)	**93.9%**	<0.0001
	linking NCD risk factors with					
	SES markers for the 47,773	**Education**	**Fruits - high intake**	Low (None/illiterate)	**79.4%**	
	controls only.			Middle-low (None/literate)	**94.0%**	
	Aged >35 years			Middle (Primary)	**89.4%**	
				Middle-high (Middle school)	**90.9%**	
				High (>High schools)	**93.5%**	<0.0001
		**Occupation**	**Fruits- high intake**	Manual	**89.4%**	
				Teacher/office	**95.2%**	
				Business	**90.8%**	
				Retired	**86.1%**	
				Other	**94.9%**	<0.0001

		**Income**	**Daily vegetables**		Low (INR<1500	**88.6%**	
					Middle-low (INR 1500-3000)	**96.2%**	
					Middle-high (INR 3001-5000)	**97.5%**	
					High (INR>5000)	**98.5%**	<0.0001
		**Education**	**Daily vegetables**		None/illiterate	**80.6%**	
					None/literate	**92.4%**	
					Primary	**91.6%**	
					Middle	**94.1%**	
					>High schools	**97.1%**	<0.0001
		**Occupation**	**Daily vegetables**		Manual	**91.3%**	
					Teacher/office	**98.7%**	
					Business	**95.6%**	
					Retired	**94.6%**	
no adjustment described					Other	**96.8%**	<0.0001

<u>Gupta et al. (2012)</u> - Funded by the South Asian Society of Atherosclerosis and Thrombosis

cross-sectio	General population in	**Education**	**High dietary fat**	1,248	Low (0-10 years)	**46.2%**	
6,198	middle-class areas of 11		>20g/day	2,956	Middle (11-15 years)	**49.8%**	
India	cities, excluded house-			1,366	High (>15 years)	**60.0%**	
	bound, pregnant and those						
	likely to die within 6	**Occupational Class**	**High dietary fat**	1,287	Low (4-5)	**40.6%**	
	months.	(British Social Register,		1,677	Middle (3 manual/non-manual)	**49.7%**	
	Aged 18-75 years	housewife = husband)		3,018	High (1-2)	**55.7%**	
		**SES (self-assessed)**	**High dietary fat**	374	Low (score 1-3)	**41.8%**	
				3,622	Middle (score 4-6)	**53.1%**	
				1,114	High (score 7-10)	**61.6%**	
		**Education**	**Low fruit and vegetables**	1,248	Low (0-10 years)	**23.9%**	
			<2 servings/day	2,956	Middle (11-15 years)	**29.3%**	

				1,366	High (>15 years)	**28.9%**	
		**Occupational Class** (British Social Register, housewife = husband)	**Low fruit and vegetables**	1,287	Low (4-5)	**26.9%**	
				1,677	Middle (3 manual/non-manual)	**26.8%**	
				3,018	High (1-2)	**26.4%**	
		**SES (self-assessed)**	**Low fruit and vegetables**	374	Low (score 1-3)	**47.8%**	
				3,622	Middle (score 4-6)	**24.7%**	
adjusted for age and sex				1,114	High (score 7-10)	**21.8%**	

<u>Agrawal et al. (2014a)</u> - Funded by a Wellcome Trust Strategic Award Grant

ross-sectiona	Nationally representative	**Caste**	**Non-vegetarian**	29,831	Scheduled caste (poorest)	**74.0%**	<0.001
156,317	2005/6 National Family Health Survey, participants aged		eats meat, fish, milk, eggs, curd, dairy products	12,734	Scheduled tribe (poorest)	**75.2%**	
India	20-49 years			60,977	Other backward class (poor)	**60.4%**	
				48,854	General (more affluent)	**57.7%**	
		**SES (assets and housing)**	**Non-vegetarian**	26,389	Lowest	**71.1%**	<0.001
				28,751	Low	**66.2%**	
				31,232	Middle	**65.4%**	
				33,560	High	**64.1%**	
no adjustment described				36,385	Highest	**54.0%**	

<u>Agrawal et al. (2014b)</u> - Funded by the Wellcome Trust; Council for England; National Institute for Health Research Collaboration

ross-sectiona	Nationally representative	**Caste**	**Daily fish consumption**		Low caste	**3.4%**	<0.001
156,317	2005/6 National Family Health Survey, participants aged				Other caste	**44.0%**	
India	20-49 years						
		**Wealth (assets and housing)**	**Daily fish consumption**		Highest (top quintile)	**39.1%**	<0.001
no adjustment described					Lowest (bottom quintile)	**8.2%**	

<u>Ganesan et al. (2012)</u> - No external funding

All High Quality Studies

cross-sectional 1,261	1,261 Diabetics and 122 non-diabetics from the population-based urban Sankara Nethralaya Diabetic Retinopathy Epidemiology and Molecular Genetic Study. Aged >40 years							
		**SES (undefined)**	**Low fibre diet**		Low	**11.9%**		0.002
India			fibre score <32 on validated questionnaire		Middle	**71.9%**		0.237
					High	**16.1%**		<0.0001
		**SES (undefined)**	**High fibre diet**		Low	**6.5%**		0.002
			fibre score >32 on validated questionnaire		Middle	**68.8%**		0.237
no adjustment described					High	**24.6%**		<0.0001

Kinra et al. (2010) - Funded by the Wellcome Trust

ross-section	Nationally representative	**SES (men) assets, housing**	**Low fruit and vegetable intake**	147	Low (asset score)	**81.0%**	74.5-87	<0.001
1,983	sample of rural inhabitants		<400g/day	358	Middle (asset score)	**75.6%**	71.2-79.9	
India	from 1600 villages in 18 states.			870	High (asset score)	**63.6%**	60.3-66.8	
	Aged 20-69 years	**SES (women) assets, housing**	**Low fruit and vegetable intake**	106	Low (asset score)	**86.6%**	77.5-95	<0.001
				143	Middle (asset score)	**78.5%**	71.4-85.5	
adjusted for age				359	High (asset score)	**69.9%**	65.2-74.7	

Dhungana et al. (2014) - Source of funding not reported

ross-section	Rural community in the	**Caste**	**Low fruit and vegetables**		High (Brahman/Chhetri)	**100.0%**		0.014
406	Sindhuli district.		<400g/day		Middle (Adhibasi/Janajati)	**94.5%**		
Nepal	Aged 20-50 years				Low (Dalits)	**100.0%**		
		**Education**	**Low fruit and vegetables**		Low (No formal education)	**97.9%**		0.32
					Middle-low (lower than primary)	**93.1%**		
					Middle-high (primary)	**97.2%**		
					High (Secondary and higher)	**95.5%**		

		**SES** (education, occupation, income)	**Low fruit and vegetables**	Lowest	**96.7%**		0.001
				Middle-low	**98.9%**		
				Middle-high	**96.9%**		
no adjustment described				Highest	**81.2%**		

Zeba et al. (2014) - Funded by the Canadian International Development Agency

cross-sectional 300	Burkinabe born and resident in Ouagadougou for >6 months. Excludes pregnant or lactating women and physically and mentally disabled individuals.	**Asset score**	**Unhealthy diet** high in fat, sugar, low in fibre, plant protein and complex carbohydrates	Low (tertile score)	**19.1%**	11.1-27	0.002
				Medium (tertile score)	**37.1%**	27.1-47	0.4
Burkina Faso	Aged 25-60 years			High (tertile score)	**43.8%**	33.5-54	0.003
		**Education**	**Unhealthy diet**	Low (no formal)	**24.7%**	15.8-33	<0.001
				Medium (elementary)	**15.7%**	8.2-23.[illegible]	0.3
no adjustment described				High (High school and above)	**59.6%**	49.4-69	<0.001

Tobaco

Bonu et al. (2005) - Source of funding not reported

cross-sectional 22,685	Hospitalised participants of the 1995/6 Indian National Sample Survey. Aged >10 years	**Current regular tobacco user**	**Poverty**	Non-user	**1**	OR	
India			borrowing/financial distress during hospitalisation	Tobacco user	**1.35**	1.11-1.[illegible]	<0.01

adjusted for; head of household, age, sex, level of schooling, marital status, state. number of days of hospitalisation used to control for severity of illness.

Hashibe et al. (2003) - Funded by Assoc. Int Cancer Researc; Imperial Cancer Research Fund; National Cancer Institute (USA) & UCLA Jonnson Cancer Center Foundation

case-control 47,773	Study examining SES and	**Income**	**Tobacco chewing**	Low (INR<1500	**34.3%**		
	premalignant oral lesions in			Middle-low (INR 1500-3000)	**22.9%**		
India	Kerala. Data is presented			Middle-high (INR 3001-5000)	**17.0%**		

	linking NCD risk factors with SES markers for the 47,773 controls only. Aged >35 years				High (INR>5000)	**11.2%**	<0.0001
		**Education**	**Tobacco chewing**		Low (None/illiterate)	**48.2%**	
					Middle-low (None/literate)	**45.7%**	
					Middle (Primary)	**33.7%**	
					Middle-high (Middle school)	**26.4%**	
					High (>High schools)	**12.4%**	<0.0001
		**Occupation**	**Tobacco chewinhg**		Manual	**29.0%**	
					Teacher/office	**10.9%**	
					Business	**21.1%**	
					Retired	**34.9%**	
					Other	**23.8%**	<0.0001
		**Income**	**Smoking**		Low (INR<1500	**28.8%**	
					Middle-low (INR 1500-3000)	**22.9%**	
					Middle-high (INR 3001-5000)	**20.2%**	
					High (INR>5000)	**16.6%**	<0.0001
		**Education**	**Smoking**		Low (None/illiterate)	**19.7%**	
					Middle-low (None/literate)	**23.3%**	
					Middle (Primary)	**29.4%**	
					Middle-high (Middle school)	**30.9%**	
					High (>High schools)	**23.8%**	<0.0001
		**Occupation**	**Smoking**		Manual	**22.6%**	
					Teacher/office	**28.4%**	
					Business	**54.3%**	
					Retired	**40.7%**	
No adjustment described					Other	**52.3%**	<0.0001

Corsi et al. (2014) - Funded by Byrraju Found; Initiative for CV Health Research in Dev. Countries; National Health and Med Research Council (AUS); the George Found.

cross-sectio	Representative sample from	**Education (men)**	**Ever smoker**	2,205	Low (None/Illiterate)	**64.0%**	

4,534<br>India	20 villages in rural Andhra Pradesh.<br>Aged >20 years		current or former smoker		Medium (Primary)	**57.9%**		
					High (Secondary or higher)	**37.7%**		
		**Education (women)**	**Ever smoker**	2,329	Low (None/Illiterate)	**12.4%**		
					Medium (Primary)	**3.2%**		
					High (Secondary or higher)	**1.1%**		
		**Education**	**Current smoker**	4,535	Low (None/Illiterate)	**3.25**	[illegible].54-4.1[illegible]	<0.01
					Medium (Primary)	**1.87**	[illegible].13-2.44	
					High (Secondary or higher)	**1**	OR	
		**Income (men)**	**Ever smoker**	2,205	Extremely Low (<US$0.50/day)	**62.2%**		
					Very Low ($0.50-1/day)	**57.5%**		
					Low (US$1-2/day)	**55.9%**		
					Higher (US$>2/day)	**50.7%**		
		**Income (women)**	**Ever smoker**	2,329	Extremely Low (<US$0.50/day)	**19.1%**		
					Very Low ($0.50-1/day)	**7.3%**		
					Low (US$1-2/day)	**7.7%**		
					Higher (US$>2/day)	**4.3%**		
		**Income (men)**	**Current smoker**	4,535	Extremely Low (<US$0.50/day)	**1.5**	[illegible].06-2.1[illegible]	<0.05
					Very Low ($0.50-1/day)	**1.15**	[illegible].88-1.49	
					Low (US$1-2/day)	**1.12**	[illegible].89-1.42	
No adjustment described for percentages					Higher (US$>2/day)	**1**	OR	
Odds ratios adjusted for age, sex, occupation, income								

Kinra et al. (2010) - Funded by the Wellcome Trust

cross-sectional<br>1,983	Nationally representative sample of rural inhabitants from 1600 villages in 18 states.	**SES (men) assets, housing**	**Smoker**	147	Low (asset score)	**36.8%**	29.6-44	<0.001

India	Aged 20-69 years		daily tobacco smoking at any time in the last 6 months	358	Middle (asset score)	**28.1%**	23.5-32.7	
				870	High (asset score)	**14.7%**	12.3-17.1	
		**SES (women) assets, housing**	**Smoker**	106	Low (asset score)	**1.2%**	0-2.9	0.2
				143	Middle (asset score)	**1.1%**	0-2.6	
adjusted for age				359	High (asset score)	**0.3%**	0-0.8	

<u>Neufeld et al. (2005)</u> - No external funding

ross-section: 471,143 India	Data from the 1995/6 Indian National Sample Survey. Aged >10 years	**Poverty***	**Smoke tobacco** regular use, any form	303416	High (above poverty line)	**1**	OR	
				167,727	Low (below poverty line)	**1**	1-1.1	
		**Caste****	**Smoke tobacco**	334,512	High	**1**	OR	
				136,631	Low	**1.4**	1.3-1.5	
		**Education**	**Smoke tobacco**	273,069	High (formal education)	**1**	OR	
				188,956	Low (no formal education)	**1.7**	1.6-1.8	
		**Poverty***	**Chew tobacco**	303,416	High (above poverty line)	**1**	OR	
			regular use, any form	167,727	Low (below poverty line)	**1.5**	1.4-1.6	
		**Caste****	**Chew tobacco**	334,512	High	**1**	OR	
				136,631	Low	**1.5**	1.4-1.6	
		**Education**	**Chew tobacco**	273,069	High (formal education)	**1**	OR	
				188,956	Low (no formal education)	**1.2**	1.1-1.3	
adjusted for age group, gender caste, income, residence, education								

**Planning commission of india definition - income required to ensure adequate intake of calories (INR 2,100 urban; INR 2,400 rural)*

***Scheduled Castes and tribes - identified in the Indian Constituion as especially disadvantaged or needy*

<u>Gupta et al. (2003)</u> - Source of funding not reported

cross-sectional 573	Serial cross-sectional surveys from the general population of Jaipur. Data taken from the most recent round.	**Education (men)**	**Smoker**	103	Low (no formal education)	**54.4%**	
India	Age not reported		past or present use of any tobacco product	182	Middle-low (1-10 years)	**42.9%**	
				202	Middle-high (11-15 years)	**28.7%**	
				63	High (>16 years)	**23.8%**	
		**Education (women)**	**Smoker**	213	Low (no formal education)	**28.2%**	
				163	Middle-low (1-10 years)	**3.1%**	
				161	Middle-high (11-15 years)	**0.6%**	
adjusted for age				36	High (>16 years)	**2.8%**	

<u>Singh et al. (2000)</u> - Sandoz (Novartis) Foundation of Gerontologic Research (AUS); World Health Federation

cross-sectional 1,767	Residents of two villages in rural north India.	**SES (education, occupation, income, assets, housing)**	**Use tobacco**	985	High	**8.1%**	0.09
	Aged 25-64 years		uses tobacco >1/week	790	Medium-high	**5.9%**	
India				774	Medium	**6.7%**	
				602	Medium-low	**7.9%**	
No adjustment described				206	Low	**8.7%**	

<u>Gupta et al. (2012)</u> - Funded by the South Asian Society of Atherosclerosis and Thrombosis

cross-sectional 6,198	General population in	**Education**	**Tobacco use**	1,248	Low (0-10 years)	**24.3%**	
	middle-class areas of 11		daily use of a tobacco product	2,956	Middle (11-15 years)	**14.4%**	
	cities, excluded house-			1,366	High (>15 years)	**19.0%**	
	bound, pregnant and those						
	likely to die within 6 months.	**Occupational Class (British Social Register, housewife = husband)**	**Tobacco use**	1,287	Low (4-5)	**16.1%**	
India	Aged 18-75 years			1,677	Middle (3 manual/non-manual)	**20.3%**	
				3,018	High (1-2)	**16.7%**	
		**SES (self-assessed)**	**Tobacco use**	374	Low (score 1-3)	**17.6%**	

				3,622	Middle (score 4-6)	**19.6%**		
adjusted for age and sex				1,114	High (score 7-10)	**15.5%**		

Reddy et al. (2007) - Funded by the Indian Ministry of Health; WHO

cross-sectional- 19,969<br>India	Industrial workers and their relatives from ten urban sites across India. Aged 20-69 years.	**Education (men)**	**Tobacco use**	1,611	High (postgraduate)	**1**	OR	<0.001
			use of any tobacco product in previous 30 days	2,607	Middle-high (secondary-tertiary)	**1.3**	1.1-1.5	
				5,820	Midle-low (primary-secondary)	**1.9**	1.6-2.2	
				1,859	Low (none-primaryl)	**6.5**	5.2-8.1	
		**Education (women)**	**Tobacco use**	960	High (postgraduate)	**1**	OR	<0.001
				1,635	Middle-high (secondary-tertiary)	**1.1**	0.8-1.3	
				2,832	Midle-low (primary-secondary)	**1.1**	0.76-1.4	
adjusted for age and occupation				2,645	Low (none-primaryl)	**8.2**	6.4-9.9	

Singh et al. (2007) - Funded by the Center of Nutrition Research, International College of Nutrition (India)

cross-sectional 2,222<br>India	Spouses/relatives surveyed about their deceased from 1999-2001, who lived in Moradabad. Aged 25-64 years	**SES (men) education, occupation, income, assets, housing**	**Tobacco consumption**	264	Highest	**58.0%**		>0.05*
			use of tobacco product	345	High	**51.0%**		
				290	Middle	**47.6%**		
				277	Low	**42.6%**		
				209	Lowest	**51.2%**		
		**SES (men) education, occupation, income, assets, housing**	**Tobacco consumption**	163	Highest	**13.5%**		<0.05*
			use of tobacco product	221	High	**15.4%**		
				169	Middle	**11.2%**		
				159	Low	**19.5%**		
				125	Lowest	**21.6%**		
No adjustment described					*comparing highest-middle vs low and lowest			

<u>Samuel et al. (2012)</u> - Funded by the British Heart Foundation

cross-sectional 2,218	Young adults from population-based birth cohort in rural and urban areas of southern India. Aged 26-32 years	**Wealth (asset score)**	**Tobacco use**		Lowest (quintile 1)	**1**	OR	
India			current user		Low (quinile 2)	**0.6**	0.4-0.9	
					Middle (quintile 3)	**0.5**	0.3-0.7	
					High (quinile 4)	**0.5**	0.4-0.9	
					Highest (Quintile 5)	**0.4**	0.2-0.6	
		**Education**	**Tobacco use**		Low (0 years formal schooling)	**1**	OR	
					Middle-low (1-8 years)	**0.8**	0.5-1.4	
adjusted for gender, place of residence, posessions score, adult educational status, paternal education					Middle-high (9-12)	**0.5**	0.3-0.9	
Multiple logistc regression analysis; gender, place of residence, posessions score, adult educational status, paternal educational status					High (>12 years)	**0.2**	0.1-0.4	

<u>Gupta et al. (2015)</u> - Funded by the South Asian Society of Atherosclerosis and Thrombosis

cross-sectional 6,198	General population in middle-class areas of 11 cities, excluded house-bound, pregnant and those likely to die within 6 months. Aged >20 years	**Education**	**Quit tobacco**	1,248	Low (0-10 years)	**1.6%**		0.139
India			quit for >1year having used for >1 year previously	2,956	Middle (11-15 years)	**2.8%**		
adjusted for age and sex				1,366	High (>15 years)	**5.5%**		

<u>Jena et al. (2012)</u> - Source of funding not reported

cross-sectional 69,296	Smokers from 29 territories, from the nationally representative 2009 Indian Global Adult Tobacco Survey. Aged >15 years	**Occupation**	**Hardcore smoker***		Employee	**1**	OR	
India					Student	**2.40**	1.16-4.9[illegible]	0.018
					Self-employed	**2.64**	1.28-5.4[illegible]	0.008
					Homemaker	**2.49**	1.18-5.2[illegible]	0.017

				Retired or unemployed	**2.24**	[illegible].07-4.6[illegible]	0.033
		**Education**	**Hardcore smoker***	Low (no formal education)	**1**	OR	
Multivarate logistic regression, variables not reported				Medium-low (primary incomplete)	**0.96**	[illegible].84-1.1[illegible]	0.569
**Proportion of daily smokers who have not attempted to quit in prev 12 months/ last attempt was <24h and no intention to quit in next 12 month[illegible]*				Medium (primary but secondary incon[illegible]	**1.01**	[illegible].90-1.1[illegible]	0.900
if at all, smokes within 30 mins of waking and knows smoking is harmful				High (secondary and above)	**1.00**	[illegible].85-1.1[illegible]	0.990

<u>**Narayan et al. (1996)**</u> - Funded by the Sitaram Bhartia Institute of Science and Research

cross-sectional 13,558 India	Residents of Delhi. Aged 25-64 years	**Education (men)**	**Smoker** currently smoking or had smoked >100 cigarettes or beedis	Low (none)	**1.75**	1.52-2.02	
				Middle-low (primary)	**1.29**	1.12-1.48	
				Middle-low (primary)	**1.06**	0.94-1.19	
				Middle-high (secondary)	**0.84**	0.75-0.94	
				High (college)	**1**	OR	
		**Education (women)**	**Smoker**	Low (none)	**3.72**	2.66-4.82	
				Middle-low (primary)	**1.13**	0.79-1.63	
				Middle-low (primary)	**0.94**	0.59-1.50	
				Middle-high (secondary)	**0.44**	0.24-0.79	
				High (college)	**1**	OR	
		**Occupation (men)**	**Smoker**	High (I)	**1**	OR	
				Medium-high (II)	**1.15**	0.96-1.38	
				Medium-low (III)	**0.8**	0.63-1.02	
				Low (IV)	**1.39**	1.13-1.70	
		**Occupation (women)**	**Smoker**	High (I)	**1**	OR	
				Medium-high (II)	**1.47**	0.47-4.62	
				Medium-low (III)	**0.87**	0.32-2.41	
				Low (IV)	**1.91**	0.64-5.70	
adjusted for income, education, marital status, religion, occupation, physical activity, leuisure activity, BMI, drinking status, meat intake, egg eating, vegitarian status, family history heart disease							

Rani et al. (2003) - Source of funding not reported

ross-section	Data from the 1998/9	**Household wealth (men)**	**Smoke tobacco**	Highest quintile	**1**	OR	ref
334,553	National Family Health	**dwelling and assets**		Second quintile	**1.53**		<0.001
India	Survey.			Middle quintile	**1.94**		<0.001
	Aged >15 years			Fourth quintile	**2.11**		<0.001
				Lowest quintile	**2.26**		<0.001
		**Household wealth (men)**	**Chew tobacco**	Highest quintile	**1**	OR	ref
		**dwelling and assets**		Second quintile	**1.4**		<0.001
				Middle quintile	**1.55**		<0.001
				Fourth quintile	**1.69**		<0.001
				Lowest quintile	**1.93**		<0.001
		**Household wealth (women)**	**Smoke tobacco**	Highest quintile	**1**	OR	
		**dwelling and assets**		Second quintile	**1.57**		<0.001
				Middle quintile	**2.68**		<0.001
				Fourth quintile	**3.26**		<0.001
				Lowest quintile	**4.32**		<0.001
		**Household wealth (women)**	**Chew tobacco**	Highest quintile	**1**	OR	
		**dwelling and assets**		Second quintile	**1.52**		<0.001
				Middle quintile	**1.92**		<0.001
				Fourth quintile	**2.15**		<0.001
				Lowest quintile	**2.58**		<0.001
		**Education (men)**	**Smoke tobacco**	High (>11 years)	**1**	OR	
				Medium-high (6-10 years)	**1.84**		<0.001
				Medium-low (1-5 years)	**2.72**		<0.001
				Low (No formal education)	**3.17**		<0.001
		**Education (men)**	**Chew tobacco**	High (>11 years)	**1**	OR	
				Medium-high (6-10 years)	**1.48**		<0.001
				Medium-low (1-5 years)	**1.86**		<0.001

			Low (No formal education)	**1.92**		<0.001
	**Education (women)**	**Smoke tobacco**	High (>11 years)	**1**	OR	
			Medium-high (6-10 years)	**1.73**		>0.05
			Medium-low (1-5 years)	**2.82**		<0.05
			Low (No formal education)	**6.25**		<0.001
	**Education (women)**	**Chew tobacco**	High (>11 years)	**1**	OR	
			Medium-high (6-10 years)	**2.05**		<0.001
			Medium-low (1-5 years)	**3.81**		<0.001
			Low (No formal education)	**4.97**		<0.001
	**Caste (men)**	**Smoke tobacco**	High (Forward/general caste)	**1**	OR	
			Medium (Other backward caste)	**1.01**		>0.05
			Low (Scheduled caste)	**1.2**		<0.001
			Low (Scheduled tribe)	**1.05**		>0.05
	**Caste (men)**	**Chew tobacco**	High (Forward/general caste)	**1**	OR	
			Medium (Other backward caste)	**1.07**		<0.05
			Low (Scheduled caste)	**1.12**		<0.05
			Low (Scheduled tribe)	**1.23**		<0.001
	**Caste (women)**	**Smoke tobacco**	High (Forward/general caste)	**1**	OR	
			Medium (Other backward caste)	**1.09**		<0.001
			Low (Scheduled caste)	**1.34**		>0.05
			Low (Scheduled tribe)	**1.49**		<0.05
	**Caste (women)**	**Chew tobacco**	High (Forward/general caste)	**1**	OR	
			Medium (Other backward caste)	**1.14**		>0.05
			Low (Scheduled caste)	**1.62**		<0.001
adjusted for wealth, years of schooling, religon, caste, age, sex, urban/rural			Low (Scheduled tribe)	**2.49**		<0.001

Heck et al. (2012) - Funded by US National Institutes of Health

cross-sectional 19,934 Bangladesh	Married Bangladeshi adults from a longitudinal arsenic study (HEALS). Age 18-75 years	**Education**	**Betel quid** use with or without tobacco (82.5% use it with tobacco)		Low (no formal)	**2**	1.81-2.20	
					Middle (1-5 years)	**1.65**	1.49-1.82	
					High (>6 years)	**1**	OR	
adjusted for gender, age, marital status, occupation, religion, land ownership, TV ownership, smoking pack years								

Kishore et al. (2013) - Source of funding not reported

cross-sectional	Data from Global Adult	**Education (India)**	**Hardcore smoker***	24,309,857	High (college and above)	**1**	OR	
92,491	Tobacco Survey data from India 2009/10 (69,296 individuals), Thailand 2009 (20,566 individuals), Bangladesh 2009 (9,629 individuals).		odds of daily smoker becoming a hardcore smoker		Medium-high (higher second)	**0.98**	0.66-1.[illegible]	0.91
India, Thaila	Aged >15 years				Medium-low (up to primary)	**0.96**	0.62-1.[illegible]	0.85
					Low (no formal education)	**1.1**	0.72-1.[illegible]	0.65
		**Wealth Index (India)** principal component analysis	**Hardcore smoker**	24,309,857	Highest	**1**	OR	
					Lowest	**1.27**	0.9-1.[illegible]	0.17
					Second	**1.29**	0.92-1.[illegible]	0.14
					Middle	**1.17**	0.85-1.[illegible]	0.33
					Fourth	**1.08**	0.80-1.[illegible]	0.6
		**Education (Bangladesh)**	**Hardcore smoker**	3,651,921	High (college and above)	**1**	OR	
					Medium-high (higher second)	**2.39**	0.83-6.[illegible]	0.11
					Medium-low (up to primary)	**1.9**	0.65-5.[illegible]	0.24
					Low (no formal education)	**2.26**	0.78-6.[illegible]	0.13
		**Wealth Index (Bangladesh)** principal component analysis	**Hardcore smoker**	3,651,921	Highest	**1**	OR	
					Lowest	**3.15**	1.67-5.[illegible]	0
					Second	**2.68**	1.12-5.[illegible]	0
					Middle	**1.93**	1.07-3.[illegible]	0.03

					Fourth	**1.49**	0.83-2.[illegible]	0.18
		**Education (Thailand)**	**Hardcore smoker**	3,180,566	High (college and above)	**1**	OR	
					Medium-high (higher second)	**0.9**	0.59-1.[illegible]	0.63
					Medium-low (up to primary)	**1.07**	0.70-1.[illegible]	0.76
					Low (no formal education)	**0.96**	0.51-1.[illegible]	0.89
		**Wealth Index (Thailand)**	**Hardcore smoker**	3,180,566	Highest	**1**	OR	
		principal component analysis			Lowest	**1.28**	0.92-1.[illegible]	0.14
No adjustment described					Second	**1.04**	0.75-1.[illegible]	0.83
**current daily smoking, no quit attempt in last 12 months or last quit was <24h, no intention to quit in next 12 months or not interested in quitting*					Middle	**1**	0.75-1.[illegible]	0.98
first smoke within 30mins of waking, knowledge of harms					Fourth	**1.08**	0.8-1.4[illegible]	0.63

Dhungana et al. (2014) - Source of funding not reported

cross-sectional 406	Rural community in the Sindhuli district.	**Caste**	**Smoker**		High (Brahman/Chhetri)	**26.2%**		0.79
	Aged 20-50 years		Smoking until last 30 days befor interview		Middle (Adhibasi/Janajati)	**29.7%**		
Nepal					Low (Dalits)	**28.6%**		
		**Education**	**Smoker**		Low (no formal education)	**44.7%**		<0.001
					Middle-low (lower than pimary)	**17.2%**		
					Middle-high (primary)	**8.3%**		
					High (secondary and higher)	**18.2%**		
		**SES**	**Smoker**		Lowest	**42.6%**		<0.001
		(education, occupation, income)			Middle-low	**27.7%**		
					Middle-high	**18.8%**		
No adjustment described					Highest	**0.0%**		

Bovet et al. (2002) - Funded by the Swiss National Science Foundation

cross-sectional 9,254	Rural community in the Sindhuli district.	**Education**	**Smoker**		Low (none)	**1**	OR	

Tanzania	Aged 20-50 years		≥1 cigarette/day		Middle-low (primary)	**0.93**	0.622
					Middle-high (secondary)	**0.53**	<0.001
					High (tertiary)	**0.56**	<0.002
		**Wealth (asset score)**	**Smoker**		Lowest	**1**	OR
					Second	**0.87**	0.131
					Third	**0.46**	<0.001
					Fourth	**0.42**	<0.001
					Fifth	**0.58**	0.001
adjusted for age, sex, occucation, wealth					Highest	**0.48**	<0.001
**Minh et al. (2007)** - Funded by the International Network of Demographic Evaluation of Populations (INDEPTH); Swedish Council for Social and Work Life Research							
cross-sectional 1,984 Vietnam	Representative 2005 STEPS survey in Bavi district of northern Vietnam. Aged 25-64 years	**Education**	**Smoker**		Low (<7 years)	**0.9**	0.6-1.4
					Middle (7-9 years)	**0.8**	0.6-1.2
					High (>9 years)	**1**	OR
		**SES** (local authority assessment and rice production)	**Smoker**		low	**2**	1.2-3.4
					middle	**1.4**	1.1-2
					high	**1**	OR
adjusted for	Sex, Age, Education, Occupation, and Economic Status						
**Tonstad et al. (2013)** - Funded by the National Institutes of Health; The Fogarty International Center							
cross-sectional 5,592 Cambodia	Data from smokers identified in the 2006 National Tobacco Survey. Aged >18 years	**Education**	**Quit tobacco** not used tobacco products for >2 years among ever-users	4,727	Low (<6 years)	**1**	OR
				840	High (>7 years)	**1.46**	1.01-2.13
		**Income**	**Quit tobacco**	3,919	Low (<US$1/day)	**1**	OR
				1,673	High (>US$1/day)	**1.39**	1.01-1.91
		**Ocupation**	**Quit tobacco**	616	None	**1**	OR
				93	Profeessional	**2.52**	1.27-5.01

				167	Technichal/Service	**1.32**	0.7-2.51
adjusted for age, demographics, health status characteristics				467	Labour	**1.98**	1.1-3.56

Ali et al. (2006) - Source of funding not reported

cross-sectional 411	Men from a rural area of Sindh province.	**Education**	**Smoker**	104	High (>10 years)	**1**	OR
Pakistan	Aged >18 years		has smoked >100 cigarettes	95	Medium-high (6-10 years)	**1.1**	0.9-1.6
				92	Medium-low (1-5 years)	**1.1**	0.9-1.4
				120	Low (Illiterate)	**1.1**	0.9-1.5
		**Income (average, individual)**	**Smoker**	98	Low (no income)	**1**	OR
				114	Medium-low (<US$30/month)	**1.4**	1.0-2.1
				151	Medium-high (US$30-60/month)	**1.4**	1.0-2.1
adjusted for age, income, marital status				48	High (>US$60/month)	**1.7**	1.2-2.6

Hosseinpoor et al. (2012) - Funded by WHO

cross-sectional	Reports data from 48 LMICs						Standard Error
213,807	taken from the 2003 World	**SES (men) assets, services**	**Smoker**	**Georgia**	Lowest	**50.1%**	6
Global	Health Survey		daily or occasional tobacco smoker		Low	**59.0%**	4.3
	Aged >18 years				Medium	**58.3%**	3.5
					High	**63.4%**	4
					Highest	**67.2%**	2.8
		**SES (women) assets, services**	**Smoker**	**Georgia**	Lowest	**1.9%**	1.1
			daily or occasional tobacco smoker		Low	**2.4%**	1.1
					Medium	**4.8%**	1.8
					High	**8.2%**	2.5
					Highest	**11.6%**	2.6
							Standard Error
		**SES (men) assets, services**	**Smoker**	**Morocco**	Lowest	**40.3%**	4.7
			daily or occasional tobacco smoker		Low	**34.0%**	3.8
					Medium	**38.4%**	5.4

			High	**31.2%**	4.8
			Highest	**17.5%**	3.5
**SES (women) assets, services**	**Smoker**	**Morocco**	Lowest	**0.0%**	0
	daily or occasional tobacco smoker		Low	**0.4%**	0.3
			Medium	**0.0%**	0
			High	**0.3%**	0.3
			Highest	**0.0%**	0
					Standard Error
**SES (men) assets, services**	**Smoker**	**Paraguay**	Lowest	**62.4%**	2.6
	daily or occasional tobacco smoker		Low	**47.9%**	2.6
			Medium	**43.8%**	2.8
			High	**28.4%**	2.5
			Highest	**33.1%**	2.9
**SES (women) assets, services**	**Smoker**	**Paraguay**	Lowest	**17.9%**	1.9
	daily or occasional tobacco smoker		Low	**16.3%**	1.9
			Medium	**14.3%**	1.8
			High	**9.7%**	1.3
			Highest	**12.2%**	1.5
					Standard Error
**SES (men) assets, services**	**Smoker**	**Phillipines**	Lowest	**67.9%**	2.22
	daily or occasional tobacco smoker		Low	**60.6%**	2.4
			Medium	**57.3%**	2.2
			High	**55.6%**	2.2
			Highest	**50.2%**	2.3
**SES (women) assets, services**	**Smoker**	**Phillipines**	Lowest	**17.2%**	1.7
	daily or occasional tobacco smoker		Low	**14.4%**	1.4
			Medium	**12.1%**	1.2
			High	**12.1%**	1.3
			Highest	**8.8%**	1.3
					Standard Error

**SES (men) assets, services**	**Smoker**	**Sri Lanka**	Lowest	**56.1%**	4.5
	daily or occasional tobacco smoker		Low	**49.5%**	3.6
			Medium	**45.5%**	3.1
			High	**38.0%**	2.3
			Highest	**29.9%**	3.9
**SES (women) assets, services**	**Smoker**	**Sri Lanka**	Lowest	**6.4%**	1.9
	daily or occasional tobacco smoker		Low	**4.7%**	1.5
			Medium	**3.0%**	1
			High	**1.7%**	0.6
			Highest	**2.4%**	1
					Standard Error
**SES (men) assets, services**	**Smoker**	**Swaziland**	Lowest	**19.9%**	6.4
	daily or occasional tobacco smoker		Low	**10.7%**	2.8
			Medium	**14.8%**	5.4
			High	**14.3%**	5.2
			Highest	**16.5%**	3.9
**SES (women) assets, services**	**Smoker**	**Swaziland**	Lowest	**8.8%**	3.4
	daily or occasional tobacco smoker		Low	**1.7%**	0.9
			Medium	**0.2%**	0.2
			High	**4.1%**	1.6
			Highest	**2.3%**	2.3
					Standard Error
**SES (men) assets, services**	**Smoker**	**Ukraine**	Lowest	**55.3%**	6
	daily or occasional tobacco smoker		Low	**48.8%**	4.5
			Medium	**53.8%**	4.4
			High	**57.9%**	4
			Highest	**54.9%**	4.3
**SES (women) assets, services**	**Smoker**	**Ukraine**	Lowest	**7.7%**	1.8
	daily or occasional tobacco smoker		Low	**6.1%**	1.4
			Medium	**12.0%**	2.3

			High	**13.2%**	3
			Highest	**14.0%**	2.2
					Standard Error
**SES (men) assets, services**	**Smoker**	**Bangladesh**	Lowest	**72.2%**	2.9
	daily or occasional tobacco smoker		Low	**4.1%**	2.9
			Medium	**57.6%**	2.8
			High	**48.6%**	2.7
			Highest	**44.2%**	2.6
**SES (women) assets, services**	**Smoker**	**Bangladesh**	Lowest	**8.2%**	1.5
	daily or occasional tobacco smoker		Low	**6.0%**	1.2
			Medium	**8.3%**	1.5
			High	**5.9%**	1.6
					Standard Error
**SES (men) assets, services**	**Smoker**	**Burkina Faso**	Lowest	**29.9%**	3.4
	daily or occasional tobacco smoker		Low	**25.8%**	2.8
			Medium	**21.6%**	2.5
			High	**17.5%**	2.5
			Highest	**26.2%**	3.5
**SES (women) assets, services**	**Smoker**	**Burkina Faso**	Lowest	**12.6%**	2.4
	daily or occasional tobacco smoker		Low	**14.1%**	2.4
			Medium	**10.9%**	2.1
			High	**10.5%**	2.2
			Highest	**8.2%**	2.9
					Standard Error
**SES (men) assets, services**	**Smoker**	**Chad**	Lowest	**22.8%**	4
	daily or occasional tobacco smoker		Low	**19.9%**	3.2
			Medium	**19.3%**	3.2
			High	**18.2%**	2.3
			Highest	**14.8%**	2
**SES (women) assets, services**	**Smoker**	**Chad**	Lowest	**1.6%**	0.7

	daily or occasional tobacco smoker		Low	**5.1%**	2
			Medium	**2.7%**	1.1
			High	**3.8%**	2
			Highest	**3.6%**	1.2
					Standard Error
**SES (men) assets, services**	**Smoker**	**Comoros**	Lowest	**39.0%**	8
	daily or occasional tobacco smoker		Low	**37.9%**	6.5
			Medium	**31.6%**	4.6
			High	**39.5%**	6
			Highest	**32.4%**	5.5
**SES (women) assets, services**	**Smoker**	**Comoros**	Lowest	**38.3%**	8.7
	daily or occasional tobacco smoker		Low	**14.7%**	5.5
			Medium	**21.1%**	6.8
			High	**16.3%**	5.9
			Highest	**20.2%**	8.7
					Standard Error
**SES (men) assets, services**	**Smoker**	**Congo**	Lowest	**31.2%**	5.8
	daily or occasional tobacco smoker		Low	**28.6%**	4.8
			Medium	**17.7%**	4.5
			High	**10.7%**	3.4
			Highest	**9.9%**	4.2
**SES (women) assets, services**	**Smoker**	**Congo**	Lowest	**4.9%**	2.5
	daily or occasional tobacco smoker		Low	**0.9%**	0.5
			Medium	**2.9%**	1.8
			High	**1.4%**	0.7
			Highest	**0.3%**	0.3
					Standard Error
**SES (men) assets, services**	**Smoker**	**Cote d'Ivoire**	Lowest	**28.0%**	3
	daily or occasional tobacco smoker		Low	**21.3%**	2.8
			Medium	**22.0%**	2.8
			High	**19.5%**	2.8

			Highest	**18.0%**	33.1
**SES (women) assets, services**	**Smoker** daily or occasional tobacco smoker	**Cote d'Ivoire**	Lowest	**3.4%**	1.6
			Low	**5.1%**	1.8
			Medium	**3.9%**	1.3
			High	**1.8%**	0.8
			Highest	**1.1%**	0.6
					Standard Error
**SES (men) assets, services**	**Smoker** daily or occasional tobacco smoker	**Ethiopia**	Lowest	**5.3%**	2
			Low	**9.5%**	2.2
			Medium	**8.1%**	1.8
			High	**8.8%**	4.8
			Highest	**4.8%**	1.2
**SES (women) assets, services**	**Smoker** daily or occasional tobacco smoker	**Ethiopia**	Lowest	**0.4%**	0.4
			Low	**1.2%**	0.6
			Medium	**0.7%**	0.4
			High	**0.6%**	0.4
			Highest	**0.1%**	0.1
					Standard Error
**SES (men) assets, services**	**Smoker** daily or occasional tobacco smoker	**Ghana**	Lowest	**21.5%**	3
			Low	**12.7%**	1.9
			Medium	**9.6%**	1.8
			High	**6.1%**	1.3
			Highest	**8.4%**	1.8
**SES (women) assets, services**	**Smoker** daily or occasional tobacco smoker	**Ghana**	Lowest	**2.6%**	0.7
			Low	**1.4%**	0.7
			Medium	**0.9%**	0.7
			High	**0.7%**	0.4
			Highest	**1.3%**	0.5
					Standard Error
**SES (men) assets, services**	**Smoker**	**India**	Lowest	**46.7%**	3.2

	daily or occasional tobacco smoker		Low	**45.8%**	2.9
			Medium	**37.8%**	3.8
			High	**23.5%**	2.9
			Highest	**21.8%**	3.1
**SES (women) assets, services**	**Smoker**	**India**	Lowest	**12.4%**	3.7
	daily or occasional tobacco smoker		Low	**8.6%**	1.8
			Medium	**8.4%**	1.7
			High	**4.3%**	1.1
			Highest	**3.1%**	1
					Standard Error
**SES (men) assets, services**	**Smoker**	**Kenya**	Lowest	**33.1%**	5.4
	daily or occasional tobacco smoker		Low	**26.9%**	4
			Medium	**25.2%**	3.9
			High	**25.6%**	4
			Highest	**26.7%**	5.5
**SES (women) assets, services**	**Smoker**	**Kenya**	Lowest	**3.2%**	0.9
	daily or occasional tobacco smoker		Low	**3.3%**	1
			Medium	**3.8%**	2.3
			High	**0.8%**	0.4
			Highest	**0.2%**	0.2
					Standard Error
**SES (men) assets, services**	**Smoker**	**Lao People's**	Lowest	**77.1%**	2.9
	daily or occasional tobacco smoker		Low	**72.7%**	2.7
			Medium	**61.0%**	3.2
			High	**62.7%**	2.9
			Highest	**41.5%**	2.8
**SES (women) assets, services**	**Smoker**	**Lao People's**	Lowest	**28.3%**	3.6
	daily or occasional tobacco smoker		Low	**17.8%**	2.4
			Medium	**12.7%**	2.1

			High	**5.0%**	1.3
			Highest	**1.8%**	0.8
					Standard Error
**SES (men) assets, services**	**Smoker**	**Malawi**	Lowest	**40.9%**	3.2
	daily or occasional tobacco smoker		Low	**34.9%**	2.7
			Medium	**24.0%**	3.3
			High	**15.9%**	2.4
			Highest	**13.3%**	2.7
**SES (women) assets, services**	**Smoker**	**Malawi**	Lowest	**9.5%**	1.5
	daily or occasional tobacco smoker		Low	**7.5%**	1.6
			Medium	**6.8%**	1.5
			High	**3.5%**	1.1
			Highest	**0.7%**	0.5
					Standard Error
**SES (men) assets, services**	**Smoker**	**Mali**	Lowest	**27.3%**	3.2
	daily or occasional tobacco smoker		Low	**28.2%**	3.2
			Medium	**24.7%**	2.7
			High	**24.2%**	2.5
			Highest	**25.9%**	3.1
**SES (women) assets, services**	**Smoker**	**Mali**	Lowest	**4.0%**	1.6
	daily or occasional tobacco smoker		Low	**3.8%**	1.6
			Medium	**3.5%**	1.3
			High	**2.9%**	1.1
			Highest	**0.5%**	0.5
					Standard Error
**SES (men) assets, services**	**Smoker**	**Mauritania**	Lowest	**27.8%**	4.3
	daily or occasional tobacco smoker		Low	**22.3%**	4.3
			Medium	**23.4%**	4
			High	**30.6%**	4.2
			Highest	**38.6%**	4

**SES (women) assets, services**	**Smoker**	**Mauritania**	Lowest	**2.8%**	1.1
	daily or occasional tobacco smoker		Low	**1.6%**	0.8
			Medium	**5.6%**	2.2
			High	**7.2%**	1.8
			Highest	**5.8%**	1.5
					Standard Error
**SES (men) assets, services**	**Smoker**	**Myanmar**	Lowest	**52.5%**	3.9
	daily or occasional tobacco smoker		Low	**53.7%**	3.2
			Medium	**46.0%**	2.8
			High	**48.4%**	2.5
			Highest	**40.3%**	2.5
**SES (women) assets, services**	**Smoker**	**Myanmar**	Lowest	**21.8%**	2.5
	daily or occasional tobacco smoker		Low	**18.2%**	2.3
			Medium	**12.5%**	1.5
			High	**8.5%**	1.1
			Highest	**4.6%**	1
					Standard Error
**SES (men) assets, services**	**Smoker**	**Nepal**	Lowest	**43.7%**	2.6
	daily or occasional tobacco smoker		Low	**36.8%**	2.5
			Medium	**36.0%**	2.2
			High	**30.4%**	2.2
			Highest	**26.0%**	2
**SES (women) assets, services**	**Smoker**	**Nepal**	Lowest	**28.5%**	2.1
	daily or occasional tobacco smoker		Low	**25.6%**	2
			Medium	**18.7%**	1.5
			High	**17.9%**	1.8
			Highest	**9.7%**	1.3
					Standard Error
**SES (men) assets, services**	**Smoker**	**Pakistan**	Lowest	**40.5%**	2.5
	daily or occasional tobacco smoker		Low	**35.4%**	2.5
			Medium	**35.6%**	2.7

			High	**32.0%**	2.6
			Highest	**19.1%**	2.1
**SES (women) assets, services**	**Smoker** daily or occasional tobacco smoker	**Pakistan**	Lowest	**7.4%**	1.5
			Low	**6.8%**	1.3
			Medium	**7.4%**	1.8
			High	**6.3%**	1.6
			Highest	**3.8%**	1.2
					Standard Error
**SES (men) assets, services**	**Smoker** daily or occasional tobacco smoker	**Senegal**	Lowest	**28.9%**	4.6
			Low	**25.5%**	4.7
			Medium	**24.4%**	4
			High	**21.2%**	4.1
			Highest	**26.3%**	3.8
**SES (women) assets, services**	**Smoker** daily or occasional tobacco smoker	**Senegal**	Lowest	**4.7%**	2
			Low	**0.0%**	0
			Medium	**0.4%**	0.4
			High	**0.5%**	0.4
			Highest	**1.9%**	1.4
					Standard Error
**SES (men) assets, services**	**Smoker** daily or occasional tobacco smoker	**Vietnam**	Lowest	**66.9%**	3.5
			Low	**59.8%**	4.3
			Medium	**43.0%**	5.6
			High	**45.0%**	4.2
			Highest	**46.7%**	4.7
**SES (women) assets, services**	**Smoker** daily or occasional tobacco smoker	**Vietnam**	Lowest	**3.2%**	1.2
			Low	**2.1%**	0.8
			Medium	**2.2%**	0.8
			High	**3.4%**	1.4
			Highest	**1.8%**	1.2
					Standard Error

	**SES (men) assets, services**	**Smoker**	**Zambia**	Lowest	**36.8%**	3.5
		daily or occasional tobacco smoker		Low	**27.5%**	2.6
				Medium	**21.5%**	2.5
				High	**22.9%**	2.7
				Highest	**13.0%**	2.1
	**SES (women) assets, services**	**Smoker**	**Zambia**	Lowest	**11.5%**	1.6
		daily or occasional tobacco smoker		Low	**7.9%**	2.3
				Medium	**2.6%**	1
				High	**3.4%**	1.2
				Highest	**3.9%**	1.5
						Standard Error
	**SES (men) assets, services**	**Smoker**	**Zimbabwe**	Lowest	**37.3%**	4.5
		daily or occasional tobacco smoker		Low	**29.6%**	3.9
				Medium	**26.4%**	3.7
				High	**20.0%**	2.8
				Highest	**23.5%**	2.8
	**SES (women) assets, services**	**Smoker**	**Zimbabwe**	Lowest	**6.4%**	2
		daily or occasional tobacco smoker		Low	**3.6%**	1
				Medium	**3.1%**	0.8
				High	**1.9%**	0.7
No adjustment described				Highest	**1.7%**	0.8

Design, n	Quality	Sample	Exposure	Outcome	n	Exposure subgroup	Value	95% CI	p

Non peer-reviewed

WHO STEPS Eritrea (2004) - Funded by WHO, Italian Government, Eritrean Government

Design, n	Quality	Sample	Exposure	Outcome	n	Exposure subgroup	Value	95% CI	p
cross-sectional 2,033 Etitrea	moderate	Nationally representative sample, individuals selected from six zones. Aged 15-64 years	**Education**	**Alcohol use**	697	Lowest (no formal education)	**36.4%**		
					631	Low (Grades 1-6)	**37.9%**		
					247	Middle (Grade 7-8)	**44.5%**		
					386	High (Grades 9-12)	**44.8%**		
					90	Highest (>12 years)	**40.0%**		
						No adjustment described			

WHO STEPS Togo (2010) - Source of funding not reported

Design, n	Quality	Sample	Exposure	Outcome	n	Exposure subgroup	Value	95% CI	p
cross-sectional 4,370 Togo	moderate	Nationally representative STEPS survey. Residents from 6 zones who have lived there for >6 months. Aged 15-64 years	**Education**	**Harmful alcohol use** ≥60 g of pure alcohol/day for men, ≥40g/day for women	939	No formal education	**4.3%**	2.7-5.9	
					503	Primary	**2.3%**	0.8-3.8	
					430	Secondary	**3.3%**	1.3-5.4	
					276	College or equivalent	**2.9%**	0.8-5.1	
					73	High school	**3.2%**	0-7.2	
					55	University	**0.0%**	0-0	
						No adjustment described			

WHO STEPS Zambia (2008) - Funded by Zambian Ministry of Health; WHO

Design, n	Quality	Sample	Exposure	Outcome	n	Exposure subgroup	Value	95% CI	p
cross-sectional 1,928 Zambia	moderate	WHO STEPS survey in Lusaka district, 67% female. Aged >25 years	**Education**	**Alcohol consumption**		No formal education	**1**	OR	
						Primary completed	**1.51**	0.69-3.3	
						Secondary completed	**1.74**	0.92-3.3	
						College or higher completed	**1.67**	0.86-3.23	
						No adjustment described			

WHO STEPS India (2007) - Funded by World Bank, Indian Government

Design, n	Quality	Sample	Exposure	Outcome	n	Exposure subgroup	Value	95% CI	p
cross-sectional 38,064 India	moderate	WHO STEPS survey in seven states. Aged 15-64	**Education Andhra Pradesh**	**Alcohol use** any in last 12 months	6,218	Illiterate	**20.3%**		
						Primary	**24.6%**		
						Middle	**20.0%**		
						Secondary	**18.3%**		
						Higher Secondary	**17.6%**		
						College and above	**16.1%**		
			**Education Madhya Pradesh**	**Alcohol use** any in last 12 months	5,853	Illiterate	**20.5%**		
						Primary	**26.6%**		
						Middle	**19.0%**		
						Secondary	**14.0%**		
						Higher Secondary	**14.7%**		

				College and above	**11.9%**
	**Education Maharashtra**	**Alcohol use** any in last 12 months	6,091	Illiterate	**19.9%**
				Primary	**18.9%**
				Middle	**12.6%**
				Secondary	**11.3%**
				Higher Secondary	**11.8%**
				College and above	**12.0%**
	**Education Mizoram**	**Alcohol use** any in last 12 months	4,495	Illiterate	**14.8%**
				Primary	**8.2%**
				Middle	**1.3%**
				Secondary	**12.2%**
				Higher Secondary	**10.8%**
				College and above	**9.4%**
	**Education Kerala**	**Alcohol use** any in last 12 months	4,859	Illiterate	**14.5%**
				Primary	**21.6%**
				Middle	**23.4%**
				Secondary	**19.7%**
				Higher Secondary	**11.2%**
				College and above	**13.8%**
	**Education Tamil Nadu**	**Alcohol use** any in last 12 months	5,105	Illiterate	**15.3%**
				Primary	**19.9%**
				Middle	**17.7%**
				Secondary	**14.8%**
				Higher Secondary	**7.0%**
				College and above	**11.2%**
	**Education Uttarakhand**	**Alcohol use** any in last 12 months	5,443	Illiterate	**10.9%**
				Primary	**20.2%**
				Middle	**17.5%**
				Secondary	**19.6%**
				Higher Secondary	**13.9%**
				College and above	**18.1%**
				No adjustment described	

Peer-reviewed

Bonu et al. (2005) - Source of funding not reported

cross-sectional 22,685 India	high	Hospitalised participants of the 1995/6 Indian National Sample Survey. Aged >10 years	**Current regular alcohol use**	**Poverty**		Non-user	**1**	OR	
				borrowing/financial distress during hospitalisation		Alcohol user	**1.1**	0.64-1.86	>0.05
		adjusted for head of household, age, sex, level of schooling, marital status, state. number of days of hospitalisation used to control for severity of illness							
**Kar et al. (2010)** - Source of funding not reported									
cross-sectional 400 India	moderate	Residents at eight sites in Northern India. Aged >30 years	**Literacy**	**Current alcohol intake**	226	Literate	**0.9**	0.4-2.1	0.01
					177	Illiterate	**1**	OR	
						adjusted for age, sex, literacy, place of residence			
**Samuel et al. (2012)** - Funded by the British Heart Foundation									
cross-sectional 2,218 India	high	Young adults from population-based birth cohort in rural and urban areas of southern India. Aged 26-32 years	**Wealth (asset score)**	**Alcohol use**		Low (quintile 1)	**1**	OR	
						Middle-low (quintile 2)	**0.8**	0.6-1.3	
						Middle (quintile 3)	**0.8**	0.6-1.3	
						Middle-high (quintile l4)	**1**	0.7-1.5	
						High (Quintile 5)	**0.8**	0.5-1.3	
			**Education**	**Alcohol use**		Low (0 years formal schooling	**1**	OR	
						Middle-low (1-8 years)	**1.3**	0.7-2.3	
						Middle-high (9-12)	**1.1**	0.6-1.9	
						High (>12 years)	**0.6**	0.3-1.2	
			adjusted for gender, place of residence, possessions score, adult educational status, paternal educational status						
**Gupta et al. (2012)** - Funded by the South Asian Society of Atherosclerosis and Thrombosis									
cross-sectional 6,198 India	high	General population in middle-class areas of 11 cities, excluded house-bound, pregnant and those likely to die within 6 months. Aged 18-75 years	**Education**	**Alcohol abuse**	1,248	Low (0-10 years)	**9.6%**		
					2,956	Middle (11-15 years)	**10.3%**		
					1,366	High (>15 years)	**11.5%**		
			**Occupational Class (British Social Register, housewife = husband)**	**Alcohol abuse**	1,287	Low (4-5)	**5.1%**		
					1,677	Middle (3 manual/non-manua	**13.3%**		
					3,018	High (1-2)	**10.8%**		
			**SES (self-assessed)**	**Alcohol abuse**	374	Low (score 1-3)	**11.2%**		
					3,622	Middle (score 4-6)	**11.6%**		
					1,114	High (score 7-10)	**11.4%**		
						adjusted for age and sex			

Alcohol

Deepa et al. (2011) - Source of funding not reported

cohort	1,122	moderate	Urban residential colonies of	**Income**						
			Chennai. Aged >20y		**Alcohol use**	526	Middle (INR 5,501-10,000/mo	**4%**		<0.05
India					drinks any quantity of alcohol da	596	Low (INR 2,500-5,500/month)	**19%**		
							No adjustment described			

Kinra et al. (2010) - Funded by the Wellcome Trust

cross-sectional	high	Nationally representative sample of							
1,983		rural inhabitants from 1600 villages							
		in 18 states.	**SES (men) assets, housing**						
				**Alcohol use**	147	Low (asset score)	**33.7%**	26.2-41.2	<0.001
India		Aged 20-69 years		consumed >10 days/month any	358	Middle (asset score)	**26.9%**	22.3-31.5	
					870	High (asset score)	**20.2%**	17.5-22.9	
			**SES (women) assets, housing**	**Alcohol use**	106	Low (asset score)	**11.2%**	5.6-16.8	<0.001
				consumed >10 days/month any	143	Middle (asset score)	**8.1%**	3.4-12.9	
					359	High (asset score)	**2.5%**	1-4.1	
						adjusted for age			

Menon et al. (2015) - Source of funding not reported

cross-sectional	moderate	Representative sample from Kerala	**Poverty***					
84,456		state.		**Alcohol user**	62,975	Above state poverty line	**10.3%**	
India		Aged >18 years			21,481	Below state poverty line	**14.6%**	
			**Poverty***	**<1 drink/week**	62,975	Above state poverty line	**96.4%**	<0.0001
				**1 drink/week**			**1.2%**	
				**2 drinks/week**			**0.9%**	
				**3 drinks/week**			**0.6%**	
				**4 drinks/week**			**0.1%**	
				**5 drinks/week**			**0.1%**	
				**6 drinks/week**			**0.1%**	
				**7 drinks/week**			**0.5%**	
			**Poverty***	**<1 drink/week**	21,481	Below state poverty line	**94.3%**	<0.0001
				**1 drink/week**			**1.8%**	
				**2 drinks/week**			**1.4%**	
				**3 drinks/week**			**1.0%**	
				**4 drinks/week**			**0.3%**	
				**5 drinks/week**			**0.2%**	
				**6 drinks/week**			**0.1%**	
				**7 drinks/week**			**0.8%**	

**Keralan government definition: families which meet >4 of the following: no land or less than 5 cents, no house/dilapidated house; no sanitation latrine; no regular*

employed person in house; no access to safe drinking water; women-headed household or presence of widow or divorce; scheduled class/tribe; mentally retarded or (rural) disabled member in the family; no colour TV (urban) or family with an illiterate adult member								
						no adjustment described		
**Zaman et al. (2012)** - Funded by the Byrraju Foundation and the Initiative for Cardiovascular Health Research in Developing Countries								
cross-sectional	moderate	Representative sample from	**Education (men)**	**Alcohol use**	1,311	High (primary or higher)	**25.5%**	<0.001
4,535		20 villages in rural Andhra Pradesh.						
				≥1 drink on ≥1 day/week	895	Low (no formal education)	**36.8%**	
India		Aged >30 years						
			**Education (women)**	**Alcohol use**	1,074	High (primary or higher)	**0.5%**	0.014
				≥1 drink on ≥1 day/week	1,255	Low (no formal education)	**1.8%**	
			**Occupation (men)**	**Alcohol use**	434	Skilled*	**23.7%**	<0.001
				≥1 drink on ≥1 day/week	1,501	Unskilled	**34.8%**	
			**Occupation (women)**	**Alcohol use**	88	Skilled*	**2.3%**	0.997
				≥1 drink on ≥1 day/week	872	Unskilled	**2.3%**	
				**skilled manual labour, owner of business/farmer office worker/non-professional or professional)*				
			**Income (men)**	**Alcohol use**	912	High (>2000 INR/month)	**26.3%**	0.005
				≥1 drink on ≥1 day/week	717	Middle (1200-1999 INR/montl	**34.3%**	
					577	Low (0-1199 INR/month)	**30.7%**	
			**Income (women)**	**Alcohol use**	945	High (>2000 INR/month)	**1.4%**	0.35
				≥1 drink on ≥1 day/week	550	Middle (1200-1999 INR/montl	**0.9%**	
					834	Low (0-1199 INR/month)	**1.1%**	
						no adjustment described		
**Pillai et al. (2013)** - Funded by the Indian National Institute on Alcohol Abuse and Alcoholism, Public Health Institute								
cross-sectional	moderate	Urban and rural male	**Wealth (assets)**	**Drunkenness**		high (upper three quintiles)	**6.7%**	0.205
742		drinkers from Goa.		feel drunk at least once per				
				week		Low (lower two quintiles)	**7.8%**	
India		Aged 18-49 years						
			**Wealth (assets)**	**Frequent heavy drinking**		high (upper three quintiles)	**25.5%**	0.002
				>5 drinks in a single occasion at				
				least once a month		Low (lower two quintiles)	**35.8%**	
			**Wealth (assets)**	**High risk alcohol use**		high (upper three quintiles)	**14.5%**	0.002

				>60g pure alcohol consumed per drinking day in the last 12 months		Low (lower two quintiles)	**15.4%**		
			**Education**	**Drunkenness**		high (high school)	**5.2%**		0.205
				feel drunk at least once per week		middle (primary/middle schoc	**11.7%**		
						low (no education)	**16.2%**		
			**Education**	**Frequent heavy drinking**		high (high school)	**22.0%**		0.002
				>5 drinks in a single occasion at least once a month - i.e. binge		middle (primary/middle schoc	**44.5%**		
						low (no education)	**63.3%**		
			**Education**	**High risk alcohol use**		high (high school)	**64.0%**		0.002
				>60g pure alcohol consumed per drinking day in the last 12 months		middle (primary/middle schoc	**39.0%**		
						low (no education)	**8.0%**		
						no adjustment described			
**<u>Subramanian et al. (2005)</u>** - Source of funding not reported									
cross-sectional	high	Data from the nationally	**Caste (men)**	**Drink alcohol**	60,001	Other (High)	**1**	OR	
				household member drinks					
301,984		representative 1998/9		alcohol	41,984	Other backward class (Mediur	**1.08**	1.04-1.12	
India		Indian National Family			24,503	Scheduled caste (Low)	**1.43**	1.37-1.49	
		Health Survey.			18,362	Scheduled tribe (Low)	**2.04**	1.92-2.17	
		Aged >18 years							
			**Education (men)**	**Drink alcohol**	4,328	Highest (postgraduate)	**1**	OR	
				household member drinks					
				alcohol	13,004	High (college)	**1.11**	1.01-1.22	
					14,170	Medium-high (higher seconda	**1.21**	1.1-1.33	
					54,516	Medium-low (secondary)	**1.75**	1.61-1.91	
					27,504	Low (primary)	**2.13**	1.95-2.33	
					38,523	Lowest (Illiterate)	**2.28**	2.08-2.5	
			**Wealth (men) assets**	**Drink alcohol**	34,133	Highest	**1**	OR	
				household member drinks					
				alcohol	31,611	High	**1.12**	1.07-1.18	
					30,413	Medium	**1.30**	1.24-1.37	

					28,985	Low	**1.59**	1.51-1.68	
					26,903	Lowest	**1.92**	1.81-2.03	
			**Caste (women)**	**Drink alcohol** household member drinks alcohol	58,977	Other (High)	**1**	OR	
					41,614	Other backward class (Mediur	**1.55**	1.31-1.83	
					23,847	Scheduled caste (Low)	**1.66**	1.47-1.87	
					18,373	Scheduled tribe (Low)	**3.74**	2.79-5	
			**Education (women)**	**Drink alcohol** household member drinks alcohol	2,154	Highest (postgraduate)	**1**	OR	
					6,948	High (college)	**0.85**	0.72-1	
					7,571	Medium-high (higher seconda	**0.55**	0.41-0.75	
					33,133	Medium-low (secondary)	**0.81**	0.62-1.05	
					22,952	Low (primary)	**1.00**	0.74-1.35	
					77,181	Lowest (Illiterate)	**1.31**	1-1.71	
			**Wealth (women) assets**	**Drink alcohol** household member drinks alcohol	34,049	Highest	**1**	OR	
					30,798	High	**1.17**	0.94-1.45	
					29,156	Medium	**1.60**	1.23-2.09	
					27,836	Low	**1.96**	1.53-2.43	
					28,100	Lowest	**2.72**	2.18-3.39	

adjusted for living environment, marital status, age, religion, caste, education, household standard of living index

Hashibe et al. (2003) - Funded by Assoc. Int Cancer Research; Imperial Cancer Research Fund; National Cancer Institute (USA) & UCLA Jonnson Cancer Centre Foundation

case-control 47,773 India	high	Study examining SES and premalignant oral lesions in Kerala. Data is presented linking NCD risk factors with SES markers for the 47,773 controls only. Aged >35 years	**Income**	**Drinking**	Low (INR<1500	**16.8%**	
					Middle-low (INR 1500-3000)	**12.4%**	
					Middle-high (INR 3001-5000)	**11.2%**	
					High (INR>5000)	**9.3%**	<0.0001
			**Education**	**Drinking**	None/illiterate	**11.6%**	
					None/literate	**8.3%**	
					Primary	**15.4%**	
					Middle	**17.2%**	
					>High school	**15.4%**	<0.0001
			**Occupation**	**Drinking**	Manual	**12.5%**	
					Teacher/office	**18.7%**	
					Business	**30.0%**	
					Retired	**24.7%**	
					Other	**34.3%**	<0.0001

No adjustment described

Neufeld et al. (2005) - No external funding

cross-sectional	high	Data from the 1995/6 Indian	**Poverty***	**Alcohol**	303416	High (above poverty line)	**1**	OR	
471,143		National Sample Survey. Aged >10		regular use of any alcoholic					
		years		beverage	167,727	Low (below poverty line)	**1.2**	1.1-1.4	
India									
			**Caste****	**Alcohol**	334,512	High	**1**	OR	
				regular use of any alcoholic					
				beverage	136,631	Low	**3.4**	3-3.8	
			**Education**	**Alcohol**	273,069	High (formal education)	**1**	OR	
				regular use of any alcoholic					
				beverage	188,956	Low (no formal education)	**1.3**	1.2-1.4	

**Planning commission of India definition - income required to ensure adequate intake of calories (INR 2,100 urban; INR 2,400 rural)*
***Scheduled Castes and tribes - identified in the Indian Constitution as especially disadvantaged or needy*

adjusted for age group, gender caste, income, residence, education

Dhungana et al. (2014) - Source of funding not reported

cross-sectional	high	Rural community in the Sindhuli	**Caste**						
406		district.		**Alcohol use**		High (Brahman/Chhetri)	**31.1%**		<0.001
Nepal		Aged 20-50 years		use of alcohol until up to 30					
				days before interview		Middle (Adhibasi/Janajati)	**56.2%**		
						Low (Dalits)	**42.9%**		
			**Education**	**Alcohol use**		Low (No formal education)	**58.5%**		<0.001
				use of alcohol until up to 30					
				days before interview		Middle-low (lower than prima	**55.2%**		
						Middle-high (primary)	**36.1%**		
						High (Secondary and higher)	**26.0%**		
			**SES (education, occupation,**						
			**income)**	**Alcohol use**		Lowest	**57.4%**		0.012
				use of alcohol until up to 30					
				days before interview		Middle-low	**42.6%**		
						Middle-high	**53.1%**		
						Highest	**31.2%**		

No adjustment described

Rahlenbeck and Gebre-Yohannes (1998) - Funded by the Ethiopian Science and Technology Commission resources

cross-sectional 66	low	Medical students at Gondar college							
		in north-west Ethiopia.							
		Age range not reported (mean 20.8	**Income (parental)**						
		years, SD=1.6)							
				**Alcohol use**	17	High (>US$150/month)	**35%**		0.013

Ethiopia					49	Low (<US$150/month)	**31%**		
						No adjustment described			
**Taylor et al (1996)** - Funded by the International Network for Clinical Epidemiology; National Institute of Medical Research (Lagos)									
cross-sectional	low	Survey of adults selected	**Income (men) 20-39 years**	**Alcohol consumption**		Low (<NGN 3000/year)	**75.2%**		<0.01
882		from the civil service, three research							
		institutions and two communities.							
				'consumes alcohol'		High (>NGN 3000/year)	**79.5%**		
Nigeria		Aged >20 years							
			**Income (men) 40-59 years**	**Alcohol consumption**		Low (<NGN 3000/year)	**63.5%**		<0.01
				'consumes alcohol'		High (>NGN 3000/year)	**90.0%**		
			**Income (women) 20-39 years**	**Alcohol consumption**		Low (<NGN 3000/year)	**38.1%**		<0.01
				'consumes alcohol'		High (>NGN 3000/year)	**68.8%**		
			**Income (women) 40-59 years**	**Alcohol consumption**		Low (<NGN 3000/year)	**30.6%**		<0.01
				'consumes alcohol'		High (>NGN 3000/year)	**76.3%**		
						No adjustment described			
**Bunker et al. (1996)** - Funded by the National Institute of Health									
cross-sectional	moderate	Civil servants selected from	**SES (seniority) women**	**Alcohol intake**	157	Senior staff	**29.5g**		>0.05
713		3 different sites at three		mean ethanol intake g/week	234	Junior staff	**26.6g**		
Nigeria		time periods: 1988, 1990 and							
		1992. This data from the	**SES (seniority) men**	**Alcohol intake**	401	Senior staff	**145.6g**		>0.05
		1992 Benin wave.		mean ethanol intake g/week	635	Junior staff	**161.4g**		
		Aged 25-54 years							
						No adjustment described			
**Sossa et al. (2013)** - Funded by the Canadian International Development Agency									
cohort 208	moderate	Healthy adults sampled from a large							
		city, small town and rural area.	**Education**						
				**Alcohol intake**		High school	**8.2g**	(SD) 13.9	
Benin		Aged 25-60 years		grams ethanol/day		Primary schooling	**5.1g**	(SD) 8.6	
						No Schooling	**3.2g**	(SD) 7.1	<0.01
						No adjustment described			
			**Wealth (assets)**	**Alcohol intake**		High	**6.8g**	(SD) 13.4	
				grams ethanol/day		Medium	**5.4g**	(SD) 8.8	
						Low	**5.6g**	(SD) 10.3	0.5
						No adjustment described			
			**Income (US$/dependent/day)**	**Ever drinker**		Extremely Low (<$1/day)	**1**	OR	
				ever drank alcohol		Low ($1-2/day)	**1.16**	0.79-1.69	
						Higher ($>2/day)	**1.2**	0.72-1.99	

						adjusted for age, sex, living history			
**<u>Houehanou et al. (2015)</u>** - No external funding									
cross-sectional<br>5,830	moderate	Population-based nationally representative 2008 STEPS survey. Aged 25-64 years	**Income**						
				**Alcohol use**	3,549	Lowest (<FCFA 66,000*)	**17.8%**		0.003
Benin				consume alcohol ≥4 days/week	1,034	Low (FCFA 66,000-132,000)	**16.5%**		
					565	Middle (FCFA 132,000-198,00(	**15.9%**		
					378	High (FCFA 198,000-264,000)	**12.4%**		
				**€100 = FCFA 65,657 at time of s*	304	Highest (>FCFA 264,000)	**11.2%**		
						No adjustment described			
**<u>Cubbins et al. (2012)</u>** - Funded by the National Institute on Alcohol Abuse and Alcoholism (USA)									
RCT<br>5,543<br>Zimbabwe	low	Baseline data from this trial of a behavioural intervention to reduce harmful alcohol use and risky sex at 30 rural sites. Aged 18-30 years Logistic regression				Logistic regression coefficient			
			**No. months employed last yr**	**Current alcohol use**<br>drank alcohol at least once in last 30 days			**0.16**	(SE) 0.008	>0.05
			**No. months employed last yr**	**Frequency of alcohol use**<br>number of days drank in last 30 days			**0.37**	(SE) 0.016	<0.05
			**No. months employed last yr**	**Quantity of drinks consumed**<br>number of drinks consumed on drinking days			**0.29**	(SE) 0.007	<0.001
			**No. months employed last yr**	**Frequency of getting drunk**<br>frequency of drunkenness in last 30 days			**0.28**	(SE) 0.011	<0.01
			**Years of education**	**Current alcohol use**<br>drank alcohol at least once in last 30 days			**-0.1**	(SE) 0.015	>0.05
			**Years of education**	**Frequency of alcohol use**<br>number of days drank in last 30 days			**0.052**	(SE) 0.02	<0.01
			**Years of education**	**Quantity of drinks consumed**<br>number of drinks consumed on drinking days			**0.33**	(SE) 0.012	<0.01
			**Years of education**	**Frequency of getting drunk**			**0.039**	(SE) 0.015	<0.05
adjusted for age, sex, months living away from home last year, ethnicity, religion, marital status, parity, years of education, months of employment last year, province crude death rate province population density, wave of study									
**<u>Laux (2014)</u>** -Funded by National Institutes of Health (USA); University of Pittsburgh School of Medicine; National Centre for Research (USA)									
cross-sectional<br>1,355	moderate	Six communities in central and western Nicaragua. Aged 20-60 years	**Income (US$/dependent/day)**						
				**Ever drinker**		Extremely Low (<$1/day)	**1**	OR	

	ever drank alcohol	Low ($1-2/day)	**1.16**	0.79-1.69
		Higher ($>2/day)	**1.2**	0.72-1.99
		adjusted for age, sex, living history		

Design, n	Quality	Sample	Exposure	Outcome	n	Exposure subgroup	Value	95% CI	p
Non peer-reviewed									
**WHO STEPS Togo (2010)** - Source of funding not reported									
cross-sectional 4,370	moderate	Nationally representative STEPS survey. Residents from 6 zones who have lived there for >6 months. Aged 15-64 years	**Education**	**Harmful alcohol use** ≥60 g of pure alcohol/day for men, ≥40g/day for women	939	No formal education	**4.3%**	2.7-5.9	
					503	Primary	**2.3%**	0.8-3.8	
Togo					430	Secondary	**3.3%**	1.3-5.4	
					276	College or equivalent	**2.9%**	0.8-5.1	
					73	High school	**3.2%**	0-7.2	
					55	University	**0.0%**	0-0	
			**Education**	**Insufficient fruit and veg**	1,761	No formal education	**96.4%**	95-97.7	
					914	Primary	**94.8%**	92.8-96.8	
					875	Secondary	**93.8%**	91.5-96.2	
					504	College or equivalent	**93.0%**	90.4-95.6	
					133	High school	**96.1%**	93-99.3	
					106	University	**95.3%**	91.3-99.3	
						no adjustment described			
**WHO STEPS Eritrea (2004)** - Funded by WHO, Italian Government, Eritrean Government									
cross-sectional 2,033	moderate	Nationally representative sample, individuals selected from six zones. Aged 15-64 years	**Education**	**Low vegetable intake** <2 servings per day	813	Lowest (no formal education)	**54.2%**		0.016
					721	Low (Grades 1-6)	**50.8%**		
Eritrea					286	Middle (Grade 7-8)	**47.2%**		
					438	High (Grades 9-12)	**48.4%**		
					103	Highest (>12 years)	**38.8%**		
					2,352 total				
						no adjustment described			
**WHO STEPS India (2007)** - Funded by World Bank, Indian Government									
cross-sectional 38,064	moderate	WHO STEPS survey in seven states. Aged 15-64	**Education Andhra Pradesh**	**Low fruit and vegetables** <5 servings /day	6,218	Illiterate	**92.8%**		
						Primary	**88.7%**		
						Middle	**85.5%**		
India						Secondary	**84.0%**		
						Higher Secondary	**83.3%**		

			College and above	**81.5%**
**Education Madhya Pradesh**	**Low fruit and vegetables** <5 servings /day	5,853	Illiterate	**86.9%**
			Primary	**84.0%**
			Middle	**85.2%**
			Secondary	**83.8%**
			Higher Secondary	**69.3%**
			College and above	**65.9%**
**Education Maharashtra**	**Low fruit and vegetables** <5 servings /day	6,091	Illiterate	**81.2%**
			Primary	**80.9%**
			Middle	**77.1%**
			Secondary	**73.1%**
			Higher Secondary	**73.0%**
			College and above	**69.6%**
**Education Mizoram**	**Low fruit and vegetables** <5 servings /day	4,495	Illiterate	**91.5%**
			Primary	**84.5%**
			Middle	**85.8%**
			Secondary	**84.1%**
			Higher Secondary	**81.1%**
			College and above	**81.4%**
**Education Kerala**	**Low fruit and vegetables** <5 servings /day	4,859	Illiterate	**94.1%**
			Primary	**94.9%**
			Middle	**92.2%**
			Secondary	**88.8%**
			Higher Secondary	**84.5%**
			College and above	**78.2%**
**Education Tamil Nadu**	**Low fruit and vegetables** <5 servings /day	5,105	Illiterate	**99.5%**
			Primary	**99.5%**
			Middle	**99.1%**
			Secondary	**98.6%**
			Higher Secondary	**98.5%**
			College and above	**97.2%**
**Education Uttarakhand**	**Low fruit and vegetables** <5 servings /day	5,443	Illiterate	**94.6%**
			Primary	**93.1%**
			Middle	**90.4%**
			Secondary	**86.3%**

					Higher Secondary	**83.0%**	
					College and above	**80.5%**	
					no adjustment described		

Hashibe et al. (2003) - Funded by Assoc. Int Cancer Research; Imperial Cancer Research Fund; National Cancer Institute (USA) & UCLA Jonnson Cancer Centre Foundation

case-control<br>47,773<br>India	high	Study examining SES and premalignant oral lesions in premalignant oral lesions in Kerala. Data is presented linking NCD risk factors with SES markers for the 47,773 controls only.<br>Aged >35 years	**Income**	**Fruits - high intake**	Low (INR<1500	**87.5%**	
					Middle-low (INR 1500-3000)	**92.1%**	
					Middle-high (INR 3001-5000)	**93.0%**	
					High (INR>5000)	**93.9%**	<0.0001
			**Education**	**Fruits - high intake**	Low (None/illiterate)	**79.4%**	
					Middle-low (None/literate)	**94.0%**	
					Middle (Primary)	**89.4%**	
					Middle-high (Middle school)	**90.9%**	
					High (>High schools)	**93.5%**	<0.0001
			**Occupation**	**Fruits- high intake**	Manual	**89.4%**	
					Teacher/office	**95.2%**	
					Business	**90.8%**	
					Retired	**86.1%**	
					Other	**94.9%**	<0.0001
			**Income**	**Daily vegetables**	Low (INR<1500	**88.6%**	
					Middle-low (INR 1500-3000)	**96.2%**	
					Middle-high (INR 3001-5000)	**97.5%**	
					High (INR>5000)	**98.5%**	<0.0001
			**Education**	**Daily vegetables**	None/illiterate	**80.6%**	
					None/literate	**92.4%**	
					Primary	**91.6%**	
					Middle	**94.1%**	
					>High schools	**97.1%**	<0.0001
			**Occupation**	**Daily vegetables**	Manual	**91.3%**	
					Teacher/office	**98.7%**	
					Business	**95.6%**	
					Retired	**94.6%**	
					Other	**96.8%**	<0.0001
					no adjustment described		

Singh et al. (1998) - Funding source not reported

cross-sectional	moderate	Representative sample of	**Income (males)**	**High saturated fat intake**	High (>600 rupees/month)	**41%**	

1,806<br>India		urban residents of Moradabad city.<br>Aged 25-64 years		saturated fat comprises >10% kcal per day		Medium (300-600 rupees/month)	**51%**	
						Low (<300 rupees/month)	**7%**	
			**Income (women)**	**High saturated fat intake**<br>saturated fat comprises >10% kcal per day		High (>600 rupees/month)	**43%**	
						Medium (300-600 rupees/month)	**38%**	
						Low (<300 rupees/month)	**19%**	
			**Education (men)**	**High saturated fat intake**<br>saturated fat comprises >10% kcal per day		High (>5 years)	**53%**	
						Medium (primary only)	**39%**	
						Low (no education)	**7%**	
			**Education (women)**	**High saturated fat intake**<br>saturated fat comprises >10% kcal per day		High (>5 years)	**54%**	
						Medium (primary only)	**33%**	
						Low (no education)	**13%**	
						no adjustment described		

Menon et al. (2015) - Source of funding not reported

cross-sectional<br>84,456<br>India	moderate	Representative sample from Kerala state.<br>Aged >18 years	**Poverty***	**0 non-vegetarian days/week**	62,975	Above state poverty line	**83.6%**	<0.0001
				**1 non-vegetarian day/week**			**16.4%**	
				**2 non-vegetarian days/week**			**0.1%**	
				**3 non-vegetarian days/week**			**0.0%**	
				**4 non-vegetarian days/week**			**0.0%**	
				**5 non-vegetarian days/week**			**0.0%**	
				**6 non-vegetarian days/week**			**0.0%**	
				**7 non-vegetarian days/week**			**0.2%**	
			**Poverty***	**0 non-vegetarian days/week**	21,481	Below state poverty line	**86.7%**	<0.0001
				**1 non-vegetarian day/week**			**12.8%**	
				**2 non-vegetarian days/week**			**0.1%**	
				**3 non-vegetarian days/week**			**0.0%**	
				**4 non-vegetarian days/week**			**0.0%**	
				**5 non-vegetarian days/week**			**0.0%**	
				**6 non-vegetarian days/week**			**0.0%**	
				**7 non-vegetarian days/week**			**0.3%**	
			**Poverty***	**Use oil on <1 day/week**	62,975	Above state poverty line	**10.1%**	<0.0001
				**Use oil 1 day/week**			**89.3%**	
				**Use oil 2 days/week**			**0.4%**	

				**Use oil 3 days/week**			**0.1%**		
				**Use oil 4 days/week**			**0.0%**		
				**Use oil 5 days/week**			**0.1%**		
				**Use oil 6 days/week**			**0.0%**		
				**Use oil 7 days/week**			**0.1%**		
			**Poverty***	**Use oil on <1 day/week**	21,481	Below state poverty line	**7.6%**		<0.0001
				**Use oil 1 day/week**			**92.1%**		
				**Use oil 2 days/week**			**0.2%**		
				**Use oil 3 days/week**			**0%**		
				**Use oil 4 days/week**			**0%**		
				**Use oil 5 days/week**			**0%**		
				**Use oil 6 days/week**			**0%**		
				**Use oil 7 days/week**			**0%**		
				**Keralan government definition: families which meet >4 of the following: no land or less than 5 cents, no house/dilapidated house; no sanitation latrine; no regular employed person in house; no access to safe drinking water; women-headed household or presence of widow or divorce; scheduled class/tribe; mentally retarded or (rural) disabled member in the family; no colour TV (urban) or family with an illiterate adult member*					
						no adjustment described			
**Singh et al. (2000)** - Sandoz (Novartis) Foundation of Gerontologic Research (AUS); World Health Federation									
cross-sectional<br>3,257<br>India	low	Representative female sample from 5 cities. Aged 25-64 years	**SES (education, occupation, income, assets,**	**High salt intake**<br>>6g/day	985	High	**50.3%**		<0.09
					790	Medium-high	**53.3%**		
					774	Medium	**66.9%**		
					602	Medium-low	**66.1%**		
					206	Low	**59.7%**		
			**SES (education, occupation, income, assets,**	**Sugar and confectionary**<br>average g/day	985	High	**52g**	(SD) 71	<0.05
					790	Medium-high	**42g**	(SD) 12	
					774	Medium	**32g**	(SD) 15	

					602	Medium-low	**25g**	(SD) 8	
					206	Low	**13g**	(SD) 3	
			**SES (education, occupation, income, assets,**	**Total visible fat** average g/day	985	High	**45g**	(SD) 18	<0.01
					790	Medium-high	**34g**	(SD) 15	
					774	Medium	**27g**	(SD) 12	
					602	Medium-low	**22g**	(SD) 9	
					206	Low	**8g**	(SD) 4	
			**SES (education, occupation, income, assets,**	**Fruits, vegetables & legumes** average g/day	985	High	**225g**	(SD) 55	>0.05
					790	Medium-high	**222g**	(SD) 51	
					774	Medium	**256g**	(SD) 38	
					602	Medium-low	**210g**	(SD) 32	
					206	Low	**140g**	(SD) 26	
			**SES (education, occupation, income, assets,**	**Fruits, veg & legumes : fat** ratio	985	High	**5**	(SD) 2	<0.05
					790	Medium-high	**6.5**	(SD) 2	
					774	Medium	**9.4**	(SD) 3	
					602	Medium-low	**9.5**	(SD) 4	
					206	Low	**17.5**	(SD) 5	
						no adjustment described			
**Zaman et al. (2012)** - Funded by the Byrraju Foundation and the Initiative for Cardiovascular Health Research in Developing Countries									
cross-sectional 4,535 India	moderate	Representative sample from 20 villages in rural Andhra Pradesh. Aged >30 years	**Education (men)**	**Low fruit intake** fruit consumed ≤1 days/week	1,311	High (primary or higher)	**37.4%**		<0.001
					895	Low (no formal education)	**55.6%**		
			**Education (women)**	**Low fruit intake** fruit consumed ≤1 days/week	1,074	High (primary or higher)	**43.4%**		<0.001
					1,255	Low (no formal education)	**56.7%**		
			**Occupation (men)**	**Low fruit intake** fruit consumed ≤1 days/week	434	Skilled*	**31.1%**		<0.001
					1,501	Unskilled	**47.8%**		
			**Occupation**	**Low fruit intake**	88	Skilled*	**50.0%**		0.344

				fruit consumed ≤1 days/week	872	Unskilled	**55.4%**	
				**skilled manual labour, owner of business/farmer office worker/non-professional or professional)*				
			**Income (men)**	**Low fruit intake**	912	High (>2000 INR/month)	**36.0%**	<0.001
				fruit consumed ≤1 days/week	717	Middle (1200-1999 INR/month)	**48.5%**	
					577	Low (0-1199 INR/month)	**54.8%**	
			**Income (women)**	**Low fruit intake**	945	High (>2000 INR/month)	**43.4%**	<0.001
				fruit consumed ≤1 days/week	550	Middle (1200-1999 INR/month)	**49.8%**	
					834	Low (0-1199 INR/month)	**59.2%**	
						no adjustment described		

<u>Gupta et al. (2012)</u> - Funded by the South Asian Society of Atherosclerosis and Thrombosis

cross-sectional	high	General population in middle-class areas of 11	**Education**	**High dietary fat**	1,248	Low (0-10 years)	**46.2%**	
6,198				>20g/day	2,956	Middle (11-15 years)	**49.8%**	
India		cities, excluded house-			1,366	High (>15 years)	**60.0%**	
		bound, pregnant and those						
		likely to die within 6						
			**Occupational Class (British Social housewife =**	**High dietary fat**	1,287	Low (4-5)	**40.6%**	
		months.		>20g/day	1,677	Middle (3 manual/non-manual)	**49.7%**	
		Aged 18-75 years			3,018	High (1-2)	**55.7%**	
			**SES (self-assessed)**	**High dietary fat**	374	Low (score 1-3)	**41.8%**	
				>20g/day	3,622	Middle (score 4-6)	**53.1%**	
					1,114	High (score 7-10)	**61.6%**	
			**Education**	**Low fruit and vegetables**	1,248	Low (0-10 years)	**23.9%**	
				<2 servings/day	2,956	Middle (11-15 years)	**29.3%**	
					1,366	High (>15 years)	**28.9%**	
			**Occupational Class (British Social housewife =**	**Low fruit and vegetables**	1,287	Low (4-5)	**26.9%**	
				<2 servings/day	1,677	Middle (3 manual/non-manual)	**26.8%**	
					3,018	High (1-2)	**26.4%**	

			**SES (self-assessed)**	**Low fruit and vegetables** <2 servings/day	374	Low (score 1-3)	**47.8%**	
					3,622	Middle (score 4-6)	**24.7%**	
					1,114	High (score 7-10)	**21.8%**	
						adjusted for age and sex		
**Agrawal et al. (2014a)** - Funded by a Wellcome Trust Strategic Award Grant								
cross-sectional 156,317 India	high	Nationally representative 2005/6 National Family Health Survey, participants aged 20-49 years	**Caste**	**Non-vegetarian** eats meat, fish, milk, eggs, curd, dairy products	29,831	Scheduled caste (poorest)	**74.0%**	<0.001
					12,734	Scheduled tribe (poorest)	**75.2%**	
					60,977	Other backward class (poor)	**60.4%**	
					48,854	General (more affluent)	**57.7%**	
			**SES (assets and**	**Non-vegetarian** eats meat, fish, milk, eggs, curd, dairy products	26,389	Lowest	**71.1%**	<0.001
					28,751	Low	**66.2%**	
					31,232	Middle	**65.4%**	
					33,560	High	**64.1%**	
					36,385	Highest	**54.0%**	
						no adjustment described		
**Agrawal et al. (2014b)** - Funded by the Wellcome Trust; Council for England; National Institute for Health Research Collaboration								
cross-sectional 156,317 India	high	Nationally representative 2005/6 National Family Health Survey, participants aged 20-49 years	**Caste**	**Daily fish consumption**		Low caste	**3.4%**	<0.001
						Other caste	**44.0%**	
			**Wealth (assets and housing)**	**Daily fish consumption**		Highest (top quintile)	**39.1%**	<0.001
						Lowest (bottom quintile)	**8.2%**	
						no adjustment described		
**Ganesan at al. (2012)** - No external funding								

cross-sectional 1,261	high	1,261 Diabetics and 122 non-diabetics from the population-based urban Sankara Nethralaya Diabetic Retinopathy Epidemiology and Molecular Genetic Study. Aged >40 years						
India			**SES (undefined)**	**Low fibre diet** fibre score <32 on validated questionnaire		Low	**11.9%**	0.002
						Middle	**71.9%**	0.237
						High	**16.1%**	<0.0001
			**SES (undefined)**	**High fibre diet** fibre score >32 on validated questionnaire		Low	**6.5%**	0.002
						Middle	**68.8%**	0.237
						High	**24.6%**	<0.0001
						no adjustment described		

Radhika et al. (2007) - Funded by the Chennai Willingdon Corporate Foundation

cross-sectional 1,902	moderate	Residents of Chennai recruited for CURES study on salt and hypertension. Excludes those with hypertension, diabetes and heart disease. Aged >20 years						
India			**Household Income** monthly	**Salt intake** mean grams/day		High (>INR10000)	**9.4g**	0.006
						Medium-high (INR5000-10000)	**9.0g**	
						Medium-low (INR2000-5000)	**8.5g**	
						Low (<INR2000)	**7.9g**	
						no adjustment described		

Mehta (2000) - Source of funding not reported

cross-sectional 320	low	Elderly Indian residents of Baroda city, selected from corporate sector, senior	**Income (men)**	**Fat intake** grams/day	60	High (undefined)	**305g**	>0.05
					60	Middle (undefined)	**303g**	
India					50	Low (undefined)	**126g**	

		citizens council, retired peoples club, vadil vahar, women's clubs, temple and slum pockets of Baroda city. Aged 60-70 years							
			**Income (women)**	**Fat intake**	50	High (undefined)	**161g**		>0.05
				grams/day	50	Middle (undefined)	**152g**		
					50	Low (undefined)	**99g**		
**Kinra et al. (2010)** - Funded by the Wellcome Trust									
cross-sectional 1,983 India	high	Nationally representative sample of rural inhabitants from 1600 villages in 18 states.	**SES (men) assets,**	**Low fruit and vegetable intake**	147	Low (asset score)	**81.0%**	74.5-87.5	<0.001
				<400g/day	358	Middle (asset score)	**75.6%**	71.2-79.9	
		Aged 20-69 years			870	High (asset score)	**63.6%**	60.3-66.8	
			**SES (women) assets, housing**	**Low fruit and vegetable intake**	106	Low (asset score)	**86.6%**	77.5-95.7	<0.001
				<400g/day	143	Middle (asset score)	**78.5%**	71.4-85.5	
					359	High (asset score)	**69.9%**	65.2-74.7	
				variables; age standardised					
			**SES (men) assets,**	**Low physical activity**	147	Low (asset score)	**65.2%**	57.5-72.9	0.11
				<1.69 MET	358	Middle (asset score)	**72.4%**	67.8-77	
					870	High (asset score)	**72.9%**	70-75.9	
			**SES (women) assets, housing**	**Low physical activity**	106	Low (asset score)	**66.0%**	54.5-77.4	0.084
				<1.69 MET	143	Middle (asset score)	**73.5%**	66.0-81.0	
					359	High (asset score)	**76.5%**	72-81	
						no adjustment described			
**Dewi et al. (2010)** - Funded by Provincial Health Office of Yogyakarta Special Regency, Indonesia									
cross-sectional 3,285	moderate	Representative sample of residents of Yogyakarta	**Income (male) 15-35 years**	**Low fruit & vegetable intake**		Low (<mean income	**85%**		
				<4.5 portions/day		High (> mean income)	**78%**		

Indonesia		Aged 15-75 years						
			**35-54 years**			Low (<mean income	**83%**	
						High (> mean income)	**79%**	
			**55-75 years**			Low (<mean income	**85%**	
						High (> mean income)	**76%**	
			**Income (female) 15-35 years**	**Low fruit & vegetable intake**		Low (<mean income	**84%**	
				<4.5 portions/day		High (> mean income)	**76%**	
			**35-54 years**			Low (<mean income	**84%**	
						High (> mean income)	**75%**	
			**55-75 years**			Low (<mean income	**83%**	
						High (> mean income)	**78%**	
						no adjustment described		
**Mumu et al. (2014)** - Source of funding not reported								
cross-sectional 374	moderate	Type 2 diabetics who had been diagnosed for >1 year from 9 health centres around Dhaka. Aged >20 years						
			**Education**	**Smoker (continues)**	112	Low (up to primary)	**6.3%**	0.541
Bangladesh				still smoking despite medical advice to quit	174	Medium (up to higher secondary)	**4.6%**	
					88	High (graduate and above)	**8.0%**	
			**Education**	**Unhealthy diet**	112	Low (up to primary)	**93.8%**	0.076
				non-adherence to recommended diet and quantity of food	174	Medium (up to higher secondary)	**85.1%**	
					88	High (graduate and above)	**86.4%**	
			**Education**	**Sedentary**	112	Low (up to primary)	**26.8%**	0.654
				<30 mins planned exercise/day	174	Medium (up to higher secondary)	**22.4%**	
					88	High (graduate and above)	**26.1%**	
						no adjustment described		
**Dhungana et al. (2014)** - Source of funding not reported								
cross-sectional 406 Nepal	high	Rural community in the Sindhuli district. Aged 20-50 years	**Caste**	**Low fruit and vegetables** <400g/day		High (Brahman/Chhetri)	**100.0%**	0.014
						Middle (Adhibasi/Janajati)	**94.5%**	
						Low (Dalits)	**100.0%**	

			**Education**	**Low fruit and vegetables**<br><400g/day		Low (No formal education)<br>Middle-low (lower than primary)<br>Middle-high (primary)<br>High (Secondary and higher)	**97.9%**<br>**93.1%**<br>**97.2%**<br>**95.5%**	0.32
			**SES (education, occupation, income)**	**Low fruit and vegetables**<br><400g/day		Lowest<br>Middle-low<br>Middle-high<br>Highest<br>no adjustment described	**96.7%**<br>**98.9%**<br>**96.9%**<br>**81.2%**	0.001
**Babalola et al. (2011)** - Source of funding not reported								
cross-sectional<br>192<br>Nigeria	moderate	Semi-urban community in Nigeria<br>Age not reported	**Years of education**	**Unhealthy diet**<br>more processed food, foods of animal origin, sugar and fat		Years	**0.8088***	<0.01
			**Average income**	**Unhealthy diet**<br>more processed food, foods of animal origin, sugar and fat		NGN	**0.1796***	<0.01
				*Standardised coefficient from logit regression for determinants of incidence of nutrition transition (unhealthy diet)				
**Bunker et al. (1996)** - Funded by the National Institute of Health								
cross-sectional<br>713<br>Nigeria	moderate	Civil servants selected from 3 different sites at three time periods: 1988, 1990 and 1992. This data from the 1992 Benin wave.<br>Aged 25-54 years	**SES (seniority)**	**Meat**<br>mean grams/day	113<br>163	Senior staff<br>Junior staff	**50.4g**<br>**33.3g**	<0.05
			**SES (seniority)**	**Fish**<br>mean grams/day	113<br>163	Senior staff<br>Junior staff	**54.5g**<br>**69.8g**	<0.05
			**SES (seniority)**	**Milk**<br>any intake in previous 24h	113<br>163	Senior staff<br>Junior staff	**34.4%**<br>**16.7%**	<0.05
			**SES (seniority)**	**Eggs**<br>any intake in previous 24h	113<br>163	Senior staff<br>Junior staff	**21.3%**<br>**15.4%**	>0.05
			**SES (seniority)**	**Carbohydrates**<br>% of total calories	113<br>163	Senior staff<br>Junior staff	**67.2%**<br>**66.6%**	>0.05

			**SES (seniority)**	**Fat**<br>% of total calories	113<br>163	Senior staff<br>Junior staff	**22.5%**<br>**23.1%**	>0.05
			**SES (seniority) men**	**Meat**<br>mean grams/day	401<br>635	Senior staff<br>Junior staff	**50.2g**<br>**40.2g**	>0.05
			**SES (seniority) men**	**Fish**<br>mean grams/day	401<br>635	Senior staff<br>Junior staff	**77.7g**<br>**78.7g**	>0.05
			**SES (seniority) men**	**Milk**<br>any intake in previous 24h	401<br>635	Senior staff<br>Junior staff	**9.50%**<br>**6.50%**	>0.05
			**SES (seniority) men**	**Eggs**<br>any intake in previous 24h	401<br>635	Senior staff<br>Junior staff	**16.80%**<br>**7.40%**	<0.05
			**SES (seniority) men**	**Carbohydrates**<br>% of total calories	401<br>635	Senior staff<br>Junior staff	**66.20%**<br>**69.10%**	<0.001
			**SES (seniority) men**	**Fat**<br>% of total calories	401<br>635	Senior staff<br>Junior staff<br>adjusted	**23%**<br>**20.50%**	<0.001

<u>**Delise et al. (2012)**</u> - Canadian Institutes of Health Research

cross-sectional<br>541<br>Benin	moderate	Adults in good health born in Benin, resident in one of three sites (urban, peri-urban and rural) for >6 months. Exclude those with hypertension, diabetes or heart disease (outcomes of this study).<br>Aged 25-60 years	**Education**	**Legumes/nuts**<br>intake*		Secondary or higher vs none	**0.035**	>0.05
			**Education**	**Fruits**<br>intake*		Secondary or higher vs none	**0.073**	>0.06
			**Education**	**Vegetables**<br>intake*<br><br>**taken from two non-consecutive 24h food recall aided by calibrated local utensils*		Secondary or higher vs none	**0.17**	<0.01
			**SES (education, household**	**Legumes/nuts**<br>intake*		High vs Low (tertiles)	**-0.061**	>0.05
			**SES (see above)**	**Fruits**		High vs Low (tertiles)	**0.043**	>0.05

				intake*					
			SES (see above)	**Vegetables** intake*		High vs Low (tertiles)	**0.052**		>0.05
				**taken from two non-consecutive 24h food recall aided by calibrated local utensils*					
						adjusted but variables not described			
**Nwamarah and Otitoju (2014)** - Source of funding not reported									
cross-sectional 170 Nigeria	low	Elderly and retired staff from the University of Nigeria, 30% below the poverty line. Aged >60 years	**Income**	**Fruit and vegetable consumption**	52	Below poverty line (US$435)	**4.21**	(SD) 2.31	0.02
				mean servings per week	118	Above poverty line (US$435)	**5.12**	(SD) 1.24	
			**Education**	**Fruit and vegetable consumption**	13	Low - lower than tertiary	**3.65**	(SD) 1.12	0.002
				mean servings per week	157	High - tertiary	**4.86**	(SD) 1.33	
						no adjustment described			
**Zeba et al. (2014)** - Funded by the Canadian International Development Agency									
cross-sectional 300 Burkina Faso	high	Burkinabe born and resident in Ouagadougou for >6 months. Excludes pregnant or lactating women and physically and mentally disabled individuals. Aged 25-60 years	**Asset score**	**Unhealthy diet**		Low (tertile score)	**19.1%**	11.1-27.3	0.002
				high in fat, sugar, low in fibre, plant protein and complex carbohydrates		Medium (tertile score)	**37.1%**	27.1-47.1	0.4
						High (tertile score)	**43.8%**	33.5-54.1	0.003
			**Education**	**Unhealthy diet**		Low (no formal)	**24.7%**	15.8-33.6	<0.001
				high in fat, sugar, low in fibre, plant protein and complex carbohydrates		Medium (elementary)	**15.7%**	8.2-23.2	0.3
						High (High school and above)	**59.6%**	49.4-69.8	<0.001
						no adjustment described			
**Houehanou et al. (2015)** - No external funding									

cross-sectional 5,830	moderate	Population-based nationally representative 2008 STEPS survey. Aged 25-64 years							
			**Income**	**Alcohol use**	3,549	Lowest (<FCFA 66,000*)	**17.8%**		0.003
Benin				consume alcohol ≥4 days/week	1,034	Low (FCFA 66,000-132,000)	**16.5%**		
					565	Middle (FCFA 132,000-198,000)	**15.9%**		
					378	High (FCFA 198,000-264,000)	**12.4%**		
				**€100 = FCFA 65,657 at time of survey*	304	Highest (>FCFA 264,000)	**11.2%**		
			**Income**	**Fruit and vegetable consumption**	3,549	Lowest (<FCFA 66,000*)	**20.2%**		<0.0001
				≤5 servings fruit and vegetables/day	1,034	Low (FCFA 66,000-132,000)	**16.7%**		
					565	Middle (FCFA 132,000-198,000)	**16.9%**		
					378	High (FCFA 198,000-264,000)	**10.3%**		
				**€100 = FCFA 65,657 at time of survey*	304	Highest (>FCFA 264,000)	**16.1%**		
						no adjustment described			
**<u>Al Ali et al. (2011)</u>** - Funded by the National Institute on Drug Abuse; EU grant Mediterranean studies of Cardiovascular disease and Hyperglycaemia									
cross-sectional	moderate	Representative sample of	**Education**	**Unhealthy diet**	351	Low (<6 years)	**49.1%**	40.5-57.7	<0.05
1,168		Aleppo residents from a		fruit and veg consumed <3 days/week	509	Medium (6-11 years)	**34.4%**	30.1-38.9	
Syria		2006 survey.			308	High (>12 years)	**16.4%**	12.6-21.1	
		Aged >25 years							
			**SES (education,**	**Unhealthy diet**	415	Low (tertile score)	**49.9%**	43-56.8	<0.05
			**employment status,**	see above	369	Medium (tertile score)	**30.5%**	25.7-35.8	
			**household income)**		384	High (tertile score)	**17.4%**	14-21.5	
			**Employment**	**Unhealthy diet**	624	Employed	**27.9%**	22.5-34	>0.05
				fruit and veg consumed <3 days/week	544	Unemployed	**38.3%**	30.9-46.4	
						no adjustment described			
**<u>Badruddin (1994)</u>** - Source of funding not reported									
cross-sectional	low	Children from lower-middle	**Class (undefined)**	**Cholesterol intake**		Age 5-9, lower-middle class	**296.9mg**	(SD) 134.6	<0.05

	233	income families with average monthly income US$115. Selected from two elementary schools. Aged 5-19 years						
				mean intake mg/day	Age 5-9, upper class	**353.9mg**	(SD) 159.7	
Pakistan								
				**Cholesterol intake**	Age 10-14, lower-middle class	**363.8mg**	(SD) 152.2	<0.05
				mean intake mg/day	Age 10-14, upper class	**469.6mg**	(SD) 173.4	
				**Cholesterol intake**	Age 15-19, lower-middle class	**318.8mg**	(SD) 134.3	<0.05
				mean intake mg/day	Age 15-19, upper class	**541.7mg**	(SD) 178.2	
			**Class (undefined)**	**Cholesterol intake**	Age 5-9, lower-middle class	**343.9mg**	(SD) 175.6	<0.05
				mean intake mg/day	Age 5-9, upper class	**486.9mg**	(SD) 157.1	
				**Cholesterol intake**	Age 10-14, lower-middle class	**275.9mg**	(SD) 129.8	<0.05
				mean intake mg/day	Age 10-14, upper class	**463.7mg**	(SD) 186.1	
				**Cholesterol intake**	Age 15-19, lower-middle class	**240.9mg**	(SD) 108.8	<0.05
				mean intake mg/day	Age 15-19, upper class	**410.7mg**	(SD) 191.5	
					no adjustment described			

Design, n	Quality	Sample	Exposure	Outcome	n	Exposure subgroup	Value	95% CI	p
Non peer-reviewed									
**WHO STEPS Côte d'Ivoire (2007)** - Source of funding not reported									
cross-sectional 4,530 Côte d'Ivoire	moderate	WHO STEPS survey. Aged 15-64 years	**Education**	**Low physical activity** <600 MET mins/week	3,442	Illiterate	**86.4%**	81.2-91.6	
						Primary	**87.4%**	81.7-93.2	
						Secondary	**89.3%**	84.8-93.8	
						Higher education	**90.1%**	85.3-94.8	
						no adjustment described			
**WHO STEPS Eritrea (2004)** - Funded by WHO, Italian Government, Eritrean Government									
cross-sectional 2,033 Eritrea	moderate	Nationally representative sample, individuals selected from six zones. Aged 15-64 years	**Education**	**Physical inactivity**	808	Lowest (no formal education)	**10.3%**		0.066
					709	Low (Grades 1-6)	**8.9%**		
					283	Middle (Grade 7-8)	**10.2%**		
					436	High (Grades 9-12)	**11.5%**		
					102	Highest (>12 years)	**11.8%**		
					2,338 total		**10.1%**		
						no adjustment described			
**WHO STEPS India (2007)** - Funded by World Bank, Indian Government									
cross-sectional 38,064 India	moderate	WHO STEPS survey in seven states. Aged 15-64	**Education Andhra Pradesh**	**Low physical activity** <600 MET mins/week	6,218	Illiterate	**62.2%**		
						Primary	**61.3%**		
						Middle	**64.2%**		
						Secondary	**73.8%**		
						Higher Secondary	**81.5%**		
						College and above	**83.8%**		
			**Education Madhya Pradesh**	**Low physical activity** <600 MET mins/week	5,853	Illiterate	**29.9%**		
						Primary	**34.8%**		
						Middle	**44.4%**		
						Secondary	**54.7%**		
						Higher Secondary	**63.2%**		
						College and above	**79.3%**		
			**Education**	**Low physical activity**	6,091	Illiterate	**75.2%**		

**Maharashtra**	<600 MET mins/week		Primary	**75.3%**
			Middle	**76.6%**
			Secondary	**82.7%**
			Higher Secondary	**85.8%**
			College and above	**93.8%**
**Education**	**Low physical activity**	4,495	Illiterate	**57.9%**
**Mizoram**	<600 MET mins/week		Primary	**52.0%**
			Middle	**62.7%**
			Secondary	**77.7%**
			Higher Secondary	**87.3%**
			College and above	**91.4%**
**Education**	**Low physical activity**	4,859	Illiterate	**63.8%**
**Kerala**	<600 MET mins/week		Primary	**59.6%**
			Middle	**63.7%**
			Secondary	**74.5%**
			Higher Secondary	**84.9%**
			College and above	**89.8%**
**Education**	**Low physical activity**	5,105	Illiterate	**55.9%**
**Tamil Nadu**	<600 MET mins/week		Primary	**57.6%**
			Middle	**62.4%**
			Secondary	**70.1%**
			Higher Secondary	**77.9%**
			College and above	**84.5%**
**Education**	**Low physical activity**	5,443	Illiterate	**57.0%**
**Uttarakhand**	<600 MET mins/week		Primary	**57.3%**
			Middle	**63.5%**
			Secondary	**67.1%**
			Higher Secondary	**78.4%**
			College and above	**89.2%**

no adjustment described

Peer reviewed

Kinra et al. (2010) - Funded by the Wellcome Trust

cross-sectional 1,983 India	high	Nationally representative sample of rural inhabitants from 1600 villages in 18 states. Aged 20-69 years	**SES (men) assets, housing**	**Low physical activity**	147	Low (asset score)	**65.2%**	57.5-72.9	0.11
				<1.69 MET	358	Middle (asset score)	**72.4%**	67.8-77	
					870	High (asset score)	**72.9%**	70-75.9	
			**SES (women) assets, housing**	**Low physical activity**	106	Low (asset score)	**66.0%**	54.5-77.4	0.084
				<1.69 MET	143	Middle (asset score)	**73.5%**	66.0-81.0	
					359	High (asset score)	**76.5%**	72-81	
						adjusted for age			

Gupta et al. (2003) - Source of funding not reported

cross-sectional 573 India	high	Serial cross-sectional surveys from the general population of Jaipur. Data taken from the most recent round. Age not reported	**Education (men)**	**Physical Inactivity**	103	Low (no formal education)	**89.3%**		0.016
				Leisure time physical activit	182	Middle-low (1-10 years)	**64.3%**		
					202	Middle-high (11-15 years)	**50.5%**		
					63	High (>16 years)	**42.9%**		
			**Education (women)**	**Physical Inactivity**	213	Low (no formal education)	**89.2%**		0.038
				Leisure time physical activit	163	Middle-low (1-10 years)	**58.9%**		
					161	Middle-high (11-15 years)	**39.8%**		
					36	High (>16 years)	**33.3%**		
						adjusted for age			

Singh et al. (2000) - Sandoz (Novartis) Foundation of Gerontologic Research (AUS); World Health Federation

cross-sectional 3,257 India	low	Representative female sample from 5 cities. Aged 25-64 years	**SES (education, occupation, income, assets, housing)**	**Sedentary**	985	High	**92.2%**		<0.01
				Indian classification of	790	Medium-high	**71.4%**		
				activities (occupational, hou	774	Medium	**42.3%**		
					602	Medium-low	**14.9%**		
					206	Low	**8.7%**		

Singh et al. (1997) - Source of funding not reported

cross-sectional 1,767 India	high	Residents of two villages in rural north India. Aged 25-64 years	**SES (men) education, occupation, income, assets, housing**	**Sedentary lifestyle***	147	High	**44.20%**	<0.05
					147	Middle-high	**34.60%**	
					287	Middle-low	**3.50%**	
					313	Low	**not reported**	
			**education, occupation, income, assets, housing**	**Sedentary lifestyle***	115	High	**13%**	<0.01
					112	Middle-high	**58%**	
					313	Middle-low	**20.40%**	
					335	Low	**4.40%**	
				*walk <14.5km/week, climb <20flights stairs or no moderate activity 5 days/week				

no adjustment described

Zaman et al. (2012) - Funded by the Byrraju Foundation and the Initiative for Cardiovascular Health Research in Developing Countries

cross-sectional 4,535 India	moderate	Representative sample from 20 villages in rural Andhra Pradesh. Aged >30 years	**Education (men)**	**Physical inactivity**	1,311	High (primary or higher)	**33.2%**	<0.001
				reports 'almost none' durin[illegible]	895	Low (no formal education)	**23.2%**	
			**Education (women)**	**Physical inactivity**	1,074	High (primary or higher)	**61.6%**	<0.001
				reports 'almost none' durin[illegible]	1,255	Low (no formal education)	**49.3%**	
			**Occupation (men)**	**Physical inactivity**	434	Skilled*	**46.3%**	<0.001
				reports 'almost none' durin[illegible]	1,501	Unskilled	**12.3%**	
			**Occupation (women)**	**Physical inactivity**	88	Skilled*	**51.4%**	<0.001
				reports 'almost none' durin[illegible]	872	Unskilled	**12.2%**	
				**skilled manual labour, owner of business/farmer office worker/non-professional or professional)*				

			**Income (men)**	**Physical inactivity**	912	High (>2000 INR/month)	**34.1%**		<0.001
				reports 'almost none' durin	717	Middle (1200-1999 INR/month)	**21.4%**		
					577	Low (0-1199 INR/month)	**30.5%**		
			**Income (women)**	**Physical inactivity**	945	High (>2000 INR/month)	**57.3%**		<0.001
				reports 'almost none' durin	550	Middle (1200-1999 INR/month)	**50.4%**		
					834	Low (0-1199 INR/month)	**54.3%**		
						no adjustment described			

Gupta et al. (2012) - Funded by the South Asian Society of Atherosclerosis and Thrombosis

cross-sectional 6,198 India	high	General population in middle-class areas of 11 cities, excluded house-bound, pregnant and those likely to die within 6 months. Aged 18-75 years	**Education**	**Low physical activity**	1,248	Low (0-10 years)	**37.2%**		
				no regular work-related or l	2,956	Middle (11-15 years)	**45.5%**		
					1,366	High (>15 years)	**35.5%**		
			**Occupational Class (British Social Register, housewife = husband)**	**Low physical activity**	1,287	Low (4-5)	**47.4%**		
				see above	1,677	Middle (3 manual/non-manual)	**43.7%**		
					3,018	High (1-2)	**39.1%**		
			**SES (self-assessed)**	**Low physical activity**	374	Low (score 1-3)	**42.3%**		
				no regular work-related or l	3,622	Middle (score 4-6)	**40.1%**		
					1,114	High (score 7-10)	**36.9%**		
						adjusted for age and sex			

Reddy et al. (2007) - Funded by the Indian Ministry of Health; WHO

cross-sectional 19,969 India	high	Industrial workers and their relatives from ten urban sites across India. Aged 20-69 years.	**Education (men)**	**Physical activity**	1,611	High (postgraduate)	**1**	OR	<0.001
				leisure time physical activity	2,607	Middle-high (secondary-tertiary)	**0.9**	0.8-1.1	
					5,820	Middle-low (primary-secondary)	**0.7**	0.6-0.8	
					1,859	Low (none-primary)	**0.2**	0.18-0.25	
			**Education (women)**	**Physical activity**	1,611	High (postgraduate)	**1**	OR	<0.001
				leisure time physical activity	2,607	Middle-high (secondary-tertiary)	**0.9**	0.7-1.1	

					5,820	Middle-low (primary-secondary)	**0.6**	0.5-0.8	
					1,859	Low (none-primary)	**0.3**	0.2-0.4	

adjusted for age, occupation

<u>**Kar et al. (2010)**</u> - Source of funding not reported

cross-sectional<br>400<br>India	moderate	Residents at eight sites in Northern India. Aged >30 years	**Literacy**	**Sedentary**	226	Literate	**1.3**	0.7-2.5	0.5
					177	Illiterate	**1**	OR	

adjusted for age, sex, literacy, place of residence

<u>**Anjana (2014)**</u> - Indian Council of Medical Research

cross-sectional<br>14,227<br>India	moderate	Data from Indian council of medical research-India diabetes (ICMR-INDAB). Aged >20 years old	**Income**	**Inactive**<br><600 MET minutes/week	Low (below median)	**42.0%**	<0.001
					High (above median)	**58.0%**	
			**Income**	**Highly active**<br>>1200 MET minutes/week	Low (below median)	**62.7%**	<0.001
					High (above median)	**37.3%**	
			**Education**	**Inactive**<br><600 MET minutes/week	Low (Illiterate)	**28.6%**	<0.001
					Middle-low (primary/secondary)	**61.2%**	
					Middle-high (Undergraduate)	**8.8%**	
					High (Postgraduate)	**1.3%**	
			**Education**	**Highly active**<br>>1200 MET minutes/week	Low (Illiterate)	**39.1%**	<0.001
					Middle-low (primary/secondary)	**57.1%**	
					Middle-high (Undergraduate)	**3.5%**	
					High (Postgraduate)	**0.3%**	
			**SES (education, occupation, income)**	**Inactive**<br><600 MET minutes/week	Low (Score <11/29)	**11.2%**	<0.001
					Middle (Score 11-25/29)	**26.5%**	
					High (Score 25-29/29)	**62.3%**	

			**SES (education, occupation, income)**	**Highly active**		Low (Score <11/29)	**21.9%**	<0.001
				>1200 MET minutes/week		Middle (Score 11-25/29)	**38.8%**	
						High (Score 25-29/29)	**39.3%**	
						no adjustment described		
**Anjana et al. (2015)** - Funded by Lilly Diabetes; International Diabetes Federation; Global Health Institute at Emory University								
cross-sectional<br>1,281<br>India	moderate	Obese and overweight individuals with high blood sugar but not diagnosed with diabetes. Pregnant women and seriously unwell excluded. Participants southern India from baseline survey from D-CLIP (diabetes community lifestyle improvement program). Age range not reported	**Income**	**Active**		High (INR >25000/month)	**41.0%**	<0.05
				>150 mins/week exercise		Low (INR<25000/month)	**59.0%**	
			**Income**	**Inactive**		High (INR >25000/month)	**29.2%**	
				<150 mins/week exercise		Low (INR<25000/month)	**70.1%**	
				variables; age, gender, education, income				
			**Education**	**Active**		High (Graduate)	**68.8%**	<0.05
				>150 mins/week exercise		Lower (Non-graduate)	**31.2%**	
			**Education**	**Inactive**		High (Graduate)	**60.0%**	
				<150 mins/week exercise		Lower (Non-graduate)	**40.0%**	
						adjusted for age, gender, education, income		
**Safraj et al. (2012)** - No external funding								
cross-sectional<br>74,147<br>India	moderate	PROFILE cohort - rural Keralan adults.<br>Aged >20 years	**SES (assets, services)**	**Sedentary**	9,557	Highest (SEP$)	**17.1%**	
				Leisure and occupation phys	23,468	Upper Middle	**15.2%**	
					32,577	Lowe Middle	**8.7%**	
					8,545	Lowest	**4.7%**	
			**SES (assets, services)**	**Moderate physical activity**	9,557	Highest (SEP4)	**10.3%**	
				Leisure and occupation phys	23,468	Upper Middle	**7.8%**	
					32,577	Lowe Middle	**4.2%**	
					8,545	Lowest	**2.7%**	
			**SES (assets, services)**	**Heavy physical activity**	9,557	Highest (SEP4)	**5.7%**	
				Leisure and occupation phys	23,468	Upper Middle	**3.3%**	
					32,577	Lowe Middle	**3.7%**	

					8,545	Lowest	**1.0%**	
						no adjustment described		

Dewi et al. (2010) - Funded by Provincial Health Office of Yogyakarta Special Regency, Indonesia

cross-sectional	moderate	Representative sample of	**Income (male) 15-35 years**	**Inactivity**		Low (<mean income	**38%**	
3,285		residents of Yogyakarta.		<105 mins active time/week		High (> mean income)	**37%**	
Indonesia		Aged 15-75 years	**35-54 years**			Low (<mean income	**23%**	
						High (> mean income)	**36%**	
			**55-75 years**			Low (<mean income	**29%**	
						High (> mean income)	**33%**	
			**Income (female) 15-35 years**	**Inactivity**		Low (<mean income	**33%**	
				<105 mins active time/week		High (> mean income)	**43%**	
			**35-54 years**			Low (<mean income	**27%**	
						High (> mean income)	**42%**	
			**55-75 years**			Low (<mean income	**38%**	
						High (> mean income)	**31%**	
						no adjustment described		

Mumu et al. (2014) - Source of funding not reported

cross-sectional<br>374	moderate	Type 2 diabetics who had been diagnosed for >1 year from 9 health centres around Dhaka. Aged >20 years	**Education**	**Sedentary**	112	Low (up to primary)	**26.8%**	0.654
Bangladesh				<30 mins planned exercise/	174	Medium (up to higher secondary)	**22.4%**	
					88	High (graduate and above)	**26.1%**	
						no adjustment described		

Dhungana et al. (2014) - Source of funding not reported

cross-sectional<br>406<br>Nepal	high	Rural community in the Sindhuli district.<br>Aged 20-50 years	**Caste**	**Insufficient physical activity**		High (Brahman/Chhetri)	**47.5%**	0.06
				<150 minutes moderate physical activity/		Middle (Adhibasi/Janajati)	**51.6%**	
						Low (Dalits)	**28.6%**	
			**Education**	**Insufficient physical activity**		Low (No formal education)	**45.7%**	0.0286
				<150 minutes moderate physical activity/		Middle-low (lower than primary)	**37.9%**	

						Middle-high (primary)	**50.0%**		
						High (Secondary and higher)	**61.4%**		
			**SES (education, occupation, income)**	**Insufficient physical activity**		Lowest	**54.1%**		0.8
				<150 minutes moderate physical activity/\		Middle-low	**42.6%**		
						Middle-high	**50.0%**		
						Highest	**62.5%**		

no adjustment described

Vaidya and Krettek (2014) - Funded by the Wilhelm & Martina Lundgren's Foundation; University of Gothenburg

cross-sectional	moderate	Adults from six peri-urban	**Education (men)**	**Adequate physical activity** mins vigorous exercise/week		High (highschool or above)	**68.50%**	55.9-81	
640		sites near Kathmandu.				Medium-high (secondary)	**86.40%**	86.4-94.3	
Nepal		Aged 25-59 years				Medium-low (primary)	**87.80%**	80.3-95.3	
						Low (informal)	**81.50%**	66.5-96.4	
			**Education (women)**	**Adequate physical activity** mins vigorous exercise/week		High (highschool or more)	**67.10%**	56-78.2	
						Medium-high (secondary)	**77.40%**	70-84.8	
						Medium-low (primary)	**80.80%**	74.9-86.7	
						Low (informal)	**80.20%**	74.4-86	
			**Education**	**Median MET mins/week** minutes and unadjusted odds	94	High (highschool or more)	**218m**	**2.99**	1.65-5.46
					165	Medium-high (secondary)	**482m**	**1.42**	0.86-2.32
					208	Medium-low (primary)	**613m**	**0.86**	0.55-1.33
					173	Low (informal)	**600m**	**1**	OR

adjusted for age, sex, occupation, education, ethnicity

Katulanda et al. (2013) - Funded by the National Science Foundation (SL); Diabetes Endocrinology and Metabolism (UK); National Institute for Health Research (UK)

cross-sectional	moderate	2005/6 population-based	**Education**	**Physical activity**	267	Low (no formal education)	**5279m**	(SD) 5376	
4,485		sample excluding		Mean MET minutes/week	809	Medium-low (primary)	**5284m**	(SD) 5004	<0.01
Sri Lanka		institutionalised individuals			3,379	Medium-high (secondary)	**4608m**	(SD) 4127	<0.01
		and those from the war-			129	High (tertiary)	**2248m**	(SD) 2297	<0.01
		torn northern zone.				no adjustment described			
		Aged >18 years							
			**Education (men)**	**Physical activity**		Low (no formal education)	**5940m**	(SD) 6094	

	Mean MET minutes/week		Low (no formal education)	**6844m**	(SD) 6246	<0.01
			Medium-low (primary)	**5309m**	(SD) 5252	<0.01
			Medium-high (secondary)	**2284m**	(SD) 2655	<0.01
			no adjustment described			
**Education (women)**	**Physical activity**		Low (no formal education)	**5103m**	(SD) 5170	<0.01
	Mean MET minutes/week		Medium-low (primary)	**4335m**	(SD) 3770	<0.01
			Medium-high (secondary)	**4128m**	(SD) 3038	<0.01
			High (tertiary)	**2197m**	(SD) 1678	<0.01
			no adjustment described			
**Education**	**Physical activity**	267	Low (no formal education)	**1**	OR	
	active vs inactive*	809	Medium-low (primary)	**0.9**	0.7-1.3	>0.05
		3,379	Medium-high (secondary)	**1.1**	0.8-1.5	>0.05
		129	High (tertiary)	**3.6**	2.2-6.0	<0.001
			adjusted but variables not described			
	Positive value= inactive correlate					

**using IPAQ definition: less than 3 days vigorous activity for 20 mins; less than 5 days of 30 mins moderate activity and/or walking 30 mins per day; less than 600 MET mins/week from any activity*

**Education (men)**	**Physical activity**		Low (no formal education)	**1**	OR	
	active vs inactive*		Medium-low (primary)	**0.8**	0.4-1.5	>0.05
			Medium-high (secondary)	**1.1**	0.6-2.1	>0.05
			High (tertiary)	**2.8**	1.2-6.6	<0.001
	Positive value= inactive correlate		adjusted but variables not described			

**using IPAQ definition: less than 3 days vigorous activity for 20 mins; less than 5 days of 30 mins moderate activity and/or walking 30 mins per day; less than 600 MET mins/week from any activity*

**Education (women)**	**Physical activity**		Low (no formal education)	**1**	OR	
	active vs inactive*		Medium-low (primary)	**1.1**	0.8-1.5	>0.05
			Medium-high (secondary)	**1.1**	0.8-1.6	>0.05
			High (tertiary)	**4.4**	2.2-8.9	<0.001
	Positive value= inactive correlate		adjusted but variables not described			

**using IPAQ definition: less than 3 days vigorous activity for 20 mins; less than 5 days of 30 mins moderate activity and/or walking 30 mins per day; less than 600 MET mins/week from any activity*

<u>**Sossa et al. (2013)**</u> - Funded by the Canadian International Development Agency

cohort	moderate	Healthy adults sampled	**Education**	**Sedentary**		High school	**126.6m** (+/-) 13.1	
208		from a large city, small		minutes/day		Primary schooling	**97.2m** (+/-) 84.4	
Benin		town and rural area.				No Schooling	**126.6m** (+/-) 103.1	
		Aged 25-60 years						
			**Education**	**Vigorous or moderate activity**		High school	**140.7m** (+/-) 146.8	
				minutes/day		Primary schooling	**185.5m** (+/-) 151.4	
						No Schooling	**199.4m** (+/-) 166.7	
			**Wealth (assets)**	**Sedentary**		High	**127.4m** (+/-)102.8	
				minutes/day		Medium	**107.9m** (+/-) 93.9	
						Low	**71.8m** (+/-) 67.3	
			**Wealth (assets)**	**Vigorous or moderate activity**		High	**110.7m** (+/-)121.6	
				minutes/day		Medium	**180.8m** (+/-)160.4	
						Low	**216.9m** (+/-)160.7	

no adjustment described

<u>**Rahlenbeck and Gebre-Yohannes (1998)**</u> - Funded by the Ethiopian Science and Technology Commission resources

cross-sectional	low	Medical students at Gondar						
66		college in north-west Ethiopia.						
		Age range not reported (mean						
		20.8 years, SD=1.6)						
			**Income (parental)**	**Sedentary lifestyle**	17	High (>US$150/month)	**18%**	0.421
Ethiopia				<2h/week moderate or <1h,	49	Low (<US$150/month)	**10%**	

no adjustment described

<u>**Bunker et al. (1996)**</u> - Funded by the National Institute of Health

cross-sectional	moderate	Civil servants selected from	**SES (seniority) men**	**Physical activity**	401	Senior staff	**20.2m**	<0.001
713		3 different sites at three		minutes/day walking	635	Junior staff	**29.3m**	
Nigeria		time periods: 1988, 1990 and						
		1992. This data from the	**SES (seniority) men**	**Physical activity**	401	Senior staff	**86 METS**	<0.001
		1992 Benin wave.		METS/week	635	Junior staff	**111.5 METS**	
		Aged 25-54 years						
			**SES (seniority) women**	**Physical activity**	157	Senior staff	**16.2m**	<0.001

				mins/day walking	234	Junior staff	**25.6m**		
			**SES (seniority) women**	**Physical activity**	157	Senior staff	**[illegible]9.6 METS**		>0.05
				METS/week	234	Junior staff	**[illegible]8.5 METS**		

no adjustment described

<u>Oyewole et al. (2014)</u> - Source of funding not reported

cross-sectional 122	moderate	Diabetics recruited from a local clinic, excluded acutely unwell, the aged, amputations, visual impairment. Aged >20 years	**Education**	**Inactive**		Low (illiterate or primary)	**39.6%**		0.16
Nigeria				does not meet WHO recommendation*		Middle (secondary)	**30.0%**		
						High (diploma or certificate)	**19.4%**		

**150 min of moderate intensity activity per week, 75 min of vigorous intensity <150 mins moderate activity/week, or an equivalent combination of moderate and vigorous intensity activity*

no adjustment described

<u>Zeba et al. (2014)</u> - Funded by the Canadian International Development Agency

cross-sectional 330 Burkina Faso	high	Burkinabe born and resident in Ouagadougou for >6 months. Excludes pregnant or lactating women and physically and mentally disabled individuals.	**Asset score**	**Physical activity**		Low (tertile score)	**5.3h**	(SD) 2.7	<0.001
				mean hours of physical activity equivalent		Medium (tertile score)	**4.3h**	(SD) 2.2	
						High (tertile score)	**3.4h**	(SD) 1.6	
		Aged 25-60 years	**Asset score**	**Sedentary time**		Low (tertile score)	**10.7h**	(SD) 3.3	<0.001
				mean hours of sedentary time equivalent		Medium (tertile score)	**11.4h**	(SD) 3.1	
						High (tertile score)	**12.4h**	(SD) 2.8	
			**Education**	**Physical activity**		Low (no formal)	**4.8h**	(SD) 2.4	0.002
				mean hours of physical activity equivalent		Medium (elementary)	**4.6h**	(SD) 2.5	
						High (High school and above)	**3.8h**	(SD) 2.1	
			**Education**	**Sedentary time**		Low (no formal)	**11.1h**	(SD) 3.2	0.251
				mean hours of sedentary time equivalent		Medium (elementary)	**11.6h**	(SD) 3.1	
						High (High school and above)	**11.7h**	(SD) 3.0	

no adjustment described

Oanh et al. (2008) - Funded by Atlantic Philanthropies

cross-sectional	high	Representative STEPS	**Education**	**Insufficient physical activity**	less than primary	**1**	OR, p for trend <0.001	
1,776		survey from Ho Chi Minh		<600 MET mins/week	primary completed	**0.88**	0.56-1.37	
Vietna,		city.			secondary completed	**0.93**	0.55-1.56	
		Aged 25-64 years			high school completed	**1.09**	0.68-1.75	
					some college	**1.32**	0.63-2.76	
			**Income**	**Insufficient physical activity**	VND <1,000,000/month	**1**	OR, p for trend <0.001	
				<600 MET mins/week	VND 1,000,000-3,000,000	**1.42**	1.02-2	<0.05
					VND 3,000,000-5,000,000	**1.51**	0.94-2.43	
					VND >5,000,000	**1.77**	1.05-2.79	<0.05
			**Wealth (assets)**	**Insufficient physical activity**	Lowest	**1**	OR, p for trend <0.001	
				<600 MET mins/week	Second	**1.29**	0.9-1.84	
					Middle	**1.67**	1.26-2.21	<0.05
					Fourth	**1.87**	1.15-3.04	<0.05
					Highest	**1.86**	1.29-2.66	<0.05

adjusted for age group, sex, ethnicity, education, occupation, household SES, tobacco use, alcohol consumption

Hosey et al. (2014) - Funded by Uniformed Services University of the Health Sciences

cross-sectional	moderate	WHO STEPwise survey using							
1,638		randomly selected	Education (men)	Sedentary	99	High (Post-secondary >13years)	**0.5**	0.28-0.88	0.025
FS Micronesia		households in Pohnpei.		<30 mins/day of moderate activity on 5 or more days per week	183	Medium (Secondary, 9-12 years)	**1.01**	0.63-1.64	
		Aged 25-64			315	Low (Primary <9 years)	**1**	OR	
			Education (women)	Sedentary	99	High (Post-secondary >13years)	**0.65**	0.22-1.94	0.258
					183	Medium (Secondary, 9-12 years)	**0.68**	0.42-1.11	
					315	Low (Primary <9 years)	**1**	OR	

no adjustment described

Abd-Elhady et al. (2007) - Source of funding not reported

cross-sectional 283	low	Diabetic patients attending Alf-Maskan outpatient clinic in East Cairo.							
			Education	**No exercise**	78	high (undefined)	**74.0%**		
Egypt		Age not reported		undefined	160	medium	**70.6%**		
					45	low (illiterate)	**73.3%**		
			**Education**	**Regular exercise**	78	High (undefined)	**11.5%**		
				undefined	160	Medium (undefined)	**8.1%**		
					45	Low (Illiterate)	**6.7%**		
						no adjustment described			

Al Ali et al. (2011) - Funded by the National Institute on Drug Abuse; EU grant Mediterranean studies of Cardiovascular disease and Hyperglycaemia

cross-sectional 1,168	moderate	Representative sample of Aleppo residents from a							
Syria		2006 survey.	**Education**	**Physical inactivity**	351	Low (<6 years)	**89.2%**	84.7-92.5	<0.05
		Aged >25 years		<1/week sport or ≥15mins b	509	Medium (6-11 years)	**85.0%**	79.7-89.1	
					308	High (>12 years)	**71.0%**	61.8-78.8	
			**SES (education, assets,**	**Physical inactivity**	415	Low (tertile score)	**90.2%**	85.7-93.4	<0.05
			**employment status,**	see above	369	Medium (tertile score)	**83.6%**	77-88.6	
			**household income)**		384	High (tertile score)	**71.9%**	66.3-79.2	
			**Occupation**	**Physical inactivity**	624	Employed	**75.5%**	68.3-81.4	<0.05
				<1/week sport or ≥15mins b	544	Unemployed	**88.3%**	83.6-91.8	
						no adjustment described			

Design, n	Quality	Sample	Exposure	Outcome	n	Exposure subgroup	Value	95% CI	p

Non-peer reviewed

WHO STEPS India (2007) - Funded by World Bank, Indian Government

Design, n	Quality	Sample	Exposure	Outcome	n	Exposure subgroup	Value	95% CI	p
cross-sectional 38,064 India	moderate	WHO STEPS survey in seven states. Aged 15-64	**Education Andhra Pradesh**	**current daily smoker**	6,218	Illiterate	**23.10%**		
						Primary	**23.00%**		
						Middle	**16.30%**		
						Secondary	**12.50%**		
						Higher Secondary	**6.90%**		
						College and above	**8.10%**		
			**Education Madhya Pradesh**	**current daily smoker**	5,853	Illiterate	**20.60%**		
						Primary	**37.00%**		
						Middle	**24.80%**		
						Secondary	**18.70%**		
						Higher Secondary	**15.00%**		
						College and above	**11.60%**		
			**Education Maharashtra**	**current daily smoker**	6,091	Illiterate	**12.90%**		
						Primary	**14.40%**		
						Middle	**8.90%**		
						Secondary	**7.60%**		
						Higher Secondary	**7.70%**		
						College and above	**7.30%**		
			**Education Mizoram**	**current daily smoker**	4,495	Illiterate	**50.90%**		
						Primary	**55.40%**		
						Middle	**45.80%**		
						Secondary	**37.60%**		
						Higher Secondary	**39.20%**		
						College and above	**39.60%**		
			**Education Kerela**	**current daily smoker**	4,859	Illiterate	**17.70%**		
						Primary	**30.10%**		
						Middle	**22.20%**		
						Secondary	**13.10%**		
						Higher Secondary	**4.80%**		
						College and above	**6.50%**		
			**Education**	**current daily smoker**	5,105	Illiterate	**14.90%**		

			**Tamil Nadu**			Primary	**19.70%**	
						Middle	**17.30%**	
						Secondary	**13.40%**	
						Higher Secondary	**7.20%**	
						College and above	**5.90%**	
			**Education Uttarakhand**	**current daily smoker**	5,443	Illiterate	**25.60%**	
						Primary	**30.40%**	
						Middle	**21.20%**	
						Secondary	**18.60%**	
						Higher Secondary	**11.00%**	
						College and above	**11.20%**	
						No adjustment described		

WHO STEPS Côte d'Ivoire (2007) - Source of funding not reported

cross-sectional 4,530	moderate	WHO STEPS survey. Aged 15-64 years	**Education**	**Current smoker**	4,491	Illiterate	**13.40%**	8.2-18.7
				currently smoke tobacco products including cigarettes, cigars & pipes		Primary	**11.50%**	8.7-14.2
Côte d'Ivoire						Secondary	**16.50%**	12.8-20.2
						Higher education	**11.20%**	8.5-13.8
			**Education**	**Daily smoker** % of current smokers	661	Illiterate	**34.20%**	24.2-44.3
						Primary	**24.40%**	18.2-30.6
						Secondary	**32.40%**	22.1-42.7
						Higher education	**24.90%**	13.1-36.7
						No adjustment described		

WHO STEPS Zambia (2008) - Funded by Zambian Ministry of Health; WHO

cross-sectional 1,928	moderate	WHO STEPS survey in Lusaka district, 67% female. Aged >25 years	**Education**	**"Smoker"**		No formal education	**1**	OR
Zambia						Primary completed	**1.12**	0.69-2.09
						Secondary completed	**0.92**	0.58-1.47
						College or higher completed	**0.62**	0.36-1.06
						No adjustment described		

Peer-reviewed

Jindal et al. (2006) - Funded by Indian Council of Medical Research

cross-sectional	low	Residents from urban and	**SES (undefined)**	**Cigarettes**		High	**1**	OR

73,605		rural areas of Bangalore,		current or former cigarette smoker	Medium	**1.281**	1.123-1.463	
India		Chandigarh, Delhi, Kanpur. Aged >15 years			Low	**1.151**	0.986-1.344	
			**SES (undefined)**	**Bidis** current or former use	High	**1**	OR	
					Medium	**3.872**	3.234-4.529	
					Low	**11.138**	9.365-13.247	
			**SES (undefined)**	**Tobacco smoking** any - cigarettes, bidis, hookah	High	**1**	OR	
					Medium	**2.101**	1.889-2.336	
variables; undefined					Low	**4.161**	3.717-4.658	
					Adjusted			

<u>Bonu et al. (2005)</u> - <u>Source of funding not reported</u>

cross-sectional 22,68[illegible]	high	Hospitalised participants of the 1995/6 Indian National Sample Survey. Aged >10 years	**Current regular tobacco user**	**Poverty**	Non-user	**1**	OR	
India				borrowing/financial distress during hospitalisation	Tobacco user	**1.35**	1.11-1.63	<0.01

adjusted for head of household, age, sex, level of schooling, marital status, state. number of days of hospitalisation used to control for severity of illness.

<u>Hashibe et al. (2003)</u> - Funded by Assoc. Int Cancer Researc; Imperial Cancer Research Fund; National Cancer Institute (USA) & UCLA Jonnson Cancer Center Foundation

case-control	high	Study examining SES and	**Income**	**Tobacco chewing**	Low (INR<1500	**34.30%**		
47,773		premalignant oral lesions in			Middle-low (INR 1500-3000)	**22.90%**		
India		Kerala. Data is presented			Middle-high (INR 3001-5000)	**17.00%**		
		linking NCD risk factors with SES markers for the 47,773			High (INR>5000)	**11.20%**		<0.0001
		controls only. Aged >35 years	**Education**	**Tobacco chewing**	Low (None/illiterate)	**48.20%**		
					Middle-low (None/literate)	**45.70%**		
					Middle (Primary)	**33.70%**		
					Middle-high (Middle school)	**26.40%**		
					High (>High schools)	**12.40%**		<0.0001
			**Occupation**	**Tobacco chewinhg**	Manual	**29.00%**		

							Teacher/office	**10.90%**	
							Business	**21.10%**	
							Retired	**34.90%**	
							Other	**23.80%**	<0.0001
				**Income**	**Smoking**		Low (INR<1500	**28.80%**	
							Middle-low (INR 1500-3000)	**22.90%**	
							Middle-high (INR 3001-5000)	**20.20%**	
							High (INR>5000)	**16.60%**	<0.0001
				**Education**	**Smoking**		Low (None/illiterate)	**19.70%**	
							Middle-low (None/literate)	**23.30%**	
							Middle (Primary)	**29.40%**	
							Middle-high (Middle school)	**30.90%**	
							High (>High schools)	**23.80%**	<0.0001
				**Occupation**	**Smoking**		Manual	**22.60%**	
							Teacher/office	**28.40%**	
							Business	**54.30%**	
							Retired	**40.70%**	
							Other	**52.30%**	<0.0001
							No adjustment described		
**Deepa et al. (2011)** - Source of funding not reported									
cohort	1,122	moderate	Urban residential colonies of Chennai. Aged >20y	**Income**	**Current smokers**	526	Middle (INR 5,501-10,000/month	**3%**	
India					habitual smoker regardless of quantity	596	Low (INR 2,500-5,500/month)	**12.60%**	
							No adjustment described		
**Corsi et al. (2014)** - Funded by Byrraju Found; Initiative for CV Health Research in Dev. Countries; National Health and Med Research Council (AUS); the George Found.									
cross-sectional		high	Representative sample from	**Education (men)**	**Ever smoker**	2,205	Low (None/Illiterate)	**64%**	
4,534			20 villages in rural Andhra		current or former smoker		Medium (Primary)	**57.90%**	
India			Pradesh.				High (Secondary or higher)	**37.70%**	
			Aged >20 years				No adjustment described		
				**Education (women)**	**Ever smoker**	2,329	Low (None/Illiterate)	**12.40%**	
							Medium (Primary)	**3.20%**	
							High (Secondary or higher)	**1.10%**	
							No adjustment described		

			**Education**	**Current smoker**	4,535	Low (None/Illiterate)	**3.25**	2.54-4.16	<0.01
						Medium (Primary)	**1.87**	1.13-2.44	
						High (Secondary or higher)	**1**	OR	
						adjusted for age, sex, occupation, income			
			**Income (men)**	**Ever smoker**	2,205	Extremely Low (<US$0.50/day)	**62.20%**		
						Very Low ($0.50-1/day)	**57.50%**		
						Low (US$1-2/day)	**55.90%**		
						Higher (US$>2/day)	**50.70%**		
						No adjustment described			
			**Income (women)**	**Ever smoker**	2,329	Extremely Low (<US$0.50/day)	**19.10%**		
						Very Low ($0.50-1/day)	**7.30%**		
						Low (US$1-2/day)	**7.70%**		
						Higher (US$>2/day)	**4.30%**		
						No adjustment described			
			**Income (men)**	**Current smoker**	4,535	Extremely Low (<US$0.50/day)	**1.5**	1.06-2.16	<0.05
						Very Low ($0.50-1/day)	**1.15**	0.88-1.49	
						Low (US$1-2/day)	**1.12**	0.89-1.42	
						Higher (US$>2/day)	**1**	OR	
						adjusted for age, sex, occupation, income			
**<u>Kinra et al. (2010)</u>** - Funded by the Wellcome Trust									
cross-sectional 1,983	high	Nationally representative sample of rural inhabitants from 1600 villages in 18 states.	**SES (men) assets, housing**	**Smoker**	147	Low (asset score)	**36.80%**	29.6-44.1	<0.001
India		Aged 20-69 years		daily tobacco smoking at any time in the last 6 months	358	Middle (asset score)	**28.10%**	23.5-32.7	
					870	High (asset score)	**14.70%**	12.3-17.1	
			**SES (women) assets, housing**	**Smoker**	106	Low (asset score)	**1.20%**	0-2.9	0.2
					143	Middle (asset score)	**1.10%**	0-2.6	
					359	High (asset score)	**0.30%**	0-0.8	
						adjusted for age			
**<u>Neufeld et al. (2005)</u>** - No external funding									
cross-sectional	high	Data from the 1995/6 Indian	**Poverty***	**Smoke tobacco**	303416	High (above poverty line)	**1**	OR	

471,143		National Sample Survey. Aged >10 years		regular use, any form	167,727	Low (below poverty line)	**1**	1-1.1	
India									
			**Caste****	**Smoke tobacco**	334,512	High	**1**	OR	
					136,631	Low	**1.4**	1.3-1.5	
			**Education**	**Smoke tobacco**	273,069	High (formal education)	**1**	OR	
					188,956	Low (no formal education)	**1.7**	1.6-1.8	
			**Poverty***	**Chew tobacco**	303,416	High (above poverty line)	**1**	OR	
				regular use, any form	167,727	Low (below poverty line)	**1.5**	1.4-1.6	
			**Caste****	**Chew tobacco**	334,512	High	**1**	OR	
					136,631	Low	**1.5**	1.4-1.6	
			**Education**	**Chew tobacco**	273,069	High (formal education)	**1**	OR	
					188,956	Low (no formal education)	**1.2**	1.1-1.3	

**Planning commission of india definition - income required to ensure adequate intake of calories (INR 2,100 urban; INR 2,400 rural)*
***Scheduled Castes and tribes - identified in the Indian Constituion as especially disadvantaged or needy*

adjusted for age group, gender caste, income, residence, education

<u>Dixit et al. (2015)</u> - No external funding

cross-sectional	moderate	Door-to-door survey of	**Education (Men)**	**Smoke tobacco**	274	Low (No schooling)	**37.50%**		<0.001
				use smoke/smokeless					
1,410		villagers in rural Jaipur.		tobacco prodcut nearly	278	Medium-low (1-5years)	**33.45%**		
				every day, or					
India		Aged >18 years		every day for >1 month	237	Medium-high (6-12 years)	**32.91%**		
					165	High (>12 years)	**13.93%**		
			**Education (Men)**	**Chew tobacco**	274	Low (No schooling)	**28.46%**		<0.001
					278	Medium-low (1-5years)	**17.27%**		
					237	Medium-high (6-12 years)	**10.97%**		
					165	High (>12 years)	**10.30%**		
			**Education (Women)**	**Smoke tobacco**	176	Low (No schooling)	**10.97%**		0.06
					127	Medium-low (1-5years)	**4.72%**		
					108	Medium-high (6-12 years)	**0%**		
					45	High (>12 years)	**0%**		
			**Education (Women)**	**Chew tobacco**	176	Low (No schooling)	**7.95%**		0.51
					127	Medium-low (1-5years)	**9.44%**		
					108	Medium-high (6-12 years)	**12.96%**		

						45	High (>12 years)	**4.44%**		
							No adjustment described			
**Gupta et al. (2003)** - Source of funding not reported										
cross-sectional	573	high	Serial cross-sectional surveys from the general population of Jaipur. Data taken from the most recent round.	**Education (men)**	**Smoker**	103	Low (no formal education)	**54.40%**		
India			Age not reported		past or present use of any tobacco product	182	Middle-low (1-10 years)	**42.90%**		
						202	Middle-high (11-15 years)	**28.70%**		
						63	High (>16 years)	**23.80%**		
				**Education (women)**	**Smoker**	213	Low (no formal education)	**28.20%**		
						163	Middle-low (1-10 years)	**3.10%**		
						161	Middle-high (11-15 years)	**0.60%**		
						36	High (>16 years)	**2.80%**		
							adjusted for age			
**Menon et al. (2015) -** Source of funding not reported										
cross-sectional	84,45[illegible]	moderate	Representative sample from Kerala state. Aged >18 years	**Poverty***	**Smoker**	62,975	Above state poverty line	**6.70%**		
India						21,481	Below state poverty line	**12.30%**		
				**Poverty***	**Smoker**	62,975	Above state poverty line	**1**	OR	
						21,481	Below state poverty line	**1.94**	1.84-2.04	<0.0001
**Kerelan government definition: families which meet >4 of the following: no land or less than 5 cents, no house/dilapidated house; no sanitation latrine; no regular employed person in house; no access to safe drinking water; women-headed household or presence of widow or divorce; scheduled class/tribe; mentally retarded or (rural) disabled member in the family; no colour TV (urban) or family with an illiterate adult member*										
							No adjustment described			
**Singh et al. (2000)** - Sandoz (Novartis) Foundation of Gerontologic Research (AUS); World Health Federation										
cross-sectional	3,257	low	Representative female sample from 5 cities. Aged 25-64 years	**SES (education, occupation, income, assets, housing)**	**Use tobacco**	985	High	**8.10%**		0.09
India					uses tobacco >1/week	790	Medium-high	**5.90%**		
						774	Medium	**6.70%**		
						602	Medium-low	**7.90%**		
						206	Low	**8.70%**		
							No adjustment described			
**Singh et al. (2000)** - Sandoz (Novartis) Foundation of Gerontologic Research (AUS); World Health Federation										

cross-sectional 1,767	high	Residents of two villages in rural north India.	**SES (education, occupation,**	**Use tobacco**	985	High	**8.10%**	0.09
		Aged 25-64 years	**income, assets, housing)**	uses tobacco >1/week	790	Medium-high	**5.90%**	
India					774	Medium	**6.70%**	
					602	Medium-low	**7.90%**	
					206	Low	**8.70%**	
						No adjustment described		

<u>Zaman et al. (2012)</u> - Funded by the Byrraju Foundation and the Initiative for Cardiovascular Health Research in Developing Countries

cross-sectional	moderate	Representative sample from	**Education (men)**	**Smoker**	1,311	High (primary or higher)	**39.50%**	<0.001
4,535		20 villages in rural Andhra Pradesh.		smokes regularly on most days for >1 year	895	Low (no formal education)	**57.70%**	
		Aged >30 years						
India								
			**Education (women)**	**Smoker**	1,074	High (primary or higher)	**1.20%**	<0.001
					1,255	Low (no formal education)	**8.50%**	
			**Ocupation (men)**	**Smoker**	434	Skilled*	**34.10%**	<0.001
					1,501	Unskilled	**51.30%**	
			**Ocupation (women)**	**Smoker**	88	Skilled*	**1.10%**	0.06
					872	Unskilled	**7.10%**	
				**skilled manual labour, owner of business/farmer office worker/non-proffesional or professional)*				
			**Income (men)**	**Smoker**	912	High (>2000 INR/month)	**13.20%**	<0.001
					717	Middle (1200-1999 INR/month)	**12.60%**	
					577	Low (0-1199 INR/month)	**14.50%**	
			**Income (women)**	**Smoker**	945	High (>2000 INR/month)	**4.20%**	<0.001
					550	Middle (1200-1999 INR/month)	**3.80%**	
					834	Low (0-1199 INR/month)	**7.10%**	
						No adjustment described		

<u>Gupta et al. (2012)</u> - Funded by the South Asian Society of Atherosclerosis and Thrombosis

cross-sectional	high	General population in	**Education**	**Tobacco use**	1,248	Low (0-10 years)	**24.30%**	
6,198		middle-class areas of 11		daily use of a tobacco product	2,956	Middle (11-15 years)	**14.40%**	

		cities, excluded house-			1,366	High (>15 years)	**19.00%**		
		bound, pregnant and those							
		likely to die within 6 months.	**Occupational Class**	**Tobacco use**	1,287	Low (4-5)	**16.10%**		
India		Aged 18-75 years	**(British Social Register,**		1,677	Middle (3 manual/non-manual)	**20.30%**		
			**housewife = husband)**		3,018	High (1-2)	**16.70%**		
			**SES (self-assessed)**	**Tobacco use**	374	Low (score 1-3)	**17.60%**		
					3,622	Middle (score 4-6)	**19.60%**		
					1,114	High (score 7-10)	**15.50%**		
						adjusted for age and sex			

Reddy et al. (2007) - Funded by the Indian Ministry of Health; WHO

cross-sectional-19,96	high	Industrial workers and their relatives from ten urban sites across India. Aged 20-69 years.							
			**Education (men)**	**Tobacco use**	1,611	High (postgraduate)	**1**	OR	<0.001
India				use of any tobacco product in previous 30 days	2,607	Middle-high (secondary-tertiary)	**1.3**	1.1-1.5	
					5,820	Midle-low (primary-secondary)	**1.9**	1.6-2.2	
					1,859	Low (none-primaryl)	**6.5**	5.2-8.1	
			**Education (women)**	**Tobacco use**	960	High (postgraduate)	**1**	OR	<0.001
					1,635	Middle-high (secondary-tertiary)	**1.1**	0.8-1.3	
					2,832	Midle-low (primary-secondary)	**1.1**	0.76-1.4	
					2,645	Low (none-primaryl)	**8.2**	6.4-9.9	
						adjusted for age and occupation			

Singh et al. (2007) - Funded by the Center of Nutrition Research, International College of Nutrition (India)

cross-sectional 2,222	high	Spouses/relatives surveyed about their deceased from 1999-2001, who lived in Moradabad. Aged 25-64 years	**SES (men) education,**	**Tobacco consumption**	264	Highest	**58.00%**		>0.05*
India			**occupation, income, assets, housing**	use of tobacco product	345	High	**51.00%**		
					290	Middle	**47.60%**		
					277	Low	**42.60%**		
					209	Lowest	**51.20%**		

			**SES (men) education, occupation, income, assets, housing**	**Tobacco consumption** use of tobacco product	163	Highest	**13.50%**		<0.05*
					221	High	**15.40%**		
					169	Middle	**11.20%**		
					159	Low	**19.50%**		
*comparing highest-middle vs low and lowest					125	Lowest	**21.60%**		

No adjustment described

Kar et al. (2010) - Source of funding not reported

cross-sectional 400	moderate	Residents at eight sites in Northern India. Aged >30 years	**Literacy**	**Current tobacco user**	226	Literate	**0.3**	0.1-0.8	0.01
India				any tobacco use at least once per day for past month	177	Illiterate	**1**	OR	

adjusted for age, sex, literacy, place of residence

Lal and Nair (2012) - Source of funding not reported

cross-sectional	moderate	Data from India's 2009/10	**SES (housing, assets, services)**	**Tobacco use**		Low	**1**	OR	<0.01
233		Global Adult Tobacco				Middle	**1.25**	1.24-1.25	
		Survey. Young Keralan men.				High	**0.51**	0.51-0.52	
		Aged 15-24 years							
India			**Education**	**Tobacco use**		Low (less than secondary)	**1.00**	OR	<0.01
						High (secondary and above)	**1.23**	1.23-1.24	
			**Occupation**	**Tobacco use**		Low (unemployed)	**1.00**	OR	<0.01
						Middle (student)	**18.87**	18.67-19.06	
						High (working)	**6.89**	6.82-6.96	

adjusted for age, place of residence, education, occupation, # members in household, belief that tobacco is harmful

Samuel et al. (2012) - Funded by the British Heart Foundation

cross-sectional 2,218	high	Young adults from population-based birth cohort in rural and urban areas of southern India. Aged 26-32 years	**Wealth (asset score)**	**Tobacco use**		Lowest (quintile 1)	**1**	OR	
India				current user		Low (quinile 2)	**0.6**	0.4-0.9	
						Middle (quintile 3)	**0.5**	0.3-0.7	
						High (quinile 4)	**0.5**	0.4-0.9	
						Highest (Quintile 5)	**0.4**	0.2-0.6	

				**Education**	**Tobacco use**		Low (0 years formal schooling)	**1**	OR	
							Middle-low (1-8 years)	**0.8**	0.5-1.4	
							Middle-high (9-12)	**0.5**	0.3-0.9	
							High (>12 years)	**0.2**	0.1-0.4	

adjusted for gender, place of residence, posessions score, adult educational status, paternal educational status

<u>Gupta et al. (2015)</u> - Funded by the South Asian Society of Atherosclerosis and Thrombosis

cross-sectional	6,198	high	General population in middle-class areas of 11 cities, excluded house-bound, pregnant and those likely to die within 6 months. Aged >20 years	**Education**	**Quit tobacco**	1,248	Low (0-10 years)	**1.60%**		0.139
India					quit for >1year having used for >1 year previously	2,956	Middle (11-15 years)	**2.80%**		
						1,366	High (>15 years)	**5.50%**		

adjusted for age and sex

<u>Safraj et al. (2012)</u> - No external funding

cross-sectional	74147	moderate	PROFILE cohort - rural Keralan adults. Aged >20 years	**SES (assets, services)**	**Smoking**	9,557	Highest	**13.50%**		
India						23,468	Upper Middle	**17.60%**		
						32,577	Lowe Middle	**24.90%**		
						8,545	Lowest	**30.30%**		

No adjustment described

<u>Jena et al. (2012)</u> - Source of funding not reported

ross-sectional	69,29	high	Smokers from 29 territories, from the nationally representative 2009 Indian Global Adult Tobacco Survey. Aged >15 years	**Occupation**	**Hardcore smoker***		Employee	**1**	OR	
India							Student	**2.40**	1.16-4.95	0.018
							Self-employed	**2.64**	1.28-5.45	0.008
							Homemaker	**2.49**	1.18-5.26	0.017
							Retired or unemployed	**2.24**	1.07-4.69	0.033
				**Education**	**Hardcore smoker***		Low (no formal education)	**1**	OR	
							Medium-low (primary incomplete	**0.96**	0.84-1.10	0.569
							Medium (primary but secondary i	**1.01**	0.90-1.11	0.900

**Proportion of daily smokers who have not attempted to quit in prev 12 months/ last attempt was <24h and no intention to quit in ne*
if at all, smokes within 30 mins of waking and knows smoking is harmful

					High (secondary and above)	**1.00**	0.85-1.19	0.990
					Multivarate logistic regression, variables not reported			
**Narayan et al. (1996)** - Funded by the Sitaram Bhartia Institute of Science and Research								
cross-sectional 13,558	high	Residents of Delhi. Aged 25-64 years	**Education (men)**	**Smoker**	Low (none)	**1.75**	1.52-2.02	
India				currently smoking or had smoked >100 cigarettes or beedis	Middle-low (primary)	**1.29**	1.12-1.48	
					Middle-low (primary)	**1.06**	0.94-1.19	
					Middle-high (secondary)	**0.84**	0.75-0.94	
					High (college)	1	OR	
			**Education (women)**	**Smoker**	Low (none)	**3.72**	2.66-4.82	
					Middle-low (primary)	**1.13**	0.79-1.63	
					Middle-low (primary)	**0.94**	0.59-1.50	
					Middle-high (secondary)	**0.44**	0.24-0.79	
					High (college)	1	OR	
			**Occupation (men)**	**Smoker**	High (I)	1	OR	
					Medium-high (II)	**1.15**	0.96-1.38	
					Medium-low (III)	**0.8**	0.63-1.02	
					Low (IV)	**1.39**	1.13-1.70	
			**Occupation (women)**	**Smoker**	High (I)	1	OR	
					Medium-high (II)	**1.47**	0.47-4.62	
					Medium-low (III)	**0.87**	0.32-2.41	
					Low (IV)	**1.91**	0.64-5.70	
adjusted for income, edu, marital status, religion, occupation, physical activity, leuisure activity, BMI, drinking status, meat intake, egg eating, vegitarian, family history CVD								
**Rani et al. (2003)** - Source of funding not reported								
cross-sectional 334,553 India	high	Data from the 1998/9 National Family Health Survey. Aged >15 years	**Household wealth (men) dwelling and assets**	**Smoke tobacco**	Highest quintile	1	OR	ref
					Second quintile	**1.53**		<0.001
					Middle quintile	**1.94**		<0.001
					Fourth quintile	**2.11**		<0.001
					Lowest quintile	**2.26**		<0.001
			**Household wealth (men) dwelling and assets**	**Chew tobacco**	Highest quintile	1	OR	ref
					Second quintile	**1.4**		<0.001
					Middle quintile	**1.55**		<0.001
					Fourth quintile	**1.69**		<0.001
					Lowest quintile	**1.93**		<0.001
			**Household wealth (women)**	**Smoke tobacco**	Highest quintile	1	OR	

**dwelling and assets**		Second quintile	**1.57**		<0.001
		Middle quintile	**2.68**		<0.001
		Fourth quintile	**3.26**		<0.001
		Lowest quintile	**4.32**		<0.001
**Household wealth (women) dwelling and assets**	**Chew tobacco**	Highest quintile	**1**	OR	
		Second quintile	**1.52**		<0.001
		Middle quintile	**1.92**		<0.001
		Fourth quintile	**2.15**		<0.001
		Lowest quintile	**2.58**		<0.001
**Education (men)**	**Smoke tobacco**	High (>11 years)	**1**	OR	
		Medium-high (6-10 years)	**1.84**		<0.001
		Medium-low (1-5 years)	**2.72**		<0.001
		Low (No formal education)	**3.17**		<0.001
**Education (men)**	**Chew tobacco**	High (>11 years)	**1**	OR	
		Medium-high (6-10 years)	**1.48**		<0.001
		Medium-low (1-5 years)	**1.86**		<0.001
		Low (No formal education)	**1.92**		<0.001
**Education (women)**	**Smoke tobacco**	High (>11 years)	**1**	OR	
		Medium-high (6-10 years)	**1.73**		>0.05
		Medium-low (1-5 years)	**2.82**		<0.05
		Low (No formal education)	**6.25**		<0.001
**Education (women)**	**Chew tobacco**	High (>11 years)	**1**	OR	
		Medium-high (6-10 years)	**2.05**		<0.001
		Medium-low (1-5 years)	**3.81**		<0.001
		Low (No formal education)	**4.97**		<0.001
**Caste (men)**	**Smoke tobacco**	High (Forward/general caste)	**1**	OR	
		Medium (Other backward caste)	**1.01**		>0.05
		Low (Scheduled caste)	**1.2**		<0.001
		Low (Scheduled tribe)	**1.05**		>0.05
**Caste (men)**	**Chew tobacco**	High (Forward/general caste)	**1**	OR	
		Medium (Other backward caste)	**1.07**		<0.05
		Low (Scheduled caste)	**1.12**		<0.05
		Low (Scheduled tribe)	**1.23**		<0.001
**Caste (women)**	**Smoke tobacco**	High (Forward/general caste)	**1**	OR	

							Medium (Other backward caste)	**1.09**		<0.001
							Low (Scheduled caste)	**1.34**		>0.05
							Low (Scheduled tribe)	**1.49**		<0.05
				**Caste (women)**	**Chew tobacco**		High (Forward/general caste)	**1**	OR	
							Medium (Other backward caste)	**1.14**		>0.05
							Low (Scheduled caste)	**1.62**		<0.001
							Low (Scheduled tribe)	**2.49**		<0.001
							adjusted for wealth, years of schooling, religon, caste, age, sex, urban/rural			
**<u>Heck et al. (2012)</u>** - Funded by US National Institutes of Health										
cross-sectional	19,934	high	Married Bangladeshi adults from a longitudinal arsenic study (HEALS). Age 18-75 years	**Education**	**Betel quid**		Low (no formal)	**2**	1.81-2.20	
Bangladesh					use with or without tobacco (82.5% use it with tobacco)		Middle (1-5 years)	**1.65**	1.49-1.82	
							High (>6 years)	**1**	OR	
			adjusted for gender, age, marital status, occupation, religion, land ownership, TV ownership, smoking pack years							
**<u>Mumu et al. (2014)</u>** - Source of funding not reported										
cross-sectional	374	moderate	Type 2 diabetics who had been diagnosed for >1 year from 9 health centres around Dhaka. Aged >20 years	**Education**	**Smoker (continues)**	112	Low (up to primary)	**6.30%**		0.54
Bangladesh					still smoking despite medical advice to quit	174	Medium (up to higher second)	**4.60%**		
						88	High (graduate and above)	**8.00%**		
							No adjustment described			
**<u>Goon and Bipasha (2014)</u>** - No external funding										
cross-sectional	400	moderate	Bus drivers in Dhaka. Aged 18-50	**Literacy**	**Smoker**	165	Literate	**1**	OR	
Bangladesh						235	Illiterate	**2.8**	1.2-6.13	
							No adjustment described			
**<u>Zaman et al. (2014)</u>** - No external funding										
cross-sectional		low	Men from the nationally	**Tobacco use***	**Education**	1,292	Non-smokers (no tobacco at all)	**35.40%**		<0.001

4,312		representative 2012 WHO		% with less than primary education	1,752	Smokers	**58.30%**		
Bangladesh		NCD risk factor survey. Recruited from urban and			655	Smokeless tobacco users	**59.20%**		
		rural areas in 62 districts.			313	Dual users	**65.10%**		
		Aged >25 years							
			**Tobacco use***	**Wealth**	1,292	Non-smokers (no tobacco at all)	**14.00%**		<0.001
				poorest quartile from principal	1,752	Smokers	**23.90%**		
				component analysis of household assets	655	Smokeless tobacco users	**22.60%**		
*smoked a tobaco product in the last 30 days - doesn't use any other form of tobacco					313	Dual users	**27.70%**		
						No adjustment described			

Kishore et al. (2013) - Source of funding not reported

cross-sectional	high	Data from Global Adult	**Education (India)**	**Hardcore smoker***	24,309,857	High (college and above)	**1**	OR - Binary logistic regression	
92,491		Tobacco Survey data from India 2009/10 (69,296 individuals), Thailand 2009 (20,566 individuals), Bangladesh 2009 (9,629 individuals).		odds of daily smoker becoming a hardcore smoker		Medium-high (higher second)	**0.98**	0.66-1.44	0.91
India, Thailand, Bangladesh		Aged >15 years				Medium-low (up to primary)	**0.96**	0.62-1.48	0.85
						Low (no formal education)	**1.1**	0.72-1.68	0.65
			**Wealth Index (India)** principal component analysis	**Hardcore smoker**	24,309,857	Highest	**1**	OR - Binary logistic regression	
						Lowest	**1.27**	0.9-1.79	0.17
						Second	**1.29**	0.92-1.81	0.14
						Middle	**1.17**	0.85-1.60	0.33
						Fourth	**1.08**	0.80-1.47	0.6
			**Education (Bangladesh)**	**Hardcore smoker**	3,651,921	High (college and above)	**1**	OR - Binary logistic regression	
						Medium-high (higher second)	**2.39**	0.83-6.95	0.11
						Medium-low (up to primary)	**1.9**	0.65-5.60	0.24
						Low (no formal education)	**2.26**	0.78-6.55	0.13
			**Wealth Index (Bangladesh)** principal component analysis	**Hardcore smoker**	3,651,921	Highest	**1**	OR - Binary logistic regression	
						Lowest	**3.15**	1.67-5.97	0
						Second	**2.68**	1.12-5.05	0
						Middle	**1.93**	1.07-3.50	0.03

							Fourth	**1.49**	0.83-2.67	0.18
				**Education (Thailand)**	**Hardcore smoker**	3,180,566	High (college and above)	**1**	OR - Binary logistic regression	
							Medium-high (higher second)	**0.9**	0.59-1.37	0.63
							Medium-low (up to primary)	**1.07**	0.70-1.63	0.76
							Low (no formal education)	**0.96**	0.51-1.79	0.89
				**Wealth Index (Thailand)** principal component analysis	**Hardcore smoker**	3,180,566	Highest	**1**	OR - Binary logistic regression	
							Lowest	**1.28**	0.92-1.77	0.14
							Second	**1.04**	0.75-1.43	0.83
**current daily smoking, no quit attempt in last 12 months or last quit was <24h, no intention to quit in next 12 months or not intereste*							Middle	**1**	0.75-1.33	0.98
first smoke within 30mins of waking, knowledge of harms							Fourth	**1.08**	0.8-1.45	0.63

No adjustment described

<u>Dhungana et al. (2014)</u> - Source of funding not reported

cross-sectional	406	high	Rural community in the Sindhuli district.	**Caste**	**Smoker**		High (Brahman/Chhetri)	**26.20%**		0.79
			Aged 20-50 years		Smoking until last 30 days befor interview		Middle (Adhibasi/Janajati)	**29.70%**		
Nepal							Low (Dalits)	**28.60%**		
				**Education**	**Smoker**		Low (no formal education)	**44.70%**		<0.001
							Middle-low (lower than pimary)	**17.20%**		
							Middle-high (primary)	**8.30%**		
							High (secondary and higher)	**18.20%**		
				**SES** (education, occupation, income)	**Smoker**		Lowest	**42.60%**		<0.001
							Middle-low	**27.70%**		
							Middle-high	**18.80%**		
							Highest	**0.00%**		

No adjustment described

<u>Chawla et al. (2010)</u> - No external funding

cross-sectional	240	low	Residents Pokhara valley, self administered questionnaire. Mean age 33.4y, SD 11.4 years	**Education**	**Smoker**		Low (illiterate)	**40%**		0.23
Nepal							Medium (higher secondary)	**34.80%**		
							High (Graduate)	**25%**		

No adjustment described

<u>Dewi et al. (2010)</u> - Funded by Provincial Health Office of Yogyakarta Special Regency, Indonesia

cross-sectional	moderate	Representative sample of	**Income (male) 15-35 years**	**Cigarettes**	Low (<mean income	**62%**	
3,285		residents of Yogyakarta.		at least one cigarette per day	High (> mean income)	**52%**	
Indonesia		Aged 15-75 years	**35-54 years**		Low (<mean income	**72%**	
					High (> mean income)	**67%**	
			**55-75 years**		Low (<mean income	**65%**	
					High (> mean income)	**50%**	
			**Income (female) 15-35 years**	**Cigarettes**	Low (<mean income	**2%**	
					High (> mean income)	**4%**	
			**35-54 years**		Low (<mean income	**0%**	
					High (> mean income)	**1%**	
			**55-75 years**		Low (<mean income	**3%**	
					High (> mean income)	**2%**	
					No adjustment described		

Taylor et al. (1996) - Funded by the International Network for Clinical Epidemiology; National Institute of Medical Research (Lagos)

cross-sectional	low	Survey of adults selected	**Income (men) 20-39 years**	**Smoker**	Low (<NGN 3000/year)	**33.00%**	>0.05
882		from the civil service, three research institutions and two communities.		'smoking habit'	High (>NGN 3000/year)	**39.70%**	
Nigeria		Aged >20 years					
			**Income (men) 40-59 years**	**Smoker**	Low (<NGN 3000/year)	**40.00%**	>0.05
					High (>NGN 3000/year)	**37.40%**	
			**Income (women) 20-39 years**	**Smoker**	Low (<NGN 3000/year)	**3.60%**	>0.05
					High (>NGN 3000/year)	**4.70%**	
			**Income (women) 40-59 years**	**Smoker**	Low (<NGN 3000/year)	**3.10%**	>0.05
					High (>NGN 3000/year)	**5.30%**	
					No adjustment described		

Bovet et al. (2002) - Funded by the Swiss National Science Foundation

cross-sectional 9,254	high	Rural community in the Sindhuli district.	**Education**	**Smoker**	Low (none)	**1**	OR
Tanzania		Aged 20-50 years		≥1 cigarette/day	Middle-low (primary)	**0.93**	0.622
					Middle-high (secondary)	**0.53**	<0.001
					High (tertiary)	**0.56**	<0.002
			**Wealth (asset score)**	**Smoker**	Lowest	**1**	OR

							Second	**0.87**	0.131	
							Third	**0.46**	<0.001	
							Fourth	**0.42**	<0.001	
							Fifth	**0.58**	0.001	
							Highest	**0.48**	<0.001	
							adjusted for age, sex, occucation, wealth			
<u>**Kebede (2002)**</u> - Funded by the Research and Publication Office of the Gondar College of Medical Sciences										
cross-sectional	181	low	University instructors from four colleges in north west Ethiopia	**Income**	**Ever smoker**	8	Low (ETB 472-500)	**1.02**	0.13-6.73	<0.01
Ethiopia			Age not reported		ever smoked a cigarette	57	Medium (ETB 501-1,000)	**1**	OR	
						116	High (ETB 1,001-1,000[sic])	**3.29**	1.54-7.09	
				**Education**	**Ever smoker**	17	Low (diploma)	**1.3**	0.3-5.56	<0.01
						37	Medium (BSc)	**1**	OR	
						127	High (MSc and above)	**2.97**	1.22-7.40	
							No adjustment described			
<u>**Sossa et al. (2013)**</u> - Funded by the Canadian International Development Agency										
cohort	208	moderate	Healthy adults sampled from a large city, small town and rural area.	**Education**	**Smoker**		High school	**14.50%**		
Benin			Aged 25-60 years		current or former smoker		Primary schooling	**17.20%**		
							No Schooling	**6.70%**		
				**Wealth (assets)**	**Smoker**		High	**11.50%**		
							Medium	**9.70%**		
							Low	**19.10%**		
							No adjustment described			
<u>**Owusu-Dabo et al. (2009)**</u> - Funded by Cancer Research UK; Institute of Clinical Research of the University of Nottingham										
cross-sectional		moderate	Nationally representative	**Education (men)**	**Cigarettes**	1,004	Low (illiterate)	**20.19%**		
6,285			sample, excludes		smokes now and has smoked >100 cigarettes	765	Middle-low (primary)	**27.19%**		
Ghana			institutionalised individuals.			4,206	Middle-high (secondary)	**39.64%**		
			Aged >14 years			283	High (tertiary)	**66.78%**		
				**Education (women)**	**Cigarettes**	794	Low (illiterate)	**0.30%**		
						557	Middle-low (primary)	**0.13%**		

					2,539	Middle-high (secondary)	**0.17%**		
					94	High (tertiary)	**0.00%**		
			**SES (men)**	**Cigarettes**	1,462	Low (none)	**24.83%**		
					1,262	Middle-low (radio)	**40.65%**		
					1,513	Middle (Telephone)	**39.52%**		
					1,694	Middle-high (TV)	**38.61%**		
					327	High (car)	**44.65%**		
			**SES (women)**	**Cigarettes**	1,099	Low (none)	**0.41%**		
					749	Middle-low (radio)	**0.07%**		
					1,096	Middle (Telephone)	**0.00%**		
					859	Middle-high (TV)	**0.24%**		
					181	High (car)	**0.24%**		
						No adjustment described			

<u>Hosey et al. (2014)</u> - Funded by Uniformed Services University of the Health Sciences

cross-sectional	moderate	WHO STEPwise survey using	**Household Income (men)**	**Tobacco use**	65	High (>$10,000)	**0.53**	0.24-1.19	0.429
1,638		randomly selected		daily use of cigarettes, pipes, cigars or smokeless tobacco	121	Medium ($5000-$10,000)	**0.79**	0.42-1.51	
FS Micronesia		households in Pohnpei.			298	Low (<$5000)	**1**	OR	
		Aged 25-64							
			**Household Income (women)**	**Tobacco use**	93	High (>$10,000)	**0.45**	0.22-0.96	0.78
					170	Medium ($5000-$10,000)	**0.75**	0.39-1.44	
					486	Low (<$5000)	**1**	OR	
			**Education (males)**	**Tobacco use**	99	High (Post-secondary >13years)	**0.54**	0.32-0.93	0.062
					183	Medium (Secondary, 9-12 years)	**0.7**	0.46-1.06	
					315	Low (Primary <9 years)	**1**	OR	
			**Education (women)**	**Tobacco use**	57	High (Post-secondary >13years)	**0.17**	0.04-0.76	0.042
					302	Medium (Secondary, 9-12 years)	**0.69**	0.45-1.06	
					568	Low (Primary <9 years)	**1**	OR	
						adjusted for age			

<u>Minh et al. (2007)</u> - Funded by the International Network of Demographic Evaluation of Populations (INDEPTH); Swedish Council for Social and Work Life Research

cross-sectional 1,984	high	Representative 2005 STEPS survey in Bavi district of northern Vietnam.	**Education**	**Smoker**		Low (<7 years)	**0.9**	0.6-1.4	

Vietnam			Aged 25-64 years				Middle (7-9 years)	**0.8**	0.6-1.2
							High (>9 years)	**1**	OR
				**SES** (local authority assessment and rice production)	**Smoker**		low	**2**	1.2-3.4
							middle	**1.4**	1.1-2
							high	**1**	OR
						adjusted for Sex, Age, Education, Occupation, and Economic Status			
**Tonstad et al. (2013)** - Funded by the National Institutes of Health; The Fogarty International Center									
cross-sectional	5,592	high	Data from smokers identified in the 2006	**Education**	**Quit tobacco**	4,727	Low (<6 years)	**1**	OR
Cambodia			National Tobacco Survey. Aged >18 years		not used tobacco products for >2 years among ever-users	840	High (>7 years)	**1.46**	1.01-2.13
				**Income**	**Quit tobacco**	3,919	Low (<US$1/day)	**1**	OR
						1,673	High (>US$1/day)	**1.39**	1.01-1.91
				**Ocupation**	**Quit tobacco**	616	None	**1**	OR
						93	Profeessional	**2.52**	1.27-5.01
						167	Technichal/Service	**1.32**	0.7-2.51
						467	Labour	**1.98**	1.1-3.56
						adjusted for age, demographics, health status characteristics			
**Ahmad et al. (2005)** - Funded by the Fogarty International Centre; National Institutes of Health (USA)									
cross-sectional	8,328	moderate	Data from the five biggest tribes taken from the 1990-1994 National Health Survey.	**Wealth (assets)**	**Smoker**	2,523	Low (assets)	**1**	OR
Pakistan			Aged >15 years		current smoker and has smoked >100 cigarettes/beddies	4,199	Middle (assets)	**1.03**	0.88-1.2
						1,606	High (assets)	**1.13**	0.92-1.39
				**Literacy**	**Smoker**	2,861	Literate	**1**	OR
						5,467	Illiterate	**0.69**	0.59-0.8
						adjusted for age, ethnicity, sex, literacy, urban/rural			
**Ali et al. (2006)** - Source of funding not reported									
cross-sectional	411	high	Men from a rural area of Sindh province.	**Education**	**Smoker**	104	High (>10 years)	**1**	OR
Pakistan			Aged >18 years		has smoked >100 cigarettes	95	Medium-high (6-10 years)	**1.1**	0.9-1.6
						92	Medium-low (1-5 years)	**1.1**	0.9-1.4

					120	Low (Illiterate)	**1.1**	0.9-1.5	
			**Income (average, individual)**	**Smoker**	98	Low (no income)	**1**	OR	
					114	Medium-low (<US$30/month)	**1.4**	1.0-2.1	
					151	Medium-high (US$30-60/month)	**1.4**	1.0-2.1	
					48	High (>US$60/month)	**1.7**	1.2-2.6	

adjusted for age, income, marital status

Channa and Khan (2014) - Source of funding not reported

case-control 233	low	114 male manipuri chewers (chewed mainpuri for 5-8 years) from Hyderabad and 119 matched controls. Aged 15-45 years	**Literacy**	**Mainpuri use**		Literate	**1**	OR	
Pakistan				1-2 packets/day (18g) using for 5-8 years		Illiterate	**4.225**		<0.01

No adjustment described

Abd-Elhady et al. (2007) - Source of funding not reported

cross-sectional 283	low	Diabetic patients attending Alf-Maskan outpatient clinic in East Cairo.	**Education**	**Smokes tobacco**	78	High (undefined)	**76.90%**		
Eypt		Age not reported		continues to smoke despite medical advice	160	Medium (undefined)	**72.50%**		
					45	Low (Illiterate)	**82.20%**		
			**Education**	**Quit smoking**	78	High (undefined)	**5.10%**		
					160	Medium (undefined)	**3.10%**		
					45	Low (Illiterate)	**4.40%**		

No adjustment described

Al Ali et al. (2011) - Funded by the National Institute on Drug Abuse; EU grant Mediterranean studies of Cardiovascular disease and Hyperglycaemia

cross-sectional 1,168	moderate	Representative sample of Aleppo residents from a	**Education**	**Smoker**	351	Low (<6 years)	**33.50%**	28.3-39.1	>0.05
Syrian Arab Republic		2006 survey.		past month cigarette or water pipe smoking	509	Medium (6-11 years)	**44.60%**	38.9-50.4	
		Aged >25 years			308	High (>12 years)	**35.10%**	30-40.6	
			**SES (education, assets, employment status, houshold income)**	**Smoker**	415	Low (tertile score)	**35.40%**	29.6-41.6	<0.05
					369	Medium (tertile score)	**39.40%**	34.4-44.7	
					384	High (tertile score)	**42.60%**	36.8-48.7	

				**Employment**	**Smoker**	624	Employed	**56.50%**	48.1-64.5	<0.05
						544	Unemployed	**23.50%**	19.1-28.5	
							No adjustment described			

Laux (2014) -Funded by National Institutes of Health (USA); University of Pittsburgh School of Medicine; National Center for Research (USA)

cross-sectional	1,355	moderate	Six communities in central and western Nicaragua. Aged 20-60 years	**Income (US$/dependent/day)**	**Ever smoker**		Extremely Low (<$1/day)	**1**	OR	
Nicaragua					ever smoked tobacco		Low ($1-2/day)	**1.1**	0.73-1.65	
							Higher ($>2/day)	**0.91**	0.54-1.53	
							adjusted for age, sex, living history			

Hosseinpoor et al. (2012) - Funded by WHO

cross-sectional		high	Reports data from 48 LMICs						Standard Error	
	213,807		taken from the 2003 World	**SES (men) assets, services**	**Smoker**	**Georgia**	Lowest	**50.10%**	6	
Global			Health Survey		daily or occasional tobacco smoker		Low	**59.00%**	4.3	
			Aged >18 years				Medium	**58.30%**	3.5	
							High	**63.40%**	4	
							Highest	**67.20%**	2.8	
				**SES (women) assets, services**	**Smoker**	**Georgia**	Lowest	**1.90%**	1.1	
					daily or occasional tobacco smoker		Low	**2.40%**	1.1	
							Medium	**4.80%**	1.8	
							High	**8.20%**	2.5	
							Highest	**11.60%**	2.6	
									Standard Error	
				**SES (men) assets, services**	**Smoker**	**Morocco**	Lowest	**40.30%**	4.7	
					daily or occasional tobacco smoker		Low	**34.00%**	3.8	
							Medium	**38.40%**	5.4	
							High	**31.20%**	4.8	
							Highest	**17.50%**	3.5	
				**SES (women) assets, services**	**Smoker**	**Morocco**	Lowest	**0.00%**	0	
					daily or occasional tobacco smoker		Low	**0.40%**	0.3	
							Medium	**0.00%**	0	
							High	**0.30%**	0.3	

			Highest	**0.00%**	0
					Standard Error
**SES (men) assets, services**	**Smoker** daily or occasional tobacco smoker	**Paraguay**	Lowest	**62.40%**	2.6
			Low	**47.90%**	2.6
			Medium	**43.80%**	2.8
			High	**28.40%**	2.5
			Highest	**33.10%**	2.9
**SES (women) assets, services**	**Smoker** daily or occasional tobacco smoker	**Paraguay**	Lowest	**17.90%**	1.9
			Low	**16.30%**	1.9
			Medium	**14.30%**	1.8
			High	**9.70%**	1.3
			Highest	**12.20%**	1.5
					Standard Error
**SES (men) assets, services**	**Smoker** daily or occasional tobacco smoker	**Phillipines**	Lowest	**67.90%**	2.22
			Low	**60.60%**	2.4
			Medium	**57.30%**	2.2
			High	**55.60%**	2.2
			Highest	**50.20%**	2.3
**SES (women) assets, services**	**Smoker** daily or occasional tobacco smoker	**Phillipines**	Lowest	**17.20%**	1.7
			Low	**14.40%**	1.4
			Medium	**12.10%**	1.2
			High	**12.10%**	1.3
			Highest	**8.80%**	1.3
					Standard Error
**SES (men) assets, services**	**Smoker** daily or occasional tobacco smoker	**Sri Lanka**	Lowest	**56.10%**	4.5
			Low	**49.50%**	3.6
			Medium	**45.50%**	3.1
			High	**38.00%**	2.3
			Highest	**29.90%**	3.9
**SES (women) assets, services**	**Smoker** daily or occasional tobacco smoker	**Sri Lanka**	Lowest	**6.40%**	1.9
			Low	**4.70%**	1.5
			Medium	**3.00%**	1
			High	**1.70%**	0.6

			Highest	**2.40%**	1
					Standard Error
**SES (men) assets, services**	**Smoker** daily or occasional tobacco smoker	**Swaziland**	Lowest	**19.90%**	6.4
			Low	**10.70%**	2.8
			Medium	**14.80%**	5.4
			High	**14.30%**	5.2
			Highest	**16.50%**	3.9
**SES (women) assets, services**	**Smoker** daily or occasional tobacco smoker	**Swaziland**	Lowest	**8.80%**	3.4
			Low	**1.70%**	0.9
			Medium	**0.20%**	0.2
			High	**4.10%**	1.6
			Highest	**2.30%**	2.3
					Standard Error
**SES (men) assets, services**	**Smoker** daily or occasional tobacco smoker	**Ukraine**	Lowest	**55.30%**	6
			Low	**48.80%**	4.5
			Medium	**53.80%**	4.4
			High	**57.90%**	4
			Highest	**54.90%**	4.3
**SES (women) assets, services**	**Smoker** daily or occasional tobacco smoker	**Ukraine**	Lowest	**7.70%**	1.8
			Low	**6.10%**	1.4
			Medium	**12.00%**	2.3
			High	**13.20%**	3
			Highest	**14.00%**	2.2
					Standard Error
**SES (men) assets, services**	**Smoker** daily or occasional tobacco smoker	**Bangladesh**	Lowest	**72.20%**	2.9
			Low	**4.10%**	2.9
			Medium	**57.60%**	2.8
			High	**48.60%**	2.7
			Highest	**44.20%**	2.6
**SES (women) assets, services**	**Smoker** daily or occasional tobacco smoker	**Bangladesh**	Lowest	**8.20%**	1.5
			Low	**6.00%**	1.2
			Medium	**8.30%**	1.5
			High	**5.90%**	1.6

					Standard Error
**SES (men) assets, services**	**Smoker** daily or occasional tobacco smoker	**Burkina Faso**	Lowest	**29.90%**	3.4
			Low	**25.80%**	2.8
			Medium	**21.60%**	2.5
			High	**17.50%**	2.5
			Highest	**26.20%**	3.5
**SES (women) assets, services**	**Smoker** daily or occasional tobacco smoker	**Burkina Faso**	Lowest	**12.60%**	2.4
			Low	**14.10%**	2.4
			Medium	**10.90%**	2.1
			High	**10.50%**	2.2
			Highest	**8.20%**	2.9
					Standard Error
**SES (men) assets, services**	**Smoker** daily or occasional tobacco smoker	**Chad**	Lowest	**22.80%**	4
			Low	**19.90%**	3.2
			Medium	**19.30%**	3.2
			High	**18.20%**	2.3
			Highest	**14.80%**	2
**SES (women) assets, services**	**Smoker** daily or occasional tobacco smoker	**Chad**	Lowest	**1.60%**	0.7
			Low	**5.10%**	2
			Medium	**2.70%**	1.1
			High	**3.80%**	2
			Highest	**3.60%**	1.2
					Standard Error
**SES (men) assets, services**	**Smoker** daily or occasional tobacco smoker	**Comoros**	Lowest	**39.00%**	8
			Low	**37.90%**	6.5
			Medium	**31.60%**	4.6
			High	**39.50%**	6
			Highest	**32.40%**	5.5
**SES (women) assets, services**	**Smoker** daily or occasional tobacco smoker	**Comoros**	Lowest	**38.30%**	8.7
			Low	**14.70%**	5.5
			Medium	**21.10%**	6.8
			High	**16.30%**	5.9
			Highest	**20.20%**	8.7

					Standard Error
**SES (men) assets, services**	**Smoker**	**Congo**	Lowest	**31.20%**	5.8
	daily or occasional tobacco smoker		Low	**28.60%**	4.8
			Medium	**17.70%**	4.5
			High	**10.70%**	3.4
			Highest	**9.90%**	4.2
**SES (women) assets, services**	**Smoker**	**Congo**	Lowest	**4.90%**	2.5
	daily or occasional tobacco smoker		Low	**0.90%**	0.5
			Medium	**2.90%**	1.8
			High	**1.40%**	0.7
			Highest	**0.30%**	0.3
					Standard Error
**SES (men) assets, services**	**Smoker**	**Cote d'Ivoire**	Lowest	**28.00%**	3
	daily or occasional tobacco smoker		Low	**21.30%**	2.8
			Medium	**22.00%**	2.8
			High	**19.50%**	2.8
			Highest	**18.00%**	33.1
**SES (women) assets, services**	**Smoker**	**Cote d'Ivoire**	Lowest	**3.40%**	1.6
	daily or occasional tobacco smoker		Low	**5.10%**	1.8
			Medium	**3.90%**	1.3
			High	**1.80%**	0.8
			Highest	**1.10%**	0.6
					Standard Error
**SES (men) assets, services**	**Smoker**	**Ethiopia**	Lowest	**5.30%**	2
	daily or occasional tobacco smoker		Low	**9.50%**	2.2
			Medium	**8.10%**	1.8
			High	**8.80%**	4.8
			Highest	**4.80%**	1.2
**SES (women) assets, services**	**Smoker**	**Ethiopia**	Lowest	**0.40%**	0.4
	daily or occasional tobacco smoker		Low	**1.20%**	0.6
			Medium	**0.70%**	0.4
			High	**0.60%**	0.4
			Highest	**0.10%**	0.1

					Standard Error
**SES (men) assets, services**	**Smoker** daily or occasional tobacco smoker	**Ghana**	Lowest	**21.50%**	3
			Low	**12.70%**	1.9
			Medium	**9.60%**	1.8
			High	**6.10%**	1.3
			Highest	**8.40%**	1.8
**SES (women) assets, services**	**Smoker** daily or occasional tobacco smoker	**Ghana**	Lowest	**2.60%**	0.7
			Low	**1.40%**	0.7
			Medium	**0.90%**	0.7
			High	**0.70%**	0.4
			Highest	**1.30%**	0.5
					Standard Error
**SES (men) assets, services**	**Smoker** daily or occasional tobacco smoker	**India**	Lowest	**46.70%**	3.2
			Low	**45.80%**	2.9
			Medium	**37.80%**	3.8
			High	**23.50%**	2.9
			Highest	**21.80%**	3.1
**SES (women) assets, services**	**Smoker** daily or occasional tobacco smoker	**India**	Lowest	**12.40%**	3.7
			Low	**8.60%**	1.8
			Medium	**8.40%**	1.7
			High	**4.30%**	1.1
			Highest	**3.10%**	1
					Standard Error
**SES (men) assets, services**	**Smoker** daily or occasional tobacco smoker	**Kenya**	Lowest	**33.10%**	5.4
			Low	**26.90%**	4
			Medium	**25.20%**	3.9
			High	**25.60%**	4
			Highest	**26.70%**	5.5
**SES (women) assets, services**	**Smoker** daily or occasional tobacco smoker	**Kenya**	Lowest	**3.20%**	0.9
			Low	**3.30%**	1
			Medium	**3.80%**	2.3
			High	**0.80%**	0.4
			Highest	**0.20%**	0.2

					Standard Error
**SES (men) assets, services**	**Smoker** daily or occasional tobacco smoker	**Lao People's**	Lowest	**77.10%**	2.9
			Low	**72.70%**	2.7
			Medium	**61.00%**	3.2
			High	**62.70%**	2.9
			Highest	**41.50%**	2.8
**SES (women) assets, services**	**Smoker** daily or occasional tobacco smoker	**Lao People's**	Lowest	**28.30%**	3.6
			Low	**17.80%**	2.4
			Medium	**12.70%**	2.1
			High	**5.00%**	1.3
			Highest	**1.80%**	0.8
					Standard Error
**SES (men) assets, services**	**Smoker** daily or occasional tobacco smoker	**Malawi**	Lowest	**40.90%**	3.2
			Low	**34.90%**	2.7
			Medium	**24.00%**	3.3
			High	**15.90%**	2.4
			Highest	**13.30%**	2.7
**SES (women) assets, services**	**Smoker** daily or occasional tobacco smoker	**Malawi**	Lowest	**9.50%**	1.5
			Low	**7.50%**	1.6
			Medium	**6.80%**	1.5
			High	**3.50%**	1.1
			Highest	**0.70%**	0.5
					Standard Error
**SES (men) assets, services**	**Smoker** daily or occasional tobacco smoker	**Mali**	Lowest	**27.30%**	3.2
			Low	**28.20%**	3.2
			Medium	**24.70%**	2.7
			High	**24.20%**	2.5
			Highest	**25.90%**	3.1
**SES (women) assets, services**	**Smoker** daily or occasional tobacco smoker	**Mali**	Lowest	**4.00%**	1.6
			Low	**3.80%**	1.6
			Medium	**3.50%**	1.3
			High	**2.90%**	1.1

			Highest	**0.50%**	0.5
					Standard Error
**SES (men) assets, services**	**Smoker** daily or occasional tobacco smoker	**Mauritania**	Lowest	**27.80%**	4.3
			Low	**22.30%**	4.3
			Medium	**23.40%**	4
			High	**30.60%**	4.2
			Highest	**38.60%**	4
**SES (women) assets, services**	**Smoker** daily or occasional tobacco smoker	**Mauritania**	Lowest	**2.80%**	1.1
			Low	**1.60%**	0.8
			Medium	**5.60%**	2.2
			High	**7.20%**	1.8
			Highest	**5.80%**	1.5
					Standard Error
**SES (men) assets, services**	**Smoker** daily or occasional tobacco smoker	**Myanmar**	Lowest	**52.50%**	3.9
			Low	**53.70%**	3.2
			Medium	**46.00%**	2.8
			High	**48.40%**	2.5
			Highest	**40.30%**	2.5
**SES (women) assets, services**	**Smoker** daily or occasional tobacco smoker	**Myanmar**	Lowest	**21.80%**	2.5
			Low	**18.20%**	2.3
			Medium	**12.50%**	1.5
			High	**8.50%**	1.1
			Highest	**4.60%**	1
					Standard Error
**SES (men) assets, services**	**Smoker** daily or occasional tobacco smoker	**Nepal**	Lowest	**43.70%**	2.6
			Low	**36.80%**	2.5
			Medium	**36.00%**	2.2
			High	**30.40%**	2.2
			Highest	**26.00%**	2
**SES (women) assets, services**	**Smoker** daily or occasional tobacco smoker	**Nepal**	Lowest	**28.50%**	2.1
			Low	**25.60%**	2
			Medium	**18.70%**	1.5
			High	**17.90%**	1.8

			Highest	**9.70%**	1.3
					Standard Error
**SES (men) assets, services**	**Smoker** daily or occasional tobacco smoker	**Pakistan**	Lowest	**40.50%**	2.5
			Low	**35.40%**	2.5
			Medium	**35.60%**	2.7
			High	**32.00%**	2.6
			Highest	**19.10%**	2.1
**SES (women) assets, services**	**Smoker** daily or occasional tobacco smoker	**Pakistan**	Lowest	**7.40%**	1.5
			Low	**6.80%**	1.3
			Medium	**7.40%**	1.8
			High	**6.30%**	1.6
			Highest	**3.80%**	1.2
					Standard Error
**SES (men) assets, services**	**Smoker** daily or occasional tobacco smoker	**Senegal**	Lowest	**28.90%**	4.6
			Low	**25.50%**	4.7
			Medium	**24.40%**	4
			High	**21.20%**	4.1
			Highest	**26.30%**	3.8
**SES (women) assets, services**	**Smoker** daily or occasional tobacco smoker	**Senegal**	Lowest	**4.70%**	2
			Low	**0.00%**	0
			Medium	**0.40%**	0.4
			High	**0.50%**	0.4
			Highest	**1.90%**	1.4
					Standard Error
**SES (men) assets, services**	**Smoker** daily or occasional tobacco smoker	**Vietnam**	Lowest	**66.90%**	3.5
			Low	**59.80%**	4.3
			Medium	**43.00%**	5.6
			High	**45.00%**	4.2
			Highest	**46.70%**	4.7
**SES (women) assets, services**	**Smoker** daily or occasional tobacco smoker	**Vietnam**	Lowest	**3.20%**	1.2
			Low	**2.10%**	0.8
			Medium	**2.20%**	0.8
			High	**3.40%**	1.4

				Highest	**1.80%**	1.2
						Standard Error
	**SES (men) assets, services**	**Smoker** daily or occasional tobacco smoker	**Zambia**	Lowest	**36.80%**	3.5
				Low	**27.50%**	2.6
				Medium	**21.50%**	2.5
				High	**22.90%**	2.7
				Highest	**13.00%**	2.1
	**SES (women) assets, services**	**Smoker** daily or occasional tobacco smoker	**Zambia**	Lowest	**11.50%**	1.6
				Low	**7.90%**	2.3
				Medium	**2.60%**	1
				High	**3.40%**	1.2
				Highest	**3.90%**	1.5
						Standard Error
	**SES (men) assets, services**	**Smoker** daily or occasional tobacco smoker	**Zimbabwe**	Lowest	**37.30%**	4.5
				Low	**29.60%**	3.9
				Medium	**26.40%**	3.7
				High	**20.00%**	2.8
				Highest	**23.50%**	2.8
	**SES (women) assets, services**	**Smoker** daily or occasional tobacco smoker	**Zimbabwe**	Lowest	**6.40%**	2
				Low	**3.60%**	1
				Medium	**3.10%**	0.8
				High	**1.90%**	0.7
variables; age, marital status, education, employment, urban/rural				Highest	**1.70%**	0.8
				No adjustment described		

Search strategy

EMBASE search strategy Restrictions: published post 1990	
1	cardiovascular disease/ or heart disease/ or vascular disease/ or cerebrovascular disease/
2	exp ischemic heart disease/ or exp myocardial disease/
3	Heart Failure/
4	cerebrovascular accident/
5	non insulin dependent diabetes mellitus/
6	chronic obstructive lung disease/
7	exp *Neoplasm/
8	((cardiovascular or cardio-vascular) adj3 disease*).ti,ab.
9	((cardiovascular or cardio-vascular) adj3 (event* or outcome* or risk*)).ti,ab.
10	((coronary or heart or myocard*) adj3 disease*).ti,ab.
11	((coronary or heart or myocard*) adj3 (event* or outcome* or risk*)).ti,ab.
12	((ischaemic or ischemic or ischaemia or ischemia) adj3 disease*).ti,ab.
13	((ischaemic or ischemic or ischaemia or ischemia) adj3 (event* or outcome* or risk*)).ti,ab.
14	myocardial infarct*.ti,ab.
15	((cerebrovascular or vascular) adj3 disease*).ti,ab.
16	((cerebrovascular or vascular) adj3 (event* or outcome* or risk*)).ti,ab.
17	stroke.ti,ab.
18	heart failure.ti,ab.
19	diabet*.ti.
20	((type 2 or type ii or noninsulin dependent or non insulin dependent or adult onset or maturity onset or obes*) adj2 diabet*).ti,ab.
21	(niddm or t2dm or tiidm).ti,ab.
22	(chronic adj2 (lung or pulmonary)).ti,ab.
23	copd.ti,ab.
24	(neoplas* or cancer* or carcinoma* or tumor* or tumour* or malignan* or leukaemia or leukemia or lymphoma?).ti,ab.
25	1 or 2 or 3 or 4 or 5 or 6 or 7 or 8 or 9 or 10 or 11 or 12 or 13 or 14 or 15 or 16 or 17 or 18 or 19 or 20 or 21 or 22 or 23 or 24
26	Poverty/
27	socioeconomics/ or economic aspect/
28	Income/ or lowest income group/
29	gross national product/
30	Economic development/
31	salary/
32	poverty.ti,ab.
33	((socioeconomic or socio-economic or economic) adj2 (factor? or inequalit* or indicator? or status or development)).ti,ab.
34	((household? or house-hold? or family or families) adj3 (income or earning? or wage? or poor or wealth)).ti,ab.
35	(gross domestic product or gross national product or gdp or gnp).ti,ab.

36	(unemploy* or (employment adj2 (status or indicator? or level?))).ti,ab.
37	26 or 27 or 28 or 29 or 30 or 31 or 32 or 33 or 34 or 35 or 36
38	Developing Country.sh.
39	(Afghanistan or Angola or Armenia or Armenian or Bangladesh or Benin or Bhutan or Bolivia or Burkina Faso or Burkina Fasso or Upper Volta or Burundi or Urundi or Cambodia or Khmer Republic or Kampuchea or Cameroon or Cameroons or Cameron or Camerons or Cape Verde or Central African Republic or Chad or Comoros or Comoro Islands or Comores or Mayotte or Congo or Zaire or Cote d'Ivoire or Ivory Coast or Djibouti or French Somaliland or East Timor or East Timur or Timor Leste or Egypt or United Arab Republic or El Salvador or Eritrea or Ethiopia or Gabon or Gabonese Republic or Gambia or Gaza or Georgia Republic or Georgian Republic or Ghana or Gold Coast or Guatemala or Guinea or Guinea-Bisau or Guam or Guiana or Guyana or Haiti or Honduras or India or Maldives or Indonesia or Kenya or Kiribati or (Democratic People* adj2 Korea) or Kosovo or Kyrgyzstan or Kirghizia or Kyrgyz Republic or Kirghiz or Kirgizstan or Lao PDR or Laos or Lesotho or Basutoland or Liberia or Madagascar or Malawi or Nyasaland or Mali or Mauritania or Micronesia or Moldova or Moldovia or Moldovian or Mongolia or Morocco or Ifni or Mozambique or Myanmar or Myanma or Burma or Nepal or Netherlands Antilles or Nicaragua or Niger or Nigeria or Pakistan or Palestine or Papua New Guinea or Paraguay or Philippines or Philipines or Phillipines or Phillippines or Rwanda or Ruanda or Samoa or Samoan Islands or Navigator Island or Navigator Islands or Sao Tome or Senegal or Sierra Leone or Sri Lanka or Ceylon or Solomon Islands or Somalia or Sudan or Swaziland or Syria or Principe or South Sudan or Tajikistan or Tadzhikistan or Tadjikistan or Tadzhik or Tanzania or Timor-Leste or Togo or Togolese Republic or Uganda or Ukraine or Uzbekistan or Uzbek or Vanuatu or New Hebrides or Vietnam or Viet Nam or West Bank or Yemen or Zambia or Zimbabwe or Rhodesia).hw,kf,ti,ab,cp.
40	((developing or less* developed or under developed or underdeveloped or low* middle income or low* income or underserved or under served or deprived or poor*) adj (countr* or nation? or state? or population? or world)).ti,ab.
41	((developing or less* developed or under developed or underdeveloped or low* middle income or low* income) adj (economy or economies)).ti,ab.
42	(low* adj (gdp or gnp or gross domestic or gross national)).ti,ab.
43	(lmic or lami).ti,ab.
44	transitional countr*.ti,ab.
45	38 or 39 or 40 or 41 or 42 or 43 or 44
46	drinking behavior/
47	alcohol consumption/
48	exp alcoholic beverage/
49	(alcohol* adj5 (drink* or factor* or pattern* or habit* or consum* or unhealthy)).ti,ab.
50	(drink* adj5 (factor* or pattern* or habit* or consum* or unhealthy or bing*)).ti,ab.
51	(alcohol* or drink*).ti.
52	exp smoking/ or "tobacco use"/
53	(tobacco or smoking or smoke or smoker?).ti,ab.
54	feeding behavior/ or eating habit/ or food preference/ or portion size/
55	*diet/
56	exp obesity/
57	*food/ or fast food/ or fat/ or exp fruit/ or *vegetable/
58	carbonated beverage/ or energy drink/ or soft drink/ or sports drink/

**59**	((food or eating or diet*) adj5 (factor* or pattern* or habit* or consum* or unhealthy or healthy or healthful)).ti,ab.
**60**	((high* or low*) adj2 (fat? or salt? or sugar or carbohydrate?)).ti,ab.
**61**	((fat? or salt? or sodium or sugar? or carbohydrate?) adj5 (factor? or pattern? or habit? or consum* or eat? or eating)).ti,ab.
**62**	((carbonated or sugar* or fizzy) adj2 (drink* or beverage?)).ti,ab.
**63**	(((unhealthy or healthy or healthful) adj3 (fat? or oil?)) or transfat? or trans fat?).ti,ab.
**64**	((fruit? or vegetable? or fibre or fiber) adj5 (factor? or pattern? or habit? or consum* or eat? or eating)).ti,ab.
**65**	(junk food? or junkfood? or fast food? or fastfood? or snack*).ti,ab.
**66**	exp physical activity/
**67**	exp *exercise/
**68**	physical inactivity/
**69**	*lifestyle/ or sedentary lifestyle/
**70**	(physical* adj3 (fit* or activ* or inactiv*)).ti,ab.
**71**	inactivity.ti,ab.
**72**	(sedentary adj3 (lifestyle* or life style* or behavio*)).ti,ab.
**73**	exercise*.ti.
**74**	46 or 47 or 48 or 49 or 50 or 51 or 52 or 53 or 54 or 55 or 56 or 57 or 58 or 59 or 60 or 61 or 62 or 63 or 64 or 65 or 66 or 67 or 68 or 69 or 70 or 71 or 72 or 73
**75**	25 and 37 and 45 and 74

Piloted Screening Form

	**Criteria**	**Tick if yes**
1	Study is based in a World Bank listed Low- or Lower-Middle Income Country	◻
2	At least one indicator of poverty	◻
3	Data on one or more NCD behavioral risk factor (tobacco use, unhealthy diet, harmful alcohol use, physical inactivity)	◻
4	Primary research study (not a narrative review, editorial, book, opinion piece, letter, report, poster presentation or abstracts only	◻
**IF YES** to all above criteria, include study for full review:		**YES or NO**

Quality Scoring Rubric

Study type	1	2	3	4	5	6	7	8	**Total score** 1=achieved, 0=not achieved, 2=unclear [RCT
**Case-Control**	Is Case Definition Adequate?	Representativeness of the Cases	Selection of Controls	Definition of Controls	Comparability of cases and controls on basis of design/analysis	Ascertainment of Exposure	Non-Response Rate		
**Cohort**	Representativeness of the exposed cohort	Selection of the non-exposed cohort	Ascertainment of exposure	Demonstration that outcome of interest was not present at start of study	Comparability of cohorts on the basis of the design or analysis	Assessment of outcome	Was follow-up long enough for outcomes to occur	Adequacy of follow-up of cohorts	
**Cross-sectional**	Representativeness of the sample	Sample Size	Non-respondents	Ascertainment of the exposure	Comparability of subjects in different outcome groups (control for confounding)	Assessment of the outcome	Statistical test is appropriate		
**Interrupted time series**	shape of intervention prespecified (they say what they expect to happen)	Intervention independent of other changes/confounders/historic changes	Intervention did not affect data collection/data collection method same pre and post	Allocation concealment (blind or objective outcome assessment)	Incomplete data adequately addressed	all outcomes mentioned in methods are reported in results	free from other sources of bias e.g. seasonality		
**RCT**	Random sequence generation (selection bias)	Allocation concealment (selection bias)	Blinding of participants and personell (performance bias)	Blinding of outcome assessment (detection bias)	Incomplete oucome data (reporting bias)	Selective reporting (reporting bias)	Other bias		

score 1=achieved, 0=not achieved, 2=unclear [RCT only]

Section/topic	#	Checklist item	Reported on page #
**TITLE**			
Title	1	Identify the report as a systematic review, meta-analysis, or both.	1
**ABSTRACT**			
Structured summary	2	Provide a structured summary including, as applicable: background; objectives; data sources; study eligibility criteria, participants, and interventions; study appraisal and synthesis methods; results; limitations; conclusions and implications of key findings; systematic review registration number.	2
**INTRODUCTION**			
Rationale	3	Describe the rationale for the review in the context of what is already known.	3
Objectives	4	Provide an explicit statement of questions being addressed with reference to participants, interventions, comparisons, outcomes, and study design (PICOS).	4
**METHODS**			
Protocol and registration	5	Indicate if a review protocol exists, if and where it can be accessed (e.g., Web address), and, if available, provide registration information including registration number.	4
Eligibility criteria	6	Specify study characteristics (e.g., PICOS, length of follow-up) and report characteristics (e.g., years considered, language, publication status) used as criteria for eligibility, giving rationale.	4
Information sources	7	Describe all information sources (e.g., databases with dates of coverage, contact with study authors to identify additional studies) in the search and date last searched.	4
Search	8	Present full electronic search strategy for at least one database, including any limits used, such that it could be repeated.	Box 1
Study selection	9	State the process for selecting studies (i.e., screening, eligibility, included in systematic review, and, if applicable, included in the meta-analysis).	5
Data collection process	10	Describe method of data extraction from reports (e.g., piloted forms, independently, in duplicate) and any processes for obtaining and confirming data from investigators.	5
Data items	11	List and define all variables for which data were sought (e.g., PICOS, funding sources) and any assumptions and simplifications made.	5, Apdx 3
Risk of bias in individual studies	12	Describe methods used for assessing risk of bias of individual studies (including specification of whether this was done at the study or outcome level), and how this information is to be used in any data synthesis.	5
Summary measures	13	State the principal summary measures (e.g., risk ratio, difference in means).	5
Synthesis of results	14	Describe the methods of handling data and combining results of studies, if done, including measures of consistency (e.g., I^2) for each meta-analysis.	5

Section/topic	#	Checklist item	Reported on page #
Risk of bias across studies	15	Specify any assessment of risk of bias that may affect the cumulative evidence (e.g., publication bias, selective reporting within studies).	5
Additional analyses	16	Describe methods of additional analyses (e.g., sensitivity or subgroup analyses, meta-regression), if done, indicating which were pre-specified.	5
**RESULTS**			
Study selection	17	Give numbers of studies screened, assessed for eligibility, and included in the review, with reasons for exclusions at each stage, ideally with a flow diagram.	6
Study characteristics	18	For each study, present characteristics for which data were extracted (e.g., study size, PICOS, follow-up period) and provide the citations.	Tables 1-5, References
Risk of bias within studies	19	Present data on risk of bias of each study and, if available, any outcome level assessment (see item 12).	Tables 1-5
Results of individual studies	20	For all outcomes considered (benefits or harms), present, for each study: (a) simple summary data for each intervention group (b) effect estimates and confidence intervals, ideally with a forest plot.	Tables 1-5
Synthesis of results	21	Present results of each meta-analysis done, including confidence intervals and measures of consistency.	n/a
Risk of bias across studies	22	Present results of any assessment of risk of bias across studies (see Item 15).	n/a
Additional analysis	23	Give results of additional analyses, if done (e.g., sensitivity or subgroup analyses, meta-regression [see Item 16]).	7-11
**DISCUSSION**			
Summary of evidence	24	Summarize the main findings including the strength of evidence for each main outcome; consider their relevance to key groups (e.g., healthcare providers, users, and policy makers).	11-12
Limitations	25	Discuss limitations at study and outcome level (e.g., risk of bias), and at review-level (e.g., incomplete retrieval of identified research, reporting bias).	13
Conclusions	26	Provide a general interpretation of the results in the context of other evidence, and implications for future research.	11-12, 14
**FUNDING**			
Funding	27	Describe sources of funding for the systematic review and other support (e.g., supply of data); role of funders for the systematic review.	1

From: Moher D, Liberati A, Tetzlaff J, Altman DG, The PRISMA Group (2009). Preferred Reporting Items for Systematic Reviews and Meta-Analyses: The PRISMA Statement. PLoS Med 6(6): e1000097. doi:10.1371/journal.pmed1000097

For more information, visit: **www.prisma-statement.org**.

Extracted data variables

- Authors
- Reference
- Year
- Country
- Sample description
- Study design
- Age mean
- Age range
- Sex
- Sample frame size
- N included
- N responders/n with complete follow up information
- Source of funding
- Exposures and definition
- Subgroups
- Outcomes and definition
- Crude and adjusted results
- P values
- 95%Cis
- Adjusted variables
